# Copper mediated one-pot synthesis of quinazolinones and exploration of piperazine linked quinazoline derivatives as anti-mycobacterial agents†

**DOI:** 10.1039/d0ra08644d

**Published:** 2020-12-08

**Authors:** Satyaveni Malasala, Jitendra Gour, Md. Naiyaz Ahmad, Srikanth Gatadi, Manjulika Shukla, Grace Kaul, Arunava Dasgupta, Y. V. Madhavi, Sidharth Chopra, Srinivas Nanduri

**Affiliations:** National Institute of Pharmaceutical Education and Research (NIPER) Hyderabad 500037 India nanduri.niperhyd@gov.in nandurisrini92@gmail.com; Division of Microbiology, CSIR-Central Drug Research Institute Sitapur Road, Sector 10, Janakipuram Extension Lucknow-226031 Uttar Pradesh India skchopra.007@cdri.res.in skchopra007@gmail.com

## Abstract

A facile method was developed for the synthesis of quinazolinone derivatives in a one-pot condensation reaction *via in situ* amine generation using ammonia as the amine source and with the formation of four new C–N bonds in good to excellent yields. With the optimised method, we synthesized a library of piperazine linked quinazoline derivatives and the synthesized compounds were evaluated for their inhibitory activity against *Mycobacterium tuberculosis*. The compounds 8b, 8e, 8f, 8m, 8n and 8v showed potent anti-mycobacterial activity with MIC values of 2–16 μg mL^−1^. All the synthesized compounds follow Lipinski's rules for drug likeness.

## Abstract 2

A facile method was developed for the synthesis of quinazolinone derivatives in a one-pot condensation reaction *via in situ* amine generation using ammonia as the amine source and with the formation of four new C–N bonds in good to excellent yields.

## Graphical abstract

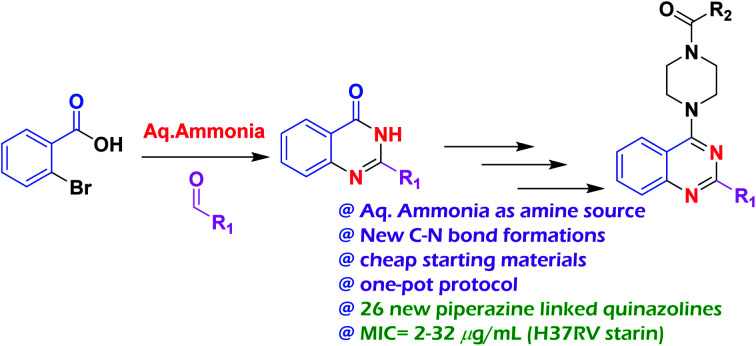

## Introduction

Nitrogen-containing heterocycles are present in a wide range of bioactive natural products and synthetic drug candidates.^1^ Among them, quinazolines and their derivatives represent medicinally important structural cores present in a number of drug candidates.^2^ They possess a wide range of biological activities including anticancer,^3^ antiviral,^4^ antitubercular^5^ and antibacterial^6^ properties.

Recently, ammonia has attracted wide attention as a cost-effective and efficient nitrogen source.^7^ A number of homogeneous transition-metal catalysed reactions for the synthesis of organic amines using gaseous or liquid ammonia are reported.^8,9^ Owing to its safety and ease of handling, aqueous ammonia is even more attractive as a substrate.

**Scheme 1:**
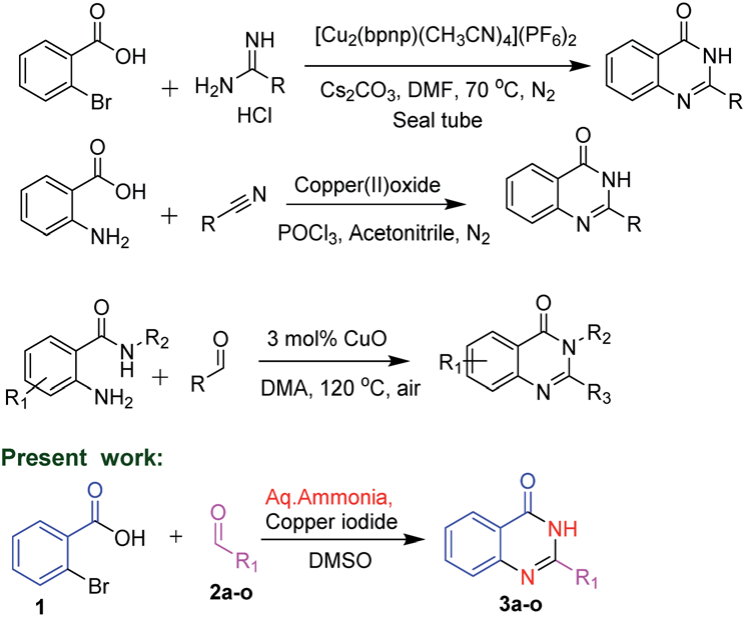
Methodologies for the synthesis of quinazolinones.

In view of the medicinal and pharmacological importance of quinazolinones, several methods on the synthesis of this class of compounds have been reported. Zhan and co-workers^10^ in 2013 reported an interesting approach by condensation of substituted anthranilamides with different aldehydes in presence of copper oxide for the synthesis of substituted quinazolinone derivatives. In 2014, Hung and co-workers^11^ reported synthesis of quinazolinone by using 2-bromobenzoic acid and substituted amidines as starting materials. Abe *et al.*^12^ reported the synthesis of quinazolinones by using 2-amino benzoic acid with substituted nitriles (Scheme 1).

**Fig. 1:**
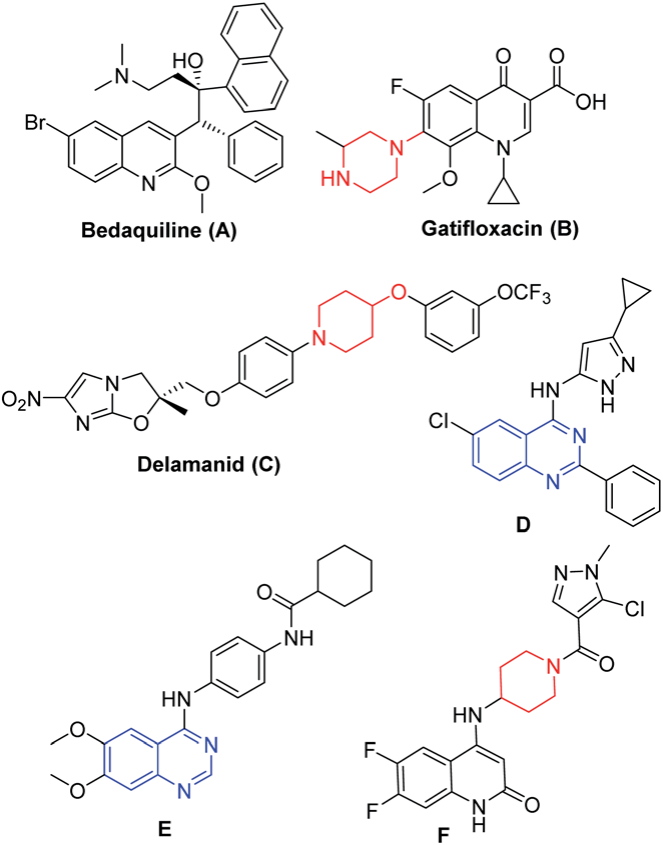
Structures of mycobacterial agents.

Tuberculosis is a transmissible disease caused by *Mycobacterium tuberculosis* (Mtb) complex and recognized to have high mortality rate globally^13^ According to WHO reports, 10 million cases were reported in the year 2017, where India is the leading country with the highest burden of TB.^14^ Emergence of drug-resistant TB or accompanying chronic diseases like HIV and diabetes certainly limits the current treatment options and hence drives the researchers to fulfil the growing demand for new agents (Fig. 1) that are effective against drug resistant TB.^15^ Bedaquiline (A, Fig. 1)^16^ led the drug discovery efforts towards the exploration of different heterocycles as anti-mycobacterial agents. Gatifloxacin (B, Fig. 1) and delamanid (C, Fig. 1) are the 2^nd^ line anti-TB drugs.^17*a*,*b*^ Wang *et al.* reported 4-(aminopyrazolyl)-substituted quinazolines (D, Fig. 1) as inhibitors of protein kinases (PknA & PknB) of *Mycobacterium tuberculosis*;^17*c*^ Tran *et al.* developed 4-aminoquinazolines (E, Fig. 1), as inhibitors of uridyl transferase activity of *M. tuberculosis* GlmU^17*d*^ and Naik *et al.* reported the quinolone based derivatives as potent anti-mycobacterial agents (F, Fig. 1).^17*e*^

In the present method, we have developed a copper mediated oxidative coupling of different aldehydes and 2-bromobenzoic acid, using aq. ammonia as a less expensive nitrogen source. We explored the double amination of aryl halides to the corresponding amines and also acids to amides at the same substrate. With the established method, we could successfully synthesize 4-substituted piperazine/piperidine linked C2-aryl/heteroaryl quinazolines. The synthesized compounds were evaluated for their *in vitro* inhibitory activity against *Mycobacterium tuberculosis* H37Rv. clog *P* values were determined using SwissADME.

**Scheme 2:**
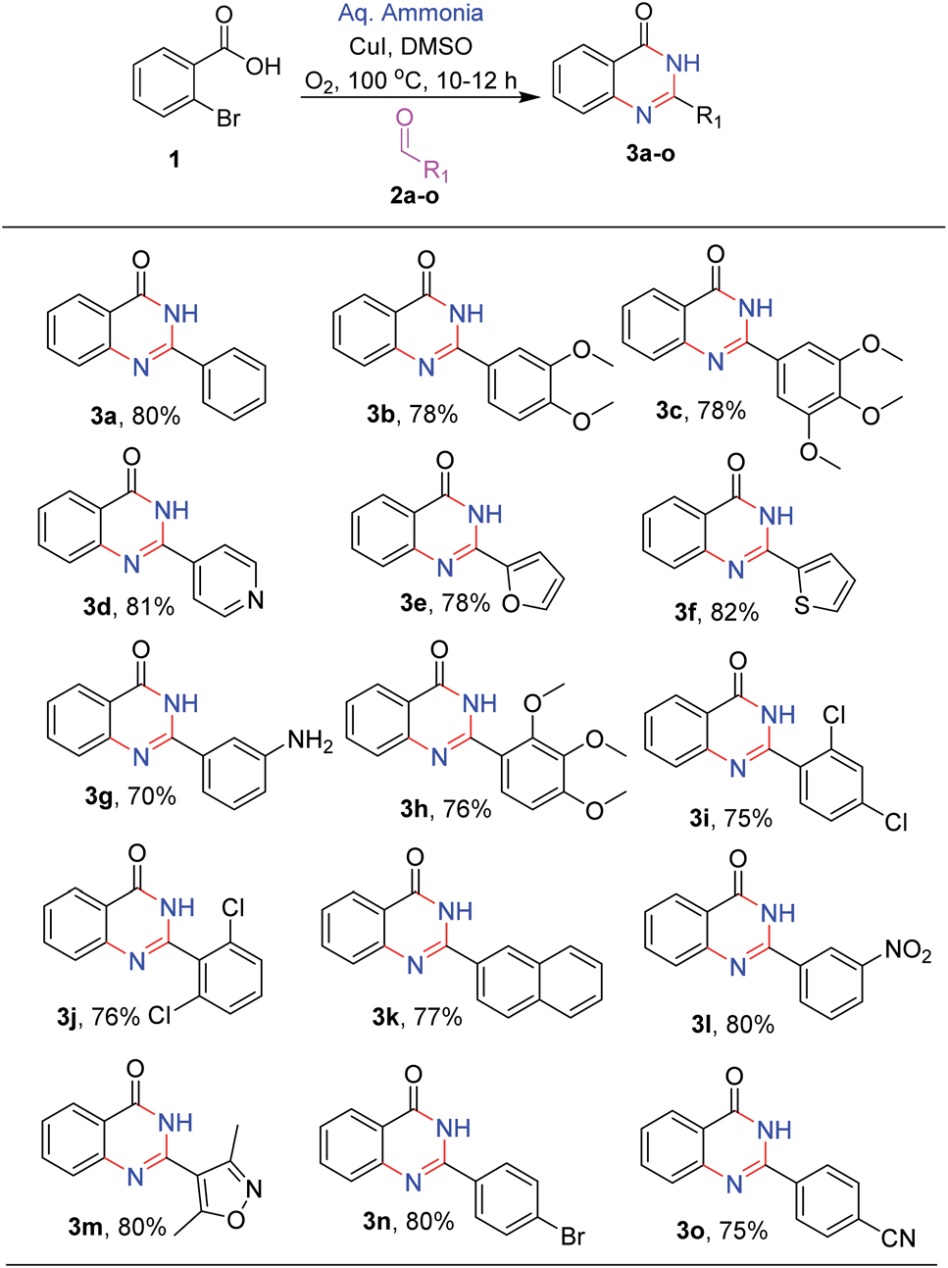
Reaction scope.

## Results and discussion

Direct employment of ammonia as a reagent in transition-metal catalysis is generally a challenging task. In the current optimised method, copper mediated oxidative coupling is developed between aldehydes and 2-bromobenzoic acid, using aq. ammonia as a nitrogen source. Various conditions for the conversion of 1 to 3 are studied and the results are discussed in Table 1. We initiated our studies by using copper oxide and NMP as solvent in the absence of oxygen at 80 °C for 24 h (Table 1, entry 1). The reaction did not proceed. However, we observed the formation of the product in small quantities when the reaction was conducted in presence of oxygen (Table 1, entry 2). With changes in time and temperature we observed the product formation to be improved (Table 1, entry 3–4). Moderate yields were observed with the change of catalyst to CuCl or CuBr with DMSO or NMP as solvents (Table 1, entry 5–8). With copper oxide and CuI as catalysts in presence of oxygen, NMP and DMSO as the solvent, the reaction proceeded smoothly. DMSO as a solvent was found to be more favourable (Table 1, entry 9–10). The reaction was studied with different solvents *viz.*, ACN, toluene, DMF and the reaction proceeded with altered yields (Table 1, entry 11–14). When the catalyst was changed to copper diacetate and coppertriflate the reaction was sluggish and resulted in low yields (Table 1, entry 15–17). The reaction was found to proceed with optimal yields with CuI as catalyst and DMSO as the solvent (entry 10).

**Table 1:** Optimization of reaction conditions^a^

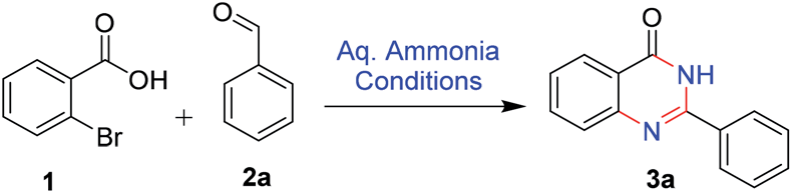						
Entry	Catalyst	Solvent	O_2_	Temperature (°C)	Time (h)	Yield (%)
1	Cu_2_O	NMP	No	80	24	0
2	Cu_2_O	NMP	Yes	80	24	12
3	Cu_2_O	NMP	Yes	80	12	25
4	Cu_2_O	NMP	Yes	100	10	40
5	CuCl	NMP	Yes	100	10	>20
6	CuCl	DMSO	Yes	100	10	24
7	CuBr	NMP	Yes	100	10	Trace
8	CuBr	DMSO	Yes	100	10	>20
9	Cu_2_O	DMSO	Yes	100	10	55
**10**	**CuI**	**DMSO**	**Yes**	**100**	**10**	**70**
11	CuI	ACN	Yes	100	10	Trace
12	CuI	Toluene	Yes	100	10	Trace
13	CuI	DMF	Yes	100	10	40
14	CuI	NMP	Yes	100	10	50
15	Cu(OAc)_2_	DMSO	Yes	100	10	38
16	Cu(OAc)_2_	DMF	Yes	100	12	26
17	Cu(OTf)_2_	DMSO	Yes	100	10	33

a Reaction conditions: 1 (1 mmol), aq. ammonia (2 mmol), aldehyde (1 mmol), catalyst (5.0 mol%), solvent (5 mL) the reaction was performed at 100 °C for 10 h under oxygen atmosphere.

After optimizing the reaction conditions, we focussed on expanding the substrate scope of this transformation and the results are summarized in (Scheme 2). It is observed that electron-donating substituents such as methoxy, amino, and methyl on 2-phenyl were well tolerated under the optimal reaction conditions, with 70–78% yields (3b, 3c, 3g and 3h). Similarly, halogen substituents like 2,4-dichloro, 2,6-dichloro and 3-nitro are also tolerated, yielding the desired products (3j, 3i and 3l) in good to high yields (75–79%). Heterocycles like pyridyl, furan, thiophene and isoxazole at C-2 position (3d, 3e, 3f and 3m) are also well tolerated with good to moderate yields (78–82%). With 4-bromo and 4-cyano (3o and 3n) substituents, the reactions proceeded smoothly (Scheme 2).

**Scheme 3:**
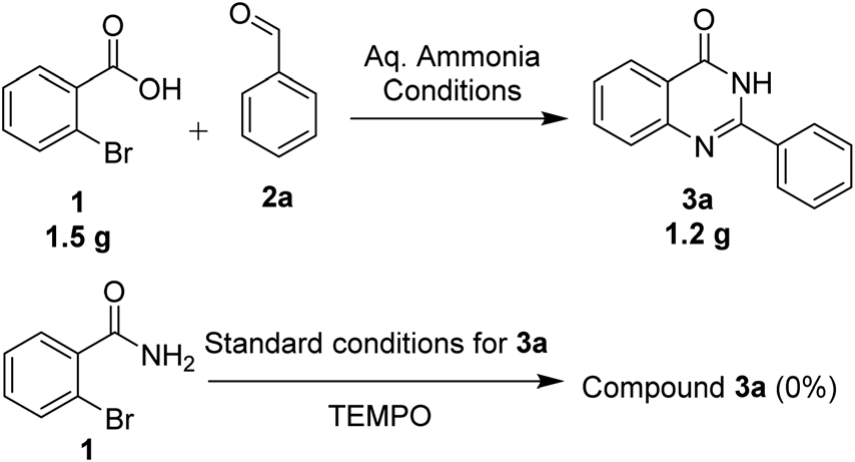
Gram-scale synthesis and control experiments.

**Scheme 4:**
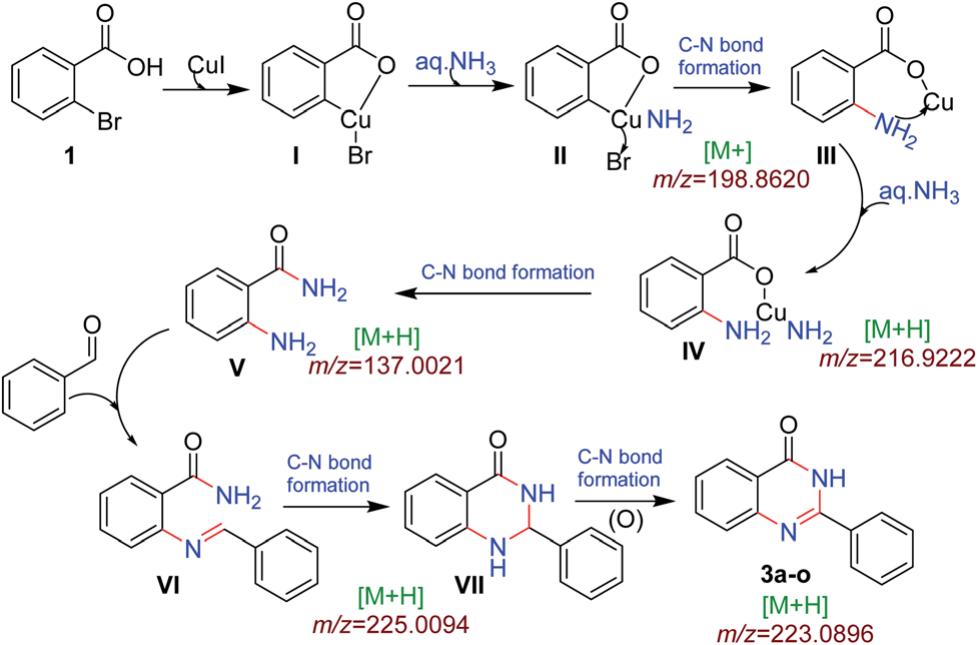
Plausible reaction mechanism.

**Fig. 2:**
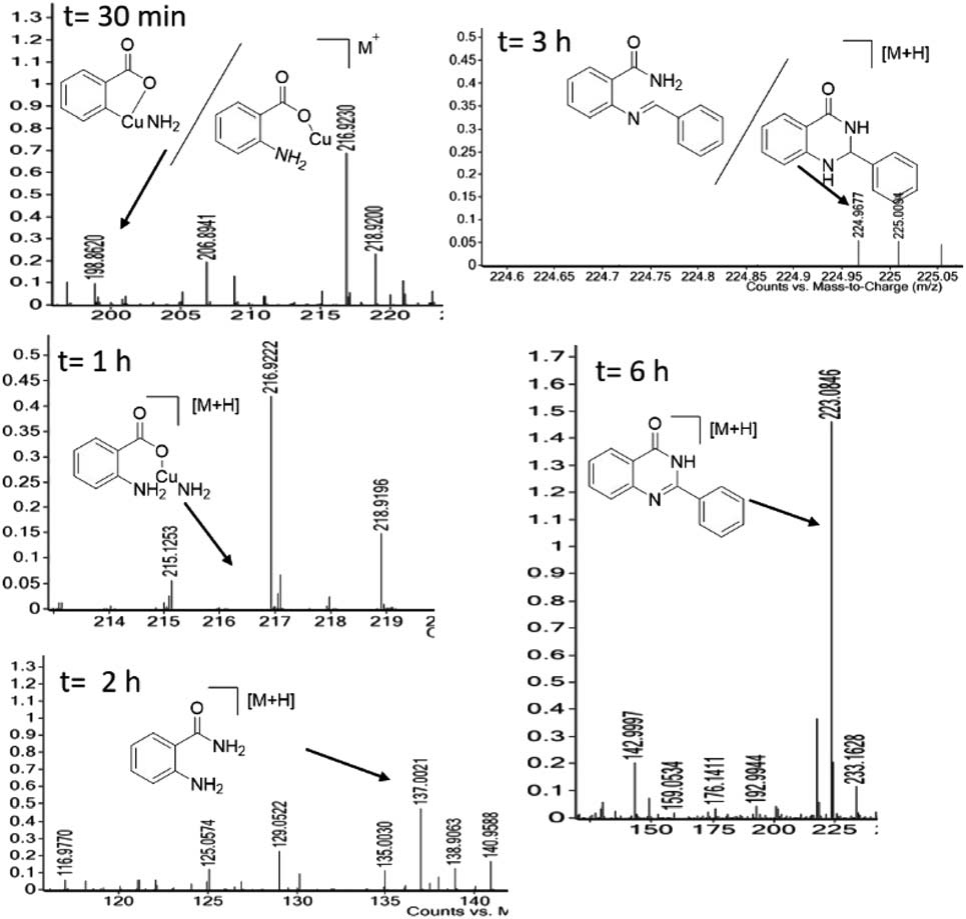
ESI-MS monitoring of the reaction.

We performed the gram scale synthesis with 1.5 gm of 2-bromo benzoic acid and benzaldehyde as the starting materials which resulted in 1.2 g of the final product (Scheme 3). As the reaction with TEMPO did not show the product formation, the free radical mediated mechanism is observed and conventional mechanism in one-pot protocol is proposed (Scheme 3). Based on the control experiments, a plausible reaction mechanism is proposed and depicted in Scheme 4. Our mechanistic investigation was supported by ESI-QTOF-MS technique and collected the mass data at different time intervals with <5 ppm error. Initially, under copper catalysis substrate 1 gets converted to intermediate I. Next addition of aq. ammonia gives the intermediate II, the observed mass was [M+] at *m*/*z* of 198.8620 after 30 min. The replacement of halo atom from the 2^nd^ position with amine will give the intermediate III, the obtained mass result was [M+] at *m*/*z* of 198.8620. After that another equivalent of aq. ammonia will be addition to the intermediate III to give the intermediate IV, the mass was shown with [M + H] at *m*/*z* of 216.9222, detected the peak after 1 h, which on further rearrangements gets converted to stable intermediate V, the mass was [M + H] at *m*/*z* of 137.0021 was observed after 2 h. Intermediate V on oxidation gets converted into imine intermediate VI which on addition of substituted aldehydes, the mass peak was observed with [M + H] at *m*/*z* of 225.0094, gives intermediate VII which finally on oxidation gives the desired products 3a–o in good to moderate yields, for the corresponding product the peak was observed with [M + H] at *m*/*z* of 223.0896. The product formation was observed after 6 h but not completely, further preceding the reaction for 10–12 h to get the complete conversion (Fig. 2).

**Scheme 5:**
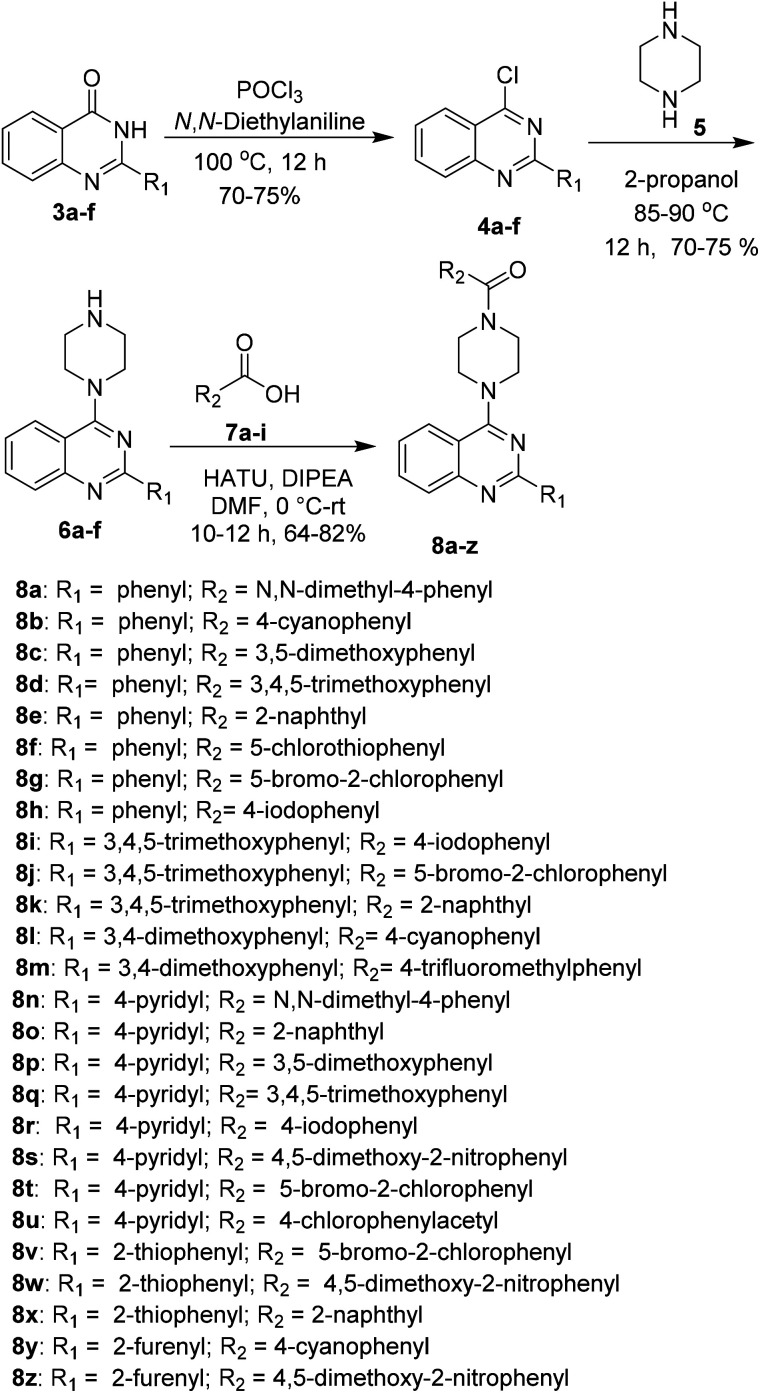
Synthetic route for the synthesis of piperazine linked quinazolines derivatives (8a–z).

A series of 2-arylquinazoline derivatives were synthesized with the optimized method as described in Scheme 2. The obtained quinazolinones (3a–f) were further chlorinated using POCl_3_ and *N*,*N*-diethyl aniline to provide the corresponding 2-aryl chloroquinazoline intermediates 4a–f. The chlorinated intermediates 4a–f were treated with piperazine 5 to yield 2-aryl-4-(piperazin-1-yl)quinazoline 6a–f. Coupling of 6a–f with a number of carboxylic acids 7a–i using HATU as coupling reagent afforded the corresponding amide derivatives 8a–z in moderate to excellent yields. Structures of all the newly synthesized compounds were confirmed by ^1^H NMR, ^13^C NMR and HRMS (ESI) spectroscopic techniques (Scheme 5).

**Fig. 3:**
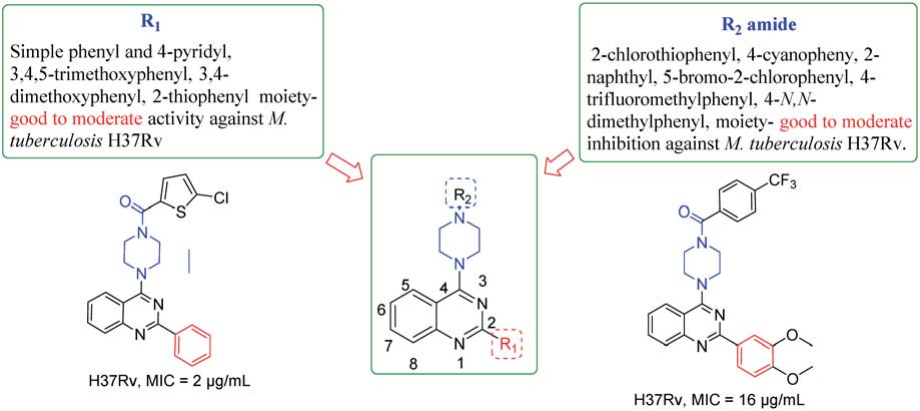
Structure activity relationship (SAR) of new 2-aryl/heteroaryl quinazoline derivatives.

The synthesized derivatives were evaluated for their antimicrobial activity against ESKAP pathogen panel (results included in the ESI†) and *Mycobacterium tuberculosis* H37Rv strain.^18–20^ While, the compounds were found to be inactive against ESKAP pathogenic panel, some of the compounds were found to exhibit promising inhibitory activity against *M. tuberculosis* H37Rv strain (Table 2). A perusal of the results indicated that among the amide derivatives, compounds 8f exhibited potent anti-mycobacterial activity with MIC of 2 μg mL^−1^ and 8n showed an MIC of 4 μg mL^−1^. Further, the results indicated that four compounds 8b, 8e, 8m and 8v showed MIC of 16 μg mL^−1^ whereas other molecules were found to be moderately active or inactive. Presence of methoxy group on 2-phenyl moiety as in 8k and 8m resulted in moderate activity with MIC of 16–64 μg mL^−1^. Replacement of C2-phenyl with 4-pyridyl was found to be a favourable lead. Substitution of R_2_ position with electron withdrawing groups like 2-chloro-5-bromo phenyl 8t showed inhibitory activity with MIC of 32 μg mL^−1^. Compounds having 2-phenyl with electron donating groups like 3,5-dimethoxy and 3,4,5-trimethoxy 8s and 8q were found to be inactive but 4-*N*,*N*-dimethylphenyl was found to be good lead with MIC of 2 μg mL^−1^. Compounds with halogen containing groups like 5-bromo-2-chlorophenyl 8y exhibited moderate activity with MIC of 32 μg mL^−1^ whereas 4-iodo 8r was inactive and unfortunately rest of the molecules were devoid of activity (Table 2 and Fig. 3).

**Table 2:** MIC (μg mL^−1^) values of 2-aryl/heteroaryl quinazoline based amide derivatives 8a–z against anti-bacterial and *M. tuberculosis* strains

Sample code	*S. aureus* ATCC 29213	*E. coli* ATCC 25922	*K. pneumoniae* BAA 1705	*A. baumannii* BAA 1605	*P. aeruginosa* ATCC 27853	Mtb H37Rv ATCC 27294	clog *P*
8a	>64	>64	>64	>64	>64	>64	3.84
8b	>64	>64	>64	>64	>64	**16**	3.65
8c	>64	>64	>64	>64	>64	>64	3.9
8d	>64	>64	>64	>64	>64	>64	3.77
8e	>64	>64	>64	>64	>64	**16**	4.77
8f	>64	>64	>64	>64	>64	**2**	4.49
8g	>64	>64	>64	>64	>64	>64	4.95
8h	>64	>64	>64	>64	>64	>64	4.54
8i	>64	>64	>64	>64	>64	>64	4.45
8j	>64	>64	>64	>64	>64	>64	4.92
8k	>64	>64	>64	>64	>64	**64**	4.7
8l	>64	>64	>64	>64	>64	>64	3.56
8m	>64	>64	>64	>64	>64	**16**	3.56
8n	>64	>64	>64	>64	>64	**4**	4.76
8o	>64	>64	>64	>64	>64	>64	3.13
8p	>64	>64	>64	>64	>64	>64	4.05
8q	>64	>64	>64	>64	>64	>64	3.14
8r	>64	>64	>64	>64	>64	>64	3.02
8s	>64	>64	>64	>64	>64	>64	3.76
8t	>64	>64	>64	>64	>64	**32**	2.25
8u	>64	>64	>64	>64	>64	>64	4.22
8v	>64	>64	>64	>64	>64	**16**	3.77
8w	>64	>64	>64	>64	>64	**64**	4.93
8x	>64	>64	>64	>64	>64	**64**	3.07
8y	>64	>64	>64	>64	>64	>64	2.99
8z	>64	>64	>64	>64	>64	>64	2.48
Levofloxacin	**0.125**	**0.015**	**64**	**8**	**0.5**	**Not tested**	
Isoniazid	—	—	—	—	—	**0.03**	
Rifampicin	—	—	—	—	—	**0.06**	

## Conclusions

In conclusion, we have developed an operationally simple, one-pot and cost-efficient method for the preparation of quinazolinones with diverse substituents. This method uses mild catalytic system which enables effective construction of four C–N bonds in one pot operation through *in situ* amine generation, confirmed through the ESI-MS technique. By using the optimised method, we generated a library of new piperazine linked 2-aryl/hetero-aryl-quinazoline derivatives which were evaluated for their anti-microbial activity against ESKAP pathogen panel and also against *M. tuberculosis*. Among the tested compounds, 8f exhibited selective and potent anti-mycobacterial activity with MIC value 2 μg mL^−1^. Compounds 8b, 8e, 8m and 8v exhibited moderate anti-mycobacterial activity with MIC value 16 μg mL^−1^. All the synthesized compounds obey the Lipinski rule of clog *P* values.

## Conflicts of interest

The authors declare no conflict of interest.

## Supplementary Material

RA-010-D0RA08644D-s001
