# Supplementary material for: Copper mediated one-pot synthesis of quinazolinones and exploration of piperazine linked quinazoline derivatives as anti-mycobacterial agents
Source: RSC Adv. 2020 Dec 8;10(71):43533–8. doi: 10.1039/d0ra08644d (PMC9058414; doi:10.1039/d0ra08644d)
Supplement: RA-010-D0RA08644D-s001 [file RA-010-D0RA08644D-s001.pdf]

Electronic Supplementary Material (ESI) for RSC Advances.
This journal is © The Royal Society of Chemistry 2020

Supporting Information

Copper mediated one-pot synthesis of quinazolinones and exploration of piperazine linked quinazoline derivatives as anti-mycobacterial agents

Satyaveni Malasala[a], Jitendra Gour[a], Md. Naiyaz Ahmed[b], Srikanth Gatadi[a], Manjulika Shukla[b], Grace Kaul[b], Arunav Dasgupta[b], Y.V Madhavi[a], Sidharth Chopra[b], Srinivas Nanduri[a*].

[a]*Department of Medicinal Chemistry, National Institute of Pharmaceutical Education and Research (NIPER), Hyderabad 500037, India*

[b]*Division of Microbiology, CSIR-Central Drug Research Institute, Sitapur Road, Sector 10, Janakipuram Extension, Lucknow-226031, Uttar Pradesh, India*

Contents

1.0 Anti-bacterial screening results Page no. 2 - 5

2.0 Experimental section Page no. 5-16

3.0 Copies of spectra Page no. 17 – 56

1.0 Anti-bacterial screening results

Table 1. Anti-bacterial screening results (MIC: μg/mL) of 2-aryl/heteroaryl quinazoline based amide derivatives **8a-z**.

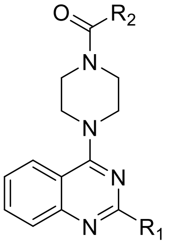

8a-z

S.no	Compound	R_1	R_2	*E.coli* ATCC 25922	*S.aureus* ATCC 29213	*K.pneumoniae* BAA 1705	*A.baumannii* BAA 1605	*P.aeruginosa* ATCC 27853
1.	**8a**	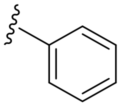	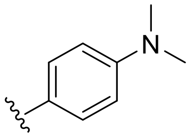	>64	>64	>64	>64	>64
2.	**8b**	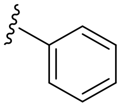	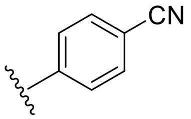	>64	>64	>64	>64	>64
3.	**8c**	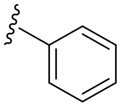	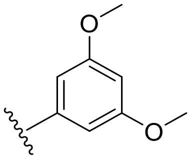	>64	>64	>64	>64	>64
4.	**8d**	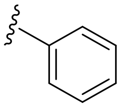	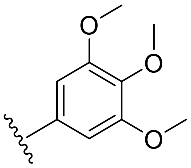	>64	>64	>64	>64	>64
5.	**8e**	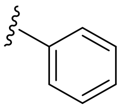	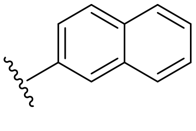	>64	>64	>64	>64	>64
6.	**8f**	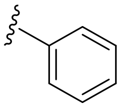	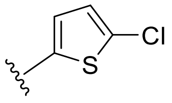	>64	>64	>64	>64	>64

7.	**8g**	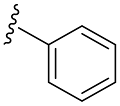	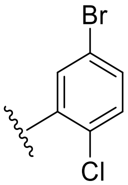	>64	>64	>64	>64	>64
8.	**8h**	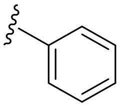	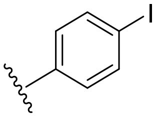	>64	>64	>64	>64	>64
9.	**8i**	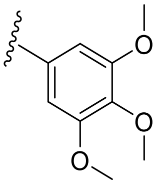	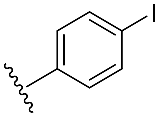	>64	>64	>64	>64	>64
10.	**8j**	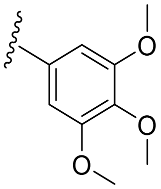	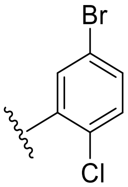	>64	>64	>64	>64	>64
11.	**8k**	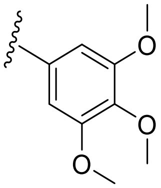	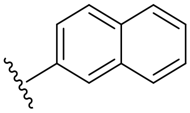	>64	>64	>64	>64	>64
12.	**8l**	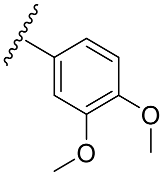	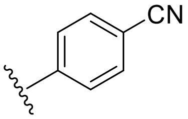	>64	>64	>64	>64	>64
13.	**8m**	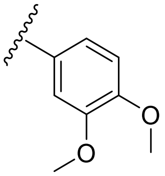	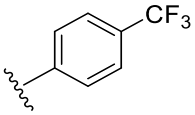	>64	>64	>64	>64	>64
14.	**8n**	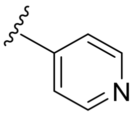	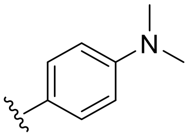	>64	>64	>64	>64	>64
15.	**8o**	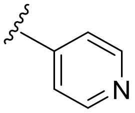	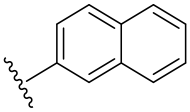	>64	>64	>64	>64	>64

16.	**8p**	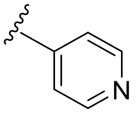	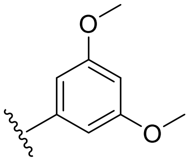	>64	>64	>64	>64	>64
17.	**8q**	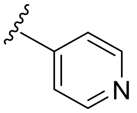	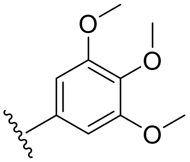	>64	>64	>64	>64	>64
18.	**8r**	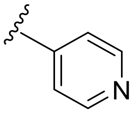	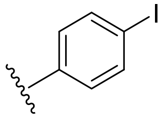	>64	>64	>64	>64	>64
19.	**8s**	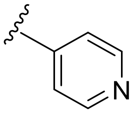	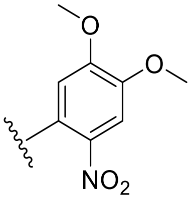	>64	>64	>64	>64	>64
20.	**8t**	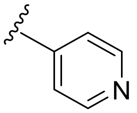	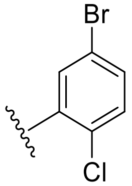	>64	>64	>64	>64	>64
21.	**8u**	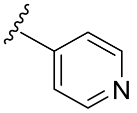	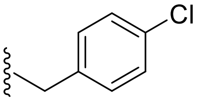	>64	>64	>64	>64	>64
22.	**8v**	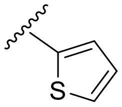	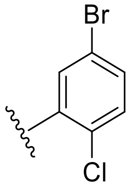	>64	>64	>64	>64	>64
23.	**8w**	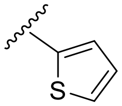	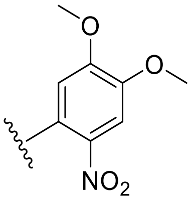	>64	>64	>64	>64	>64
24.	**8x**	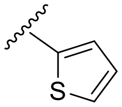	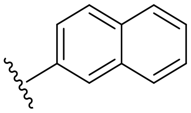	>64	>64	>64	>64	>64
25.	**8y**	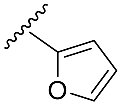	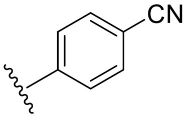	>64	>64	>64	>64	>64

26.	**8z**	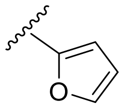	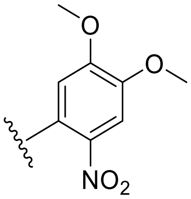	>64	>64	>64	>64	>64

2 Experimental section

2.1 General Methods. All the reagents and solvents were obtained from commercial suppliers and were used without further purification. Analytical thin layer chromatography (TLC) was performed on MERCK pre-coated silica gel 60-F254 (0.5 mm) aluminum plates. Visualization of the spots on TLC plates was achieved by UV light. ^{1}H and ^{13}C NMR spectra were recorded on Bruker 500 MHz by making a solution of samples in the DMSO-d_6 as solvent using tetramethyl silane (TMS) as the internal standard. Chemical shifts for ^{1}H and ^{13}C are reported in parts per million (ppm) downfield from tetra methyl silane. Spin multiplicities are described as s (singlet), brs (broad singlet), d (doublet), t (triplet), q (quartet), and m (multiplet). Coupling constant (J) values are reported in hertz (Hz). HRMS were determined with Agilent QTOF mass spectrometer 6540 series instrument. Wherever required, column chromatography was performed using silica gel (60-120). The reactions wherever anhydrous conditions required are carried under nitrogen positive pressure using freshly distilled solvents. All evaporation of solvents was carried out under reduced pressure using rotary evaporator below 45 ^{o}C. Melting points were determined with an electro thermal digital melting point apparatus IA9100 and are uncorrected. The names of all the compounds given in the experimental section were taken from ChemBioDraw Ultra, Version 12.0.

2.1.1 General Experimental Procedure for the Synthesis of substituted quinazolinones (3a-o): A solution of the 2-bromo benzoic acid (1 mmol), copper oxide (0.5 mmol) and aqueous ammonia (5 mmol) was taken in 5 mL of DMSO under oxygen atmosphere at room temperature. The reaction was then stirred for 2 h at 100 ^{o}C. Then substituted benzaldehyde (1 mmol) was added and progress of the reaction was monitored by TLC. After completion of the reaction, the suspension was extracted with ethyl acetate (3x5.0 mL), washed with 1:1 mixture of brine. The combined organic extracts were dried over anhydrous sodium sulphate. After removal of the solvent under

reduced pressure, the crude product was purified by using column chromatography, EtOAc:Hexane (2:8) as eluent on silica gel to afford the pure products.

*2.1.1.1 2-phenylquinazolin-4(3H)-one (**3a**)*

White solid; yield 80 %; mp:125-129 °C; ^{1}H NMR (500 MHz, DMSO-d_6): δ 12.56 (s, 1H), 8.33–8.08 (m, 3H), 7.90–7.81 (m, 1H), 7.80–7.72 (m, 1H), 7.67–7.47 (m, 4H); ^{13}C NMR (125 MHz, DMSO-d_6): δ 161.2, 150.3, 149.4, 149.1, 148.9, 138.4, 135.1, 128.2, 127.7, 127.0, 126.5, 122.6; HRMS (ESI): m/z calculated for $C_{14}H_{10}N_2O$ 223.0871 found 223.0898 [M+H]+.

2.1.1.2 2-(3,4-dimethoxyphenyl)quinazolin-4(3H)-one (***3b***)

White solid; yield 78 %; mp:125–129 °C; ^{1}H NMR (500 MHz, DMSO-d_6): δ 12.44 (s, 1H), 8.16-8.10 (m, 1H), 7.90-7.82 (m, 1H), 7.86–7.78 (m, 2H), 7.72 (d, J = 8.0 Hz, 1H), 7.55–7.45 (m, 1H), 7.14 (t, J = 13.9 Hz, 1H), 3.89 (s, 3H), 3.86 (s, 3H); ^{13}C NMR (125 MHz, DMSO-d_6): δ 162.9, 152.3, 152.0, 149.4, 149.0, 134.9, 127.8, 126.5, 126.3, 125.2, 121.6, 121.2, 111.8, 111.1, 56.1, 56.1; HRMS (ESI): m/z calculated for $C_{16}H_{14}N_2O_3$ 283.1083 found 283.1113 [M+H]+.

2.1.1.3 2-(3,4,5-trimethoxyphenyl)quinazolin-4(3H)-one (***3c***)

White solid; yield 78 %; mp:126–130 °C; ^{1}H NMR (500 MHz, DMSO-d_6): δ 12.54 (s, 1H), 8.16 (d, J = 7.7 Hz, 1H), 7.84 (t, J = 7.2 Hz, 1H), 7.76 (d, J = 8.0 Hz, 1H), 7.70-7.60 (m, 2H), 7.52 (t, J = 7.4 Hz, 1H), 3.90 (s, 6H), 3.78 (s, 3H); ^{13}C NMR (125 MHz, DMSO-d_6): δ 162.8, 153.3, 152.2, 149.1, 140.7, 135.1, 128.1, 127.9, 126.9, 126.3, 121.3, 105.6, 60.6, 56.6; HRMS (ESI): m/z calculated for $C_{17}H_{16}N_2O_4$ 313.1188 found 313.1227 [M+H]+.

2.1.1.4 2-(pyridin-4-yl)quinazolin-4(3H)-one (***3d***)

White solid; yield 81 %; mp:126-130 °C; ^{1}H NMR (500 MHz, DMSO-d_6): δ 11.82 (s, 1H), 8.77 (d, J = 4.5 Hz, 1H), 8.53–8.40 (m, 1H), 8.21 (t, J = 13.9 Hz, 1H), 8.09-8.01 (m, 1H), 7.91–7.85 (m, 1H), 7.81 (d, J = 8.0 Hz, 1H), 7.72–7.64 (m, 1H), 7.58 (t, J = 7.4 Hz, 1H); ^{13}C NMR (125 MHz, DMSO-d_6): δ 161.2, 150.3, 149.4, 149.1, 148.9, 138.4, 135.1, 128.2, 127.7, 127.0, 126.5, 122.6, 122.5; HRMS (ESI): m/z calculated for $C_{13}H_9N_3O$ 224.0824 found 224.0852 [M+H]+.

2.1.1.5 2-(furan-2-yl)quinazolin-4(3H)-one (***3e***)

Off-white solid; yield 78 %; mp:135-139 °C; ^{1}H NMR (500 MHz, DMSO-d_6): δ 12.52 (s, 1H), 8.13 (d, J = 7.6 Hz, 1H), 8.01 (s, 1H), 7.82 (t, J = 7.2 Hz, 1H), 7.70 (d, J = 8.1 Hz, 1H), 7.64 (d, J = 3.4 Hz, 1H), 7.50 (t, J = 7.4 Hz, 1H), 6.76 (d, J = 1.7 Hz, 1H); ^{13}C NMR (125 MHz, DMSO-d_6): δ

162.0, 149.1, 147.0, 146.5, 144.4, 135.1, 127.7, 126.9, 126.4, 121.6, 114.9, 112.9; HRMS (ESI): m/z calculated for $C_{12}H_8N_2O_2$ 213.0664 found 213.0688 [M+H]+.

*2.1.1.6 2-(thiophen-2-yl)quinazolin-4(3H)-one (**3f**)*

Off-white solid; yield 82 %; mp:123-126 °C; ^{1}H NMR (500 MHz, DMSO-d_6): δ 12.67 (s, 1H), 8.24 (d, J = 3.3 Hz, 1H), 8.13 (d, J = 7.7 Hz, 1H), 7.95-7.90 (m, 1H), 7.81 (t, J = 7.2 Hz, 1H), 7.66 (d, J = 8.1 Hz, 1H), 7.49 (t, J = 7.4 Hz, 1H), 7.31–7.18 (m, 1H); ^{13}C NMR (125 MHz, DMSO-d_6): δ 162.2, 149.1, 148.3, 137.8, 135.1, 132.6, 129.8, 128.9, 127.4, 126.8, 126.4, 121.4; HRMS (ESI): m/z calculated for $C_{12}H_8N_2OS$ 229.0436 found 229.0469 [M+H]+.

*2.1.1.7 2-(3-aminophenyl)quinazolin-4(3H)-one (**3g**)*

Off-white solid; yield 70 %; mp:123–127 °C; ^{1}H NMR (500 MHz, DMSO-d_6): δ 12.32 (s, 1H), 8.20–8.10 (m, 1H), 7.87–7.80 (m, 1H), 7.71 (d, J = 7.9 Hz, 1H), 7.55–7.48 (m, 1H), 7.40 (t, J = 1.9 Hz, 1H), 7.28 (d, J = 7.7 Hz, 1H), 7.17 (t, J = 7.8 Hz, 1H), 6.79-6.71 (m, 1H), 5.36 (s, 2H); ^{13}C NMR (125 MHz, DMSO-d_6): δ 162.6, 153.5, 149.4, 149.3, 135.0, 133.9, 129.5, 127.8, 126.8, 126.3, 121.4, 117.4, 115.4, 113.4. HRMS (ESI): m/z calculated for $C_{14}H_{11}N_3O$ 238.0980 found 238.1018 [M+H]+.

*2.1.1.8 2-(2,3,4-trimethoxyphenyl)quinazolin-4(3H)-one (**3h**)*

Off-white solid; yield 76 %; mp:125–128 °C; ^{1}H NMR (500 MHz, DMSO-d_6): δ 7.99-7.92 (m, 1H), 7.63 (d, J = 7.5 Hz, 1H), 7.28–7.17 (m, 2H), 6.85 (d, J = 8.7 Hz, 1H), 6.75 (d, J = 8.8 Hz, 2H), 6.68 (t, J = 7.4 Hz, 1H), 5.94 (s, 1H), 3.84 (s, 3H), 3.80 (s, 3H), 3.77 (s, 3H); ^{13}C NMR (125 MHz, DMSO-d_6): δ 164.2, 154.1, 151.4, 148.6, 141.8, 133.6, 127.8, 127.0, 122.6, 117.5, 115.3, 114.9, 108.2, 61.7, 60.8, 56.4; HRMS (ESI): m/z calculated for $C_{17}H_{16}N_2O_4$ 313.1188 found 313.1226 [M+H]+.

*2.1.1.9 2-(2,4-dichlorophenyl)quinazolin-4(3H)-one (**3i**)*

White crystalline solid; yield 75 %; mp:121-124 °C; ^{1}H NMR (500 MHz, DMSO-d_6): δ 12.68 (s, 1H), 8.27–8.10 (m, 1H), 7.92–7.86 (m, 1H), 7.84 (d, J = 2.0 Hz, 1H), 7.88-7.79 (m, 2H), 7.65–7.51 (m, 2H); ^{13}C NMR (125 MHz, DMSO-d_6): δ 161.9, 151.9, 148.9, 135.9, 135.1, 133.2, 133.2, 132.7, 129.6, 127.9, 127.7, 126.3, 121.8; HRMS (ESI): m/z calculated for $C_{14}H_8Cl_2N_2O$ 291.0092 found 291.0129 [M+H]+.

*2.1.1.10 2-(2,6-dichlorophenyl)quinazolin-4(3H)-one (**3j**)*

White crystalline solid; yield 76 %; mp:122–125 °C; ^{1}H NMR (500 MHz, DMSO-d_6): δ 12.82 (s, 1H), 8.26-8.20 (m, 1H), 7.90-7.86 (m, 1H), 7.75 (d, J = 7.9 Hz, 1H), 7.71–7.54 (m, 4H); ^{13}C NMR (125 MHz, DMSO-d_6): δ 161.9, 150.4, 148.9, 135.2, 133.7, 133.3, 132.7, 128.8, 127.9, 126.4, 121.9; HRMS (ESI): m/z calculated for $C_{14}H_8Cl_2N_2O$ 291.0092 found 291.0129 [M+H]+.

*2.1.1.11 2-(naphthalen-2-yl)quinazolin-4(3H)-one (**3k**)*

Off-white solid; yield 77 %; mp:122–125 °C; ^{1}H NMR (500 MHz, DMSO-d_6): δ 12.69 (s, 1H), 8.24 (d, J = 7.0 Hz, 1H), 8.19 (d, J = 6.7 Hz, 1H), 8.14 (d, J = 7.6 Hz, 1H), 8.06 (d, J = 6.1 Hz, 1H), 7.88 (s, 1H), 7.81 (d, J = 5.9 Hz, 1H), 7.75 (d, J = 7.2 Hz, 1H), 7.66 (d, J = 6.6 Hz, 1H), 7.60 (d, J = 5.4 Hz, 4H); ^{13}C NMR (125 MHz, DMSO-d_6): δ 162.4, 154.2, 149.2, 135.0, 133.6, 132.2, 130.8, 130.7, 128.8, 128.2, 127.9, 127.5, 127.3, 126.8, 126.3, 125.7, 125.5, 121.7; HRMS (ESI): m/z calculated for $C_{18}H_{12}N_2O$ 273.1028 found 273.1071 [M+H]+.

*2.1.1.12 2-(3-nitrophenyl)quinazolin-4(3H)-one (**3l**)*

Off-white solid; yield 80 %; mp:123–126 °C; HRMS (ESI): m/z calculated for $C_{14}H_9N_3O_3$ 268.0722 found 268.0754 [M+H]+.

*2.1.1.13 2-(3,5-dimethylisoxazol-4-yl)quinazolin-4(3H)-one (**3m**)*

White solid; yield 80 %; mp:120-123 °C; ^{1}H NMR (500 MHz, DMSO-d_6): δ 9.98 (s, 1H), 7.70–7.42 (m, 5H), 2.53 (s, 3H), 2.42 (s, 3H); ^{13}C NMR (125 MHz, DMSO-d_6): δ 186.3, 150.8, 145.8, 138.5, 129.8, 129.0, 125.6, 119.0, 13.1, 11.2; HRMS (ESI): m/z calculated for $C_{13}H_{11}N_3O_2$ 242.0930 found 242.0967 [M+H]+.

*2.1.1.14 2-(4-bromophenyl)quinazolin-4(3H)-one (**3n**)*

White solid; yield 80 %; mp:122-125 °C; ^{1}H NMR (500 MHz, DMSO-d_6): δ 8.20-8.10 (m, 2H), 8.06 (d, J = 8.4 Hz, 1H), 7.95 (d, J = 1.8 Hz, 1H), 7.68 (dd, J = 8.4, 1.8 Hz, 1H), 7.62 (t, J = 7.3 Hz, 1H), 7.56 (t, J = 7.4 Hz, 2H); ^{13}C NMR (125 MHz, DMSO-d_6): δ 162.3, 154.2, 150.2, 132.7, 132.2, 130.0, 129.9, 129.1, 128.6, 128.4, 120.5; HRMS (ESI): m/z calculated for $C_{14}H_9BrN_2O$ 300.9977 found 300.9952 [M+2]+.

*2.1.1.15 4-(4-oxo-3,4-dihydroquinazolin-2-yl)benzonitrile (**3o**)*

White solid; yield 75 %; mp:124-127 °C; ^{1}H NMR (500 MHz, DMSO-d_6): δ 8.41 (s, 1H), 7.67 (d, J = 16.3 Hz, 1H), 7.65-7.59 (m, 1H), 7.55 (d, J = 8.0 Hz, 1H), 7.50 (d, J = 7.8 Hz, 1H), 7.36 (t, J = 7.8 Hz, 1H), 7.30-7.24 (m, 1H), 7.23 (s, 1H), 6.77 (d, J = 8.1 Hz, 1H), 6.69 (t, J = 7.4 Hz, 1H), 5.79 (s, 1H); ^{13}C NMR (125 MHz, DMSO-d_6): δ 162.0, 149.1, 147.1, 146.6, 144.5, 135.1, 127.7,

126.9, 126.4, 121.6, 114.9, 112.9; HRMS (ESI): m/z calculated for $C_{15}H_9N_3O$ 248.0824 found 248.0857 [M+H]+.

2.1.2 General Experimental Procedure for the Synthesis of piperazine amide derivatives (8a-z);

To the mixture of substituted benzoic acids (**7a-i**, 1mmol) and HATU (1mmol), DMF (3mL) was added slowly under nitrogen atmosphere. The reaction mixture was then stirred for 20 minutes at 0 ^{o}C, followed by the addition of 2-phenyl-4-(piperazin-1-yl) quinazoline (**6a-h**, 1mmol). The reaction mixture was stirred for 20 minutes at room temperature, followed by the addition of DIPEA. Upon completion of the reaction as monitored by TLC, crushed ice was added to the reaction mixture. The resulting solid was then subjected to vacuum filtration, excess of water was used to wash off the insoluble solids to obtain crude powder which was purified using column chromatography (elution with hexane/EtOAc = 7:3). The pure products were collected as white color solids in good yields.

*2.1.2.1 (4-(dimethylamino)phenyl)(4-(2-phenylquinazolin-4-yl)piperazin-1-yl)methanone (**8a**)*

White solid; Yield 73 %; mp: 125-128 ^{o}C; ^{1}H NMR (500 MHz, DMSO-d_6): δ 8.57–8.46 (m, 2H), 8.08 (d, J = 8.2 Hz, 1H), 7.91 (d, J = 7.9 Hz, 1H), 7.84 (t, J = 7.2 Hz, 1H), 7.57–7.49 (m, 4H), 7.37 (d, J = 8.7 Hz, 2H), 6.74 (d, J = 8.8 Hz, 2H), 3.91 (s, 4H), 3.81 (s, 4H), 2.97 (s, 6H); ^{13}C NMR (125 MHz, DMSO-d_6): δ 170.5, 164.5, 158.5, 152.6, 151.7, 138.4, 133.4, 130.8, 129.6, 129.0, 128.8, 128.4, 125.9, 125.8, 122.3, 115.2, 111.5, 49.5, 40.2; HRMS (ESI): m/z calculated for $C_{27}H_{27}N_5O$ 438.2294 found 438.2324 [M+H]+.

*2.1.2.2 4-(4-(2-phenylquinazolin-4-yl)piperazine-1-carbonyl)benzonitrile (**8b**)*

White solid; Yield 73 %; mp: 123-126 ^{o}C; ^{1}H NMR (500 MHz, DMSO-d_6): δ 8.55–8.47 (m, 2H), 8.07 (d, J = 8.3 Hz, 1H), 7.97 (d, J = 8.4 Hz, 2H), 7.92 (d, J = 8.4 Hz, 1H), 7.85 (dd, J = 11.2, 4.1 Hz, 1H), 7.69 (d, J = 8.4 Hz, 2H), 7.57–7.47 (m, 4H), 4.02–3.80 (m, 6H), 3.59 (s, 2H); ^{13}C NMR (125 MHz, DMSO-d_6): δ 168.1, 164.5, 158.5, 152.5, 140.8, 138.4, 133.5, 133.0, 130.8, 128.9, 128.8, 128.5, 128.4, 125.9, 125.7, 118.8, 115.2, 112.6, 49.3; HRMS (ESI): m/z calculated for $C_{26}H_{21}N_5O$ 420.1824 found 420.1850 [M+H]+.

*2.1.2.3 (3,5-dimethoxyphenyl)(4-(2-phenylquinazolin-4-yl)piperazin-1-yl)methanone (**8c**)*

White solid; Yield 73 %; mp: 128-131 ^{o}C; ^{1}H NMR (500 MHz, DMSO-d_6): δ 8.53-8.49 (m, 2H), 8.07 (d, J = 8.2 Hz, 1H), 7.91 (d, J = 7.8 Hz, 1H), 7.84 (t, J = 7.2 Hz, 1H), 7.68–7.43 (m, 3H), 6.60

(s, 2H), 4.13–3.83 (m, 2H), 3.78 (s, 2H), 3.62 (s, 1H); ^{13}C NMR (125 MHz, DMSO-d_6): δ 170.5, 164.5, 158.5, 152.6, 151.7, 138.4, 133.4, 130.8, 129.6, 129.0, 128.8, 128.4, 125.9, 125.8, 122.3, 115.2, 111.5, 49.5, 40.2; HRMS (ESI): m/z calculated for $C_{27}H_{26}N_4O_3$ 455.2083 found 455.2116 [M+H]+.

*2.1.2.4 (4-(2-phenylquinazolin-4-yl)piperazin-1-yl)(3,4,5-trimethoxyphenyl)methanone (**8d**)*

White solid; Yield 73 %; mp: 125-128 °C; ^{1}H NMR (500 MHz, DMSO-d_6): δ 8.52 (s, 2H), 8.08 (d, J = 7.4 Hz, 1H), 7.91 (s, 1H), 7.85 (s, 1H), 7.61-7.47(s, 4H), 6.89-6.66 (s, 2H), 3.94 (s, 3H), 3.83 (s, 3H), 3.72 (s, 2H); ^{13}C NMR (125 MHz, DMSO-d_6): δ 169.5, 164.5, 158.5, 153.3, 152.5, 138.9, 138.4, 133.4, 131.6, 130.8, 130.7, 128.8, 128.6, 128.4, 125.9, 125.8, 115.1, 105.1, 60.5, 56.6, 49.4; HRMS (ESI): m/z calculated for $C_{28}H_{28}N_4O_4$ 485.2189 found 485.2210 [M+H]+.

*2.1.2.5 Naphthalen-2-yl(4-(2-phenylquinazolin-4-yl)piperazin-1-yl)methanone (**8e**)*

White solid; Yield 73 %; mp: 122-126 °C; ^{1}H NMR (500 MHz, DMSO-d_6): δ 8.51 (d, J = 3.5 Hz, 2H), 8.09 (s, 2H), 8.06–7.98 (m, 3H), 7.92 (d, J = 8.2 Hz, 1H), 7.85 (t, J = 7.4 Hz, 1H), 7.62 (d, J = 7.7 Hz, 3H), 7.58–7.44 (m, 4H), 3.85 (d, J = 125.6 Hz, 8H); ^{13}C NMR (125 MHz, DMSO-d_6): δ 169.8, 164.5, 158.5, 152.6, 138.4, 133.6, 133.6, 132.7, 130.84, 128.8, 128.5, 128.4, 128.1, 127.6, 127.2, 127.1, 125.9, 125.8, 125.0, 115.2, 49.5; HRMS (ESI): m/z calculated for $C_{29}H_{24}N_4O$ 445.2028 found 445.2060 [M+H]+.

*2.1.2.6 (5-chlorothiophen-2-yl)(4-(2-phenylquinazolin-4-yl)piperazin-1-yl)methanone (**8f**)*

White solid; Yield 73 %; mp: 134-138 °C; ^{1}H NMR (500 MHz, DMSO-d_6): δ 8.56–8.47 (m, 2H), 8.10 (d, J = 8.2 Hz, 1H), 7.92 (d, J = 8.2 Hz, 1H), 7.85 (t, J = 7.5 Hz, 1H), 7.60-7.48 (m, 4H), 7.45 (d, J = 3.9 Hz, 1H), 7.21 (d, J = 3.9 Hz, 1H), 4.05-3.85 (m, 8H); ^{13}C NMR (125 MHz, DMSO-d_6): δ 164.2, 161.7, 158.5, 152.6, 138.5, 137.2, 133.4, 132.7, 130.8, 130.1, 128.8, 128.4, 127.7, 125.8, 115.1, 49.0; HRMS (ESI): m/z calculated for $C_{23}H_{19}ClN_4OS$ 435.1046 found 435.1070 [M+H]+.

*2.1.2.7 (5-bromo-2-chlorophenyl)(4-(2-phenylquinazolin-4-yl)piperazin-1-yl)methanone (**8g**)*

White solid; Yield 73 %; mp: 133-136 °C; ^{1}H NMR (500 MHz, DMSO-d_6): δ 8.55-8.49 (m, 2H), 8.08 (d, J = 8.1 Hz, 1H), 7.92 (d, J = 7.7 Hz, 1H), 7.85 (t, J = 7.1 Hz, 1H), 7.75 (d, J = 8.6 Hz, 1H), 7.62 (d, J = 2.5 Hz, 1H), 7.57–7.46 (m, 5H), 4.05-3.91 (m, 6H), 3.46 (d, J = 4.2 Hz, 2H); ^{13}C NMR (125 MHz, DMSO-d_6): δ 165.7, 164.6, 158.5, 152.5, 140.0, 138.3, 134.8, 133.5, 133.4, 131.0, 130.8, 128.8, 128.4, 128.3, 126.0, 125.7, 117.4, 115.2, 49.4, 46.4; HRMS (ESI): m/z calculated for $C_{25}H_{20}BrClN_4O$ 507.0587 found 509.0565 [M+H]+.

*2.1.2.8 (4-iodophenyl)(4-(2-phenylquinazolin-4-yl)piperazin-1-yl)methanone (**8h**)*

White solid; Yield 73 %; mp: 129-132 °C; ^{1}H NMR (500 MHz, DMSO-d_6): δ 8.58–8.46 (m, 2H), 8.07 (d, J = 8.2 Hz, 1H), 7.92 (d, J = 8.2 Hz, 1H), 7.85 (t, J = 10.0 Hz, 1H), 7.60–7.46 (m, 4H), 7.30 (d, J = 8.2 Hz, 1H), 4.10-3.88 (m, 8H); ^{13}C NMR (125 MHz, DMSO-d_6): δ 169.0, 164.5, 158.5, 152.6, 138.4, 137.7, 135.6, 133.4, 130.8, 129.6, 128.8, 128.8, 128.4, 125.9, 125.7, 115.2, 96.9, 49.4; HRMS (ESI): m/z calculated for $C_{25}H_{21}IN_4O$ 521.0838 found 521.0864 [M+H]+.

*2.1.2.9 (4-iodophenyl)(4-(2-(3,4,5-trimethoxyphenyl)quinazolin-4-yl)piperazin-1-yl)methanone (**8i**)*

White solid; Yield 73 %; mp: 127-130 °C; ^{1}H NMR (500 MHz, DMSO-d_6): δ 8.16 (d, J = 7.8 Hz, 1H), 7.83 (d, J = 8.1 Hz, 3H), 7.76 (d, J = 8.0 Hz, 1H), 7.58 (s, 2H), 7.52 (t, J = 7.4 Hz, 1H), 7.23 (d, J = 7.7 Hz, 2H), 3.92 (s, 6H), 3.76 (s, 3H), 3.55-3.38 (m, 8H); ^{13}C NMR (125 MHz, DMSO-d_6): δ 168.9, 162.7, 153.3, 152.1, 149.1, 140.7, 137.7, 135.4, 135.0, 129.5, 128.1, 127.9, 126.9, 126.3, 121.3, 105.7, 60.6, 56.6; HRMS (ESI): m/z calculated for $C_{28}H_{27}IN_4O_4$ 611.1155 found 611.1189 [M+H]+.

*2.1.2.10 (5-bromo-2-chlorophenyl)(4-(2-(3,4,5-trimethoxyphenyl)quinazolin-4-yl)piperazin-1-yl)methanone (**8j**)*

White solid; Yield 73 %; mp: 126-129 °C; ^{1}H NMR (500 MHz, DMSO-d_6): δ 8.16 (d, J = 7.0 Hz, 1H), 7.84 (t, J = 7.0 Hz, 1H), 7.74 (d, J = 6.7 Hz, 1H), 7.69 (d, J = 8.5 Hz, 1H), 7.58 (s, 3H), 7.51 (d, J = 6.4 Hz, 1H), 7.47 (d, J = 8.6 Hz, 1H), 7.43 (d, J = 8.5 Hz, 1H), 3.91 (s, 6H), 3.70-3.50 (s, 4H), 3.62 (s, 3H), 3.28–3.08 (m, 4H); ^{13}C NMR (125 MHz, DMSO-d_6): δ 162.9, 153.3, 152.3, 149.1, 140.7, 135.0, 128.2, 127.8, 126.8, 126.3, 121.3, 105.7, 60.6, 56.6; HRMS (ESI): m/z calculated for $C_{28}H_{26}BrClN_4O_4$ 597.0904 found 599.0900 [M+2]+.

*2.1.2.11 Naphthalen-2-yl(4-(2-(3,4,5-trimethoxyphenyl)quinazolin-4-yl)piperazin-1-yl)methanone (**8k**)*

White solid; Yield 73 %; mp: 132-136 °C; ^{1}H NMR (500 MHz, DMSO-d_6): δ 8.17 (d, J = 7.8 Hz, 1H), 8.03 (s, 3H), 7.99–7.94 (m, 2H), 7.84 (t, J = 7.6 Hz, 1H), 7.76 (d, J = 8.1 Hz, 1H), 7.60 (s, 2H), 7.56 (s, 1H), 7.56–7.50 (m, 2H), 3.95-3.71 (m, 9H), 3.70-3.37 (m, 8H); ^{13}C NMR (125 MHz, DMSO-d_6): δ 169.8, 162.8, 153.4, 152.2, 149.2, 140.8, 137.7, 135.1, 133.6, 133.45, 132.7, 129.6, 128.7, 128.6, 128.1, 127.9, 127.6, 127.2, 127.1, 126.94, 126.3, 124.9, 121.3, 105.7, 60.6, 56.6; HRMS (ESI): m/z calculated for $C_{32}H_{30}IN_4O_4$ 535.2345 found 535.2380 [M+H]+.

*2.1.2.12 4-(4-(2-(3,4-dimethoxyphenyl)quinazolin-4-yl)piperazine-1-carbonyl)benzonitrile (**8l**)*

White solid; Yield 73 %; mp: 133-136 ºC; ^{1}H NMR (500 MHz, DMSO-d_6): δ 8.10 (d, J = 8.4 Hz, 1H), 8.09–8.02 (m, 2H), 7.97 (d, J = 8.2 Hz, 2H), 7.89 (d, J = 8.0 Hz, 1H), 7.82 (t, J = 7.5 Hz, 1H), 7.69 (d, J = 8.2 Hz, 2H), 7.51 (t, J = 7.5 Hz, 1H), 7.08 (d, J = 8.5 Hz, 1H), 4.05-3.91 (m, 6H), 3.85 (s, 3H), 3.80 (s, 3H), 3.59 (s, 2H); ^{13}C NMR (125 MHz, DMSO-d_6): δ 168.1, 164.4, 158.3, 152.6, 151.5, 149.0, 140.8, 133.3, 133.0, 131.0, 128.6, 128.4, 125.7, 125.5, 121.8, 118.8, 114.9, 112.6, 111.7, 111.5, 56.0, 49.3; HRMS (ESI): m/z calculated for $C_{28}H_{25}N_5O_3$ 480.2036 found 480.2079 [M+H]+.

*2.1.2.13 (4-(2-(3,4-dimethoxyphenyl)quinazolin-4-yl)piperazin-1-yl)(4-(trifluoromethyl)phenyl)methanone (**8m**)*

White solid; Yield 73 %; mp: 127-130 ºC; ^{1}H NMR (500 MHz, DMSO-d_6): δ 8.09 (d, J = 16.2 Hz, 3H), 7.88 (s, 4H), 7.73 (s, 2H), 7.51 (s, 1H), 7.09 (s, 1H), 4.11–3.74 (m, 12H), 3.61 (s, 2H); ^{13}C NMR (125 MHz, DMSO-d_6): δ 168.4, 164.4, 158.4, 152.6, 151.5, 149.0, 140.4, 133.4, 130.9, 130.1, 128.6, 128.3, 126.0, 125.7, 125.5, 123.3, 121.8, 114.9, 111.7, 111.5, 55.9, 49.4; HRMS (ESI): m/z calculated for $C_{28}H_{25}F_3N_4O_3$ 523.1957 found 523.1980 [M+H]+.

*2.1.2.14 (4-(dimethylamino)phenyl)(4-(2-(pyridin-4-yl)quinazolin-4-yl)piperazin-1-yl)methanone (**8n**)*

White solid; Yield 73 %; mp: 125-128 ºC; ^{1}H NMR (500 MHz, DMSO-d_6): δ 8.55-8.48 (m, 2H), 8.08 (d, J = 8.2 Hz, 1H), 7.91 (d, J = 7.9 Hz, 1H), 7.84 (t, J = 7.2 Hz, 1H), 7.54–7.50 (m, 3H), 7.37 (d, J = 8.7 Hz, 2H), 6.74 (d, J = 8.8 Hz, 2H), 3.91 (s, 4H), 3.81 (s, 4H), 2.97 (s, 6H); ^{13}C NMR (125 MHz, DMSO-d_6): δ 170.5, 164.5, 158.5, 152.6, 151.7, 138.4, 133.4, 130.8, 129.6, 128.8, 128.4, 125.8, 125.8, 122.3, 115.2, 111.5, 49.5, 40.3; HRMS (ESI): m/z calculated for $C_{26}H_{26}N_6O$ 439.2246 found 439.2272 [M+H]+.

*2.1.2.15 Naphthalen-2-yl(4-(2-(pyridin-4-yl)quinazolin-4-yl)piperazin-1-yl)methanone (**8o**)*

White solid; Yield 73 %; mp: 131-135 ºC; ^{1}H NMR (500 MHz, DMSO-d_6): δ 8.51 (d, J = 3.5 Hz, 2H), 8.09 (s, 2H), 8.06–7.95 (m, 3H), 7.92 (d, J = 8.2 Hz, 1H), 7.85 (t, J = 7.4 Hz, 1H), 7.65-7.58 (m, 3H), 7.55-7.49 (m, 3H), 3.90-3.75 (m, 8H); ^{13}C NMR (125 MHz, DMSO-d_6): δ 169.8, 164.5, 158.5, 152.6, 138.4, 133.6, 133.6, 133.4, 132.7, 130.8, 128.8, 128.86, 128.5, 128.4, 128.1, 127.6, 127.2, 127.1, 125.9, 125.8, 125.0, 115.2, 49.5; HRMS (ESI): m/z calculated for $C_{28}H_{23}N_5O$ 446.1981 found 446.1998 [M+H]+.

*2.1.2.16 (3,5-dimethoxyphenyl)(4-(2-(pyridin-4-yl)quinazolin-4-yl)piperazin-1-yl)methanone (**8p**)*

White solid; Yield 73 %; mp: 128-132 °C; ^{1}H NMR (500 MHz, DMSO-d_6): δ 8.56-8.49 (m, 2H), 8.07 (d, J = 8.2 Hz, 1H), 7.91 (d, J = 7.8 Hz, 1H), 7.84 (t, J = 7.2 Hz, 1H), 7.53–7.49 (m, 3H), 6.60 (s, 3H), 4.01–3.83 (m, 6H), 3.82-3.77 (m, 6H), 3.62 (s, 2H); ^{13}C NMR (125 MHz, DMSO-d_6): δ 169.3, 164.5, 160.9, 158.5, 152.6, 138.4, 133.4, 130.8, 128.8, 128.4, 125.9, 125.8, 115.2, 105.2, 101.7, 55.9, 49.4; HRMS (ESI): m/z calculated for $C_{26}H_{25}N_5O_3$ 456.2036 found 456.2072 [M+H]+.

*2.1.2.17 (4-(2-(pyridin-4-yl)quinazolin-4-yl)piperazin-1-yl)(3,4,5-trimethoxyphenyl)methanone (**8q**)*

White solid; Yield 73 %; mp: 130-134 °C; ^{1}H NMR (500 MHz, DMSO-d_6): δ 8.52 (s, 2H), 8.08 (d, J = 7.4 Hz, 1H), 7.91 (s, 1H), 7.85 (s, 1H), 7.53 (s, 3H), 6.78 (s, 2H), 3.90-3.41 (m, 17H); ^{13}C NMR (125 MHz, DMSO-d_6): δ 190.3, 169.5, 164.4, 158.5, 153.3, 152.5, 138.9, 138.4, 133.4, 131.6, 130.8, 128.8, 128.4, 125.8, 115.2, 105.1, 60.5, 56.6, 49.4; HRMS (ESI): m/z calculated for $C_{27}H_{27}N_5O_4$ 486.2141 found 486.2168 [M+H]+.

*2.1.2.18 (4-iodophenyl)(4-(2-(pyridin-4-yl)quinazolin-4-yl)piperazin-1-yl)methanone (**8r**)*

White solid; Yield 73 %; mp: 125-128 °C; ^{1}H NMR (500 MHz, DMSO-d_6): δ 8.57–8.46 (m, 2H), 8.07 (d, J = 8.2 Hz, 1H), 7.92 (d, J = 8.2 Hz, 1H), 7.86 (d, J = 8.1 Hz, 2H), 7.59–7.48 (m, 4H), 7.30 (d, J = 8.2 Hz, 2H), 3.85-3.76 (m, 8H); ^{13}C NMR (125 MHz, DMSO-d_6): δ 169.0, 164.5, 158.5, 152.6, 138.4, 137.7, 135.6, 133.4, 130.8, 129.6, 128.8, 128.4, 125.9, 125.7, 115.2, 96.9, 49.4; HRMS (ESI): m/z calculated for $C_{24}H_{20}IN_5O$ 522.0791 found 522.0819 [M+H]+.

*2.1.2.19 (4,5-dimethoxy-2-nitrophenyl)(4-(2-(pyridin-4-yl)quinazolin-4-yl)piperazin-1-yl)methanone (**8s**)*

White solid; Yield 73 %; mp: 127-130 °C; ^{1}H NMR (500 MHz, DMSO-d_6): δ 8.51 (s, 2H), 8.09 (s, 1H), 7.92 (s, 1H), 7.86 (s, 1H), 7.76 (s, 1H), 7.52 (s, 3H), 7.16 (s, 1H), 4.10-3.95 (m, 9H), 3.82 (s, 3H), 3.49 (s, 2H); ^{13}C NMR (125 MHz, DMSO-d_6): δ 169.4, 164.4, 158.5, 152.5, 138.4, 135.3, 133.4, 131.5, 130.8, 128.8, 128.8, 128.6, 128.5, 128.4, 125.8, 125.8, 115.2, 49.4, 45.2, 41.6; HRMS (ESI): m/z calculated for $C_{26}H_{24}N_6O_5$ 501.1886 found 501.1906 [M+H]+.

*2.1.2.20 (5-bromo-2-chlorophenyl)(4-(2-(pyridin-4-yl)quinazolin-4-yl)piperazin-1-yl)methanone (**8t**)*

White solid; Yield 73 %; mp: 125-128 °C; ^{1}H NMR (500 MHz, DMSO-d_6): δ 8.56–8.46 (m, 2H), 8.08 (d, J = 8.1 Hz, 1H), 7.92 (d, J = 7.7 Hz, 1H), 7.85 (t, J = 7.1 Hz, 1H), 7.75 (d, J = 8.6 Hz, 1H), 7.62 (d, J = 2.5 Hz, 1H), 7.57–7.46 (m, 5H), 3.99-3.55 (m, 6H), 3.46 (d, J = 4.2 Hz, 2H); ^{13}C NMR

(125 MHz, DMSO-d_6): δ 165.7, 164.6, 158.5, 152.5, 140.0, 138.3, 134.8, 133.5, 133.4, 131.0, 130.8, 128.8, 128.4, 128.3, 126.0, 125.7, 117.4, 115.2, 49.3, 46.4; HRMS (ESI): m/z calculated for $C_{24}H_{19}BrClN_5O$ 508.0540 found 510.0532 [M+2]+.

*2.1.2.21 2-(4-chlorophenyl)-1-(4-(2-(pyridin-4-yl)quinazolin-4-yl)piperazin-1-yl)ethan-1-one (**8u**)*

White solid; Yield 73 %; mp: 132-136 ºC; ^{1}H NMR (500 MHz, DMSO-d_6): δ 8.57–8.46 (m, 2H) 8.06 (d, J = 8.2 Hz, 1H), 7.91 (d, J = 7.9 Hz, 1H), 7.84 (t, J = 7.2 Hz, 1H), 7.54–7.51 (m, 3H), 7.38 (d, J = 8.4 Hz, 2H), 7.29 (d, J = 8.4 Hz, 2H), 3.82 (d, J = 8.9 Hz, 8H), 3.77 (d, J = 4.9 Hz, 2H); ^{13}C NMR (125 MHz, DMSO-d_6): δ 168.2, 164.2, 155.8, 152.4, 144.5, 140.8, 133.6, 133.0, 130.6, 129.0, 128.7, 128.4, 128.3, 125.9, 125.7, 118.8, 115.0, 112.7, 49.5; HRMS (ESI): m/z calculated for $C_{25}H_{22}ClN_5O$ 444.1591 found 444.1623 [M+H]+.

*2.1.2.22 (5-bromo-2-chlorophenyl)(4-(2-(thiophen-2-yl)quinazolin-4-yl)piperazin-1-yl)methanone (**8v**)*

White solid; Yield 73 %; mp: 130-133 ºC; ^{1}H NMR (500 MHz, DMSO-d_6): δ 8.03 (d, J = 8.3 Hz, 1H), 8.00–7.97 (m, 1H), 7.84–7.80 (m, 2H), 7.74 (d, J = 8.6 Hz, 1H), 7.75-7.71 (m, 1H), 7.61 (d, J = 2.6 Hz, 1H), 7.53–7.45 (m, 2H), 7.22–7.18 (m, 1H), 3.98–3.80 (m, 6H), 3.43 (d, J = 3.3 Hz, 2H); ^{13}C NMR (125 MHz, DMSO-d_6): δ 165.9, 162.5, 150.7, 141.9, 139.6, 136.2, 136.0, 135.9, 134.7, 133.5, 133.1, 131.1, 129.9, 128.3, 127.6, 127.1, 114.6, 112.3, 112.1, 45.4; HRMS (ESI): m/z calculated for $C_{23}H_{18}BrClN_4OS$ 513.0151 found 515.0139 [M+H]+.

*2.1.2.23 (4,5-dimethoxy-2-nitrophenyl)(4-(2-(thiophen-2-yl)quinazolin-4-yl)piperazin-1-yl)methanone (**8w**)*

White solid; Yield 73 %; mp: 129-133 ºC; ^{1}H NMR (500 MHz, DMSO-d_6): δ 8.04 (d, J = 8.3 Hz, 1H), 8.00–7.96 (m, 1H), 7.85–7.80 (m, 2H), 7.75 (s, 1H), 7.75-7.71 (m, 1H),7.54-7.50 (m, 1H), 7.24-7.19 (m, 1H), 7.15 (s, 1H), 4.02–3.87 (m, 10H), 3.78 (s, 2H), 3.47 (s, 2H); ^{13}C NMR (125 MHz, DMSO-d_6): δ 166.2, 164.4, 155.7, 154.5, 152.4, 149.1, 144.4, 138.0, 133.6, 130.6, 129.0, 128.7, 128.3, 127.3, 125.9, 125.7, 115.1, 110.2, 107.8, 57.2, 56.7, 49.2, 46.3; HRMS (ESI): m/z calculated for $C_{25}H_{23}N_5O_5S$ 506.1498 found 506.1536 [M+H]+.

*2.1.2.24 Naphthalen-2-yl(4-(2-(thiophen-2-yl)quinazolin-4-yl)piperazin-1-yl)methanone (**8x**)*

White solid; Yield 73 %; mp: 128-132 ºC; ^{1}H NMR (500 MHz, DMSO-d_6): δ 8.08 (s, 1H), 8.06–7.96 (m, 5H), 7.84–7.79 (m, 2H), 7.74–7.70 (m, 1H), 7.65-7.57 (m, 3H), 7.53–7.47 (m, 1H), 7.22–7.17 (m, 1H), 3.90-3.71 (m, 8H); ^{13}C NMR (125 MHz, DMSO-d_6): δ 169.8, 164.2, 155.8, 152.4,

144.5, 133.6, 133.9, 132.7, 130.5, 129.0, 128.8, 128.6, 128.5, 128.3, 128.1, 127.6, 127.2, 127.1, 125.9, 125.6, 125.0, 115.0, 49.4; HRMS (ESI): m/z calculated for $C_{27}H_{22}N_4OS$ 451.1593 found 451.1624 [M+H]+.

*2.1.2.25 4-(4-(2-(furan-2-yl)quinazolin-4-yl)piperazine-1-carbonyl)benzonitrile (**8y**)*

White solid; Yield 73 %; mp: 128-132 ^oC; ^1H NMR (500 MHz, DMSO-d_6): δ 8.15–7.90 (m, 4H), 7.83 (s, 2H), 7.77-7.69 (m, 3H), 7.51 (s, 1H), 7.20 (s, 1H), 4.16–3.73 (m, 6H), 3.57 (s, 2H); ^{13}C NMR (125 MHz, DMSO-d_6): δ 168.1, 164.2, 155.7, 152.4, 144.4, 140.7, 133.6, 133.0, 130.5, 129.0, 128.6, 128.4, 128.3, 125.8, 125.6, 118.8, 115.0, 112.6, 49.2; HRMS (ESI): m/z calculated for $C_{24}H_{19}N_5O_2$ 410.1617 found 410.1649 [M+H]+.

*2.1.2.26 (4,5-dimethoxy-2-nitrophenyl)(4-(2-(furan-2-yl)quinazolin-4-yl)piperazin-1-yl)methanone (**8z**)*

White solid; Yield 73 %; mp: 132-136 ^oC; ^1H NMR (500 MHz, DMSO-d_6): δ 8.04 (d, J = 8.3 Hz, 1H), 8.00–7.96 (m, 1H), 7.85–7.80 (m, 2H), 7.75 (s, 1H), 7.73-7.69 (m, 1H), 7.55-7.51 (m, 1H), 7.20-7.17 (m, 1H), 7.15 (s, 1H), 4.02–3.87 (m, 10H), 3.78 (s, 2H), 3.47 (s, 2H); ^{13}C NMR (125 MHz, DMSO-d_6): δ 166.2, 164.4, 155.7, 154.5, 152.4, 149.1, 144.4, 138.0, 133.6, 130.6, 129.0, 128.7, 128.3, 127.3, 125.9, 125.7, 115.1, 110.2, 107.8, 57.2, 56.7, 49.2, 46.3; HRMS (ESI): m/z calculated for $C_{25}H_{23}N_5O_6$ 490.1727 found 490.1752 [M+H]+.

2.2 Antibiotic susceptibility testing against ESKAP pathogen panel

Antibiotic susceptibility testing was carried out on the newly synthesized compounds by determining the Minimum Inhibitory Concentration (MIC) with reference to the standard CLSI guidelines [17, 18]. MIC is defined as the minimum concentration of compound at which visible bacterial growth is inhibited. Bacterial cultures were grown in Mueller-Hinton cation supplemented broth (CA-MHB). Optical density (OD_{600}) of the cultures was measured, followed by dilution for ~10^6 cfu/mL. This inoculum was added into a series of test wells in a microtitre plate that contained various concentrations of compound under test ranging from 64-0.03 μg/mL. Controls i.e., cells alone and media alone (without compound+cells) and levofloxacin used as a reference standard. Plates were incubated at 37 °C for 16-18 h followed by observations of MIC values by the absence or presence of visible growth. For each compound, MIC determinations were performed independently thrice using duplicate samples each time.

2.3 Antibiotic susceptibility testing against pathogenic mycobacteria

Antimycobacterial susceptibility testing was carried out on the newly synthesized compounds by using broth micro dilution assay [19]. 1g/100 mL stock solutions of test and control compounds were prepared in DMSO and stored in −20 °C. Mycobacterial cultures were inoculated in Middlebrook 7H9 enriched (Difco, Becton, NJ, USA) media supplemented with 10% ADC-Tween-80 (Bovine Serum Albumin, Dextrose, 0.2% glycerol and 0.05% Tween-80) and OD_{600} of the cultures was measured, followed by dilution to achieve ~10^6 cfu/mL [20]. The newly synthesized compounds were tested from 0.0064–0.00005 g/100 mL in two-fold serial diluted fashion with 2.5 μL of each concentration added per well of a 96-well round bottom microtitre plate. Later, 97.5 μL of bacterial suspension was added to each well containing the test compound along with appropriate controls. Presto blue (Thermo Fisher, USA) resazurin-based dye was used for the visualized identification of active compounds. MIC of active compound was determined as lowest concentration of compound that inhibited visible growth after incubation period. For each compound, MIC determinations were replicated thrice using duplicate samples. The MIC plates were incubated at 37 °C for 7 days for Mtb.

3.0 Copies of spectra

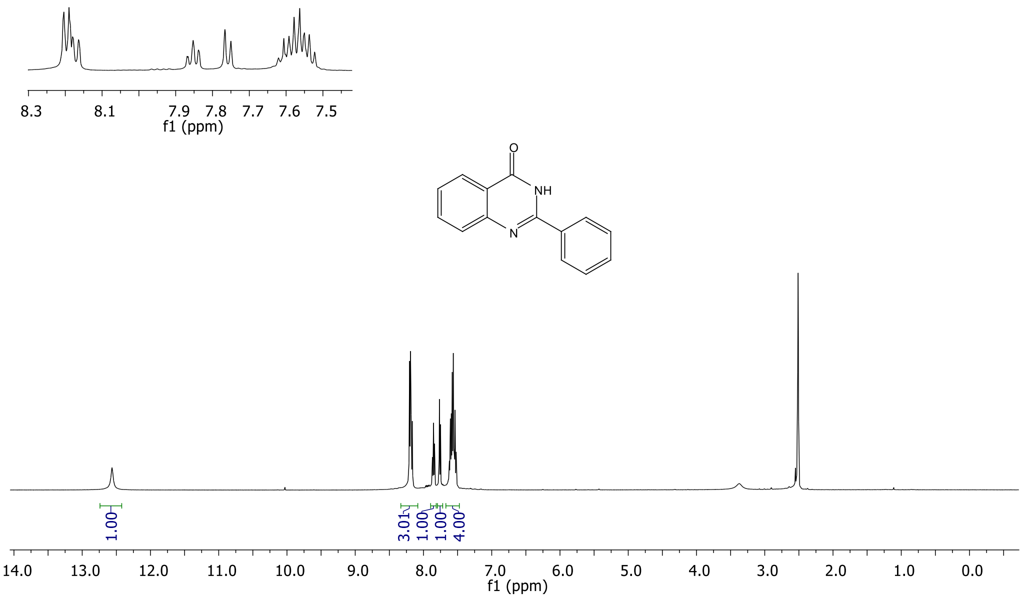

Compound **3a**: ^{1}H NMR (500 MHz, DMSO-d_6).

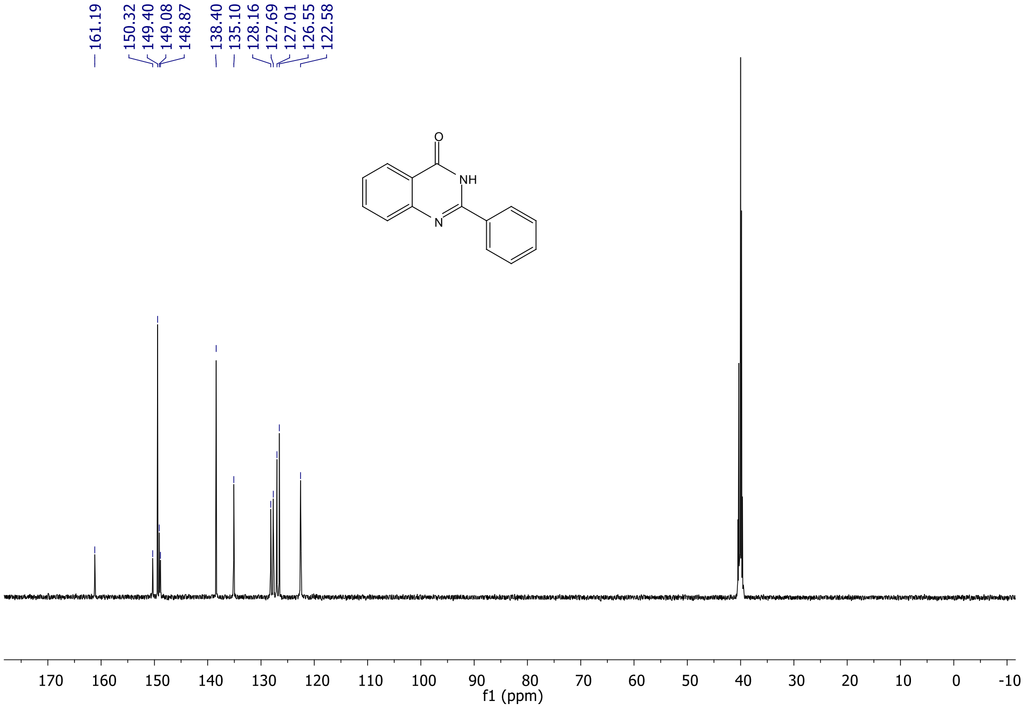

Compound **3a**: ^{13}C NMR (125 MHz, DMSO-d_6).

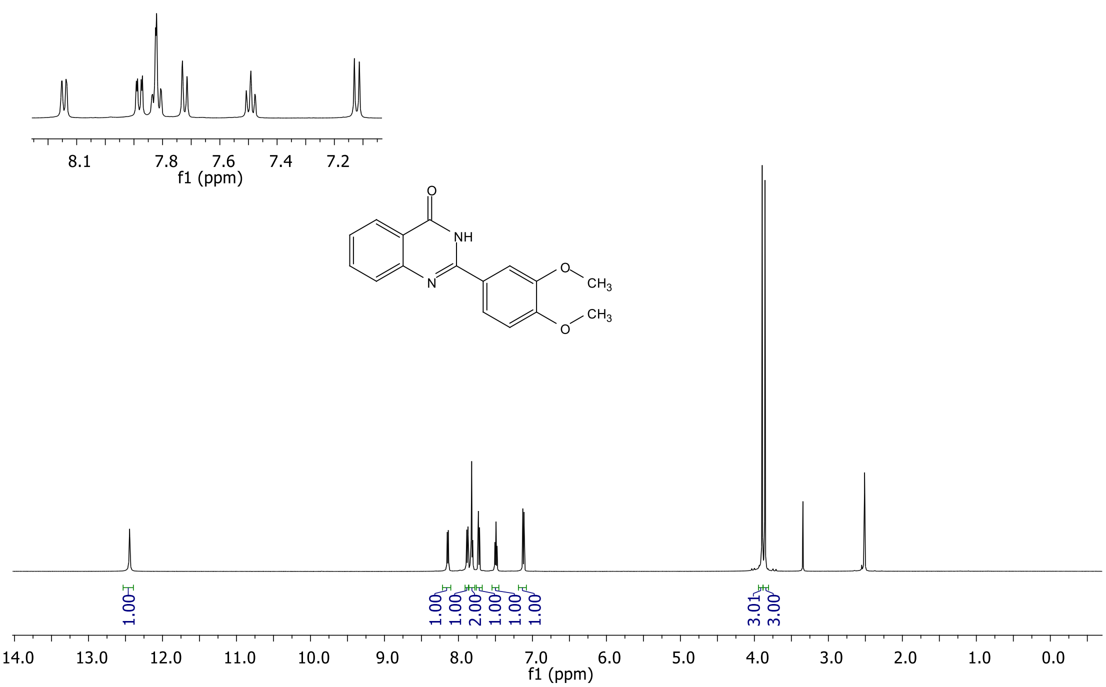

Compound **3b**: ^{1}H NMR (500 MHz, DMSO-d_6).

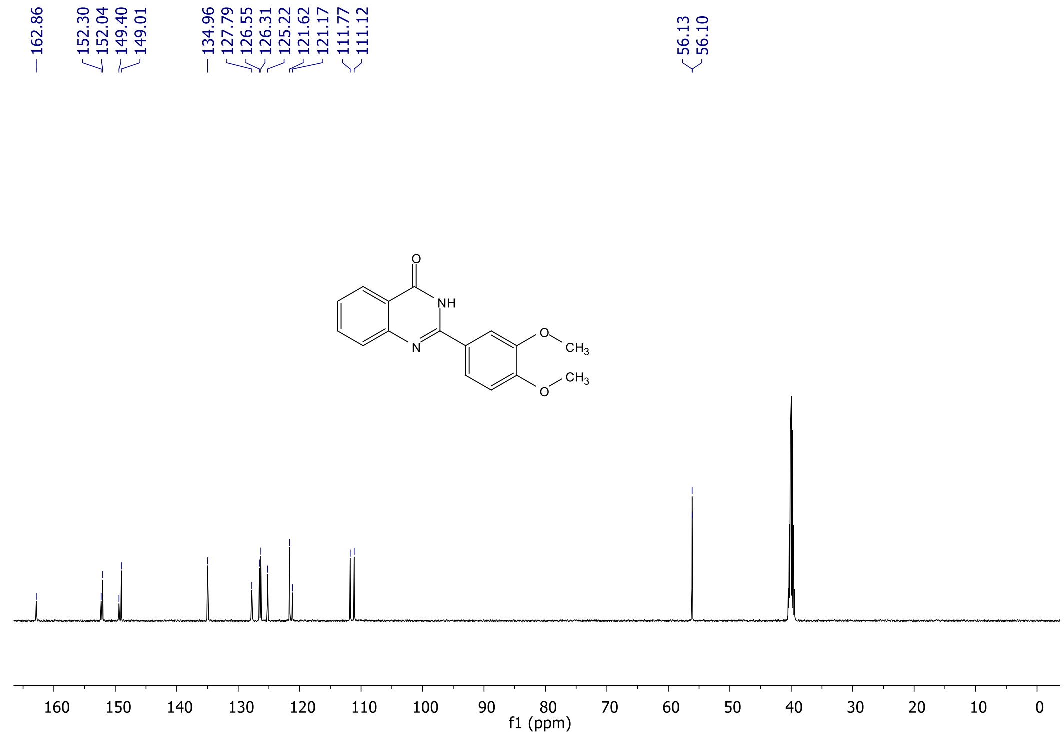

Compound **3b**: ^{13}C NMR (125 MHz, DMSO-d_6).

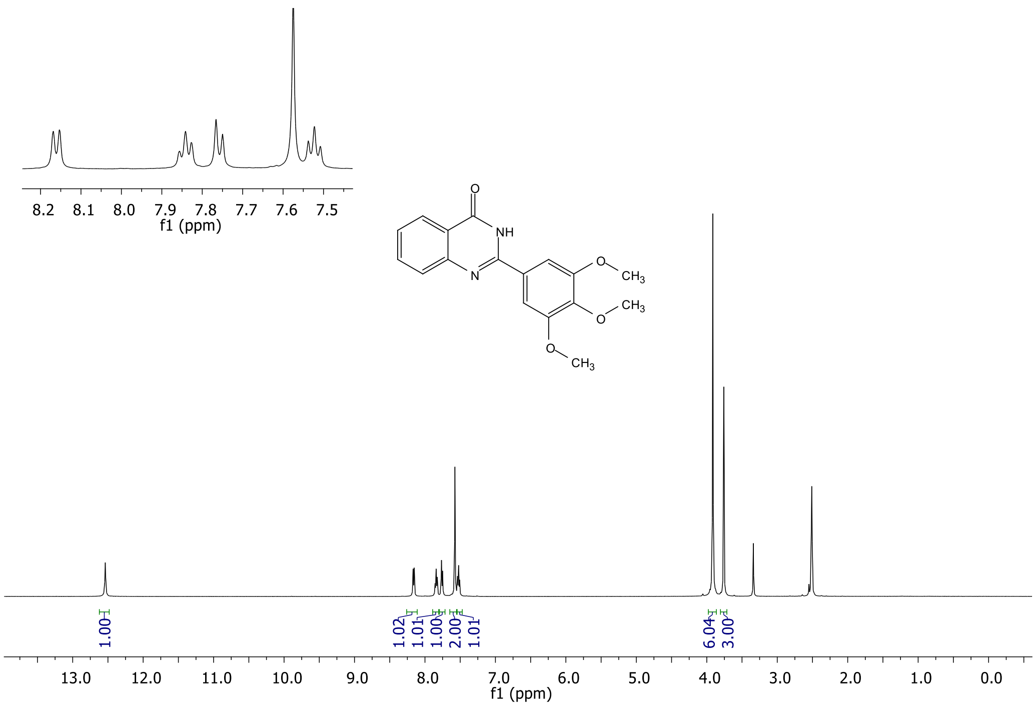

Compound **3c**: ^{1}H NMR (500 MHz, DMSO-d_6).

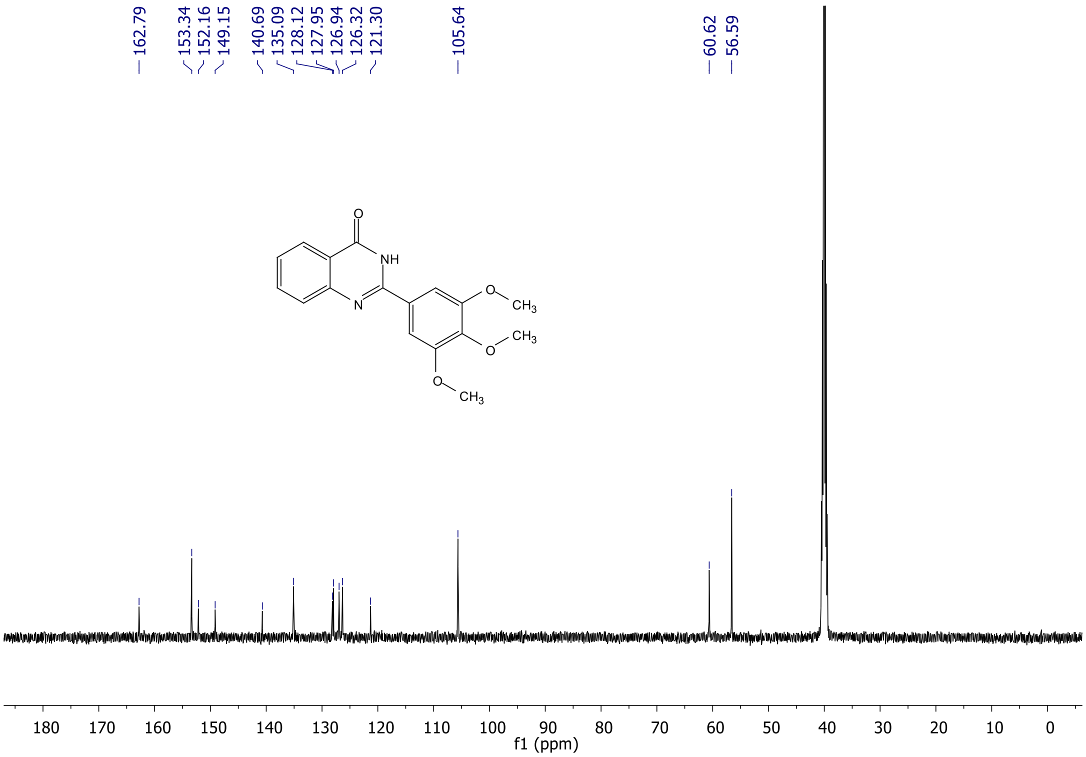

Compound **3c**: ^{13}C NMR (125 MHz, DMSO-d_6).

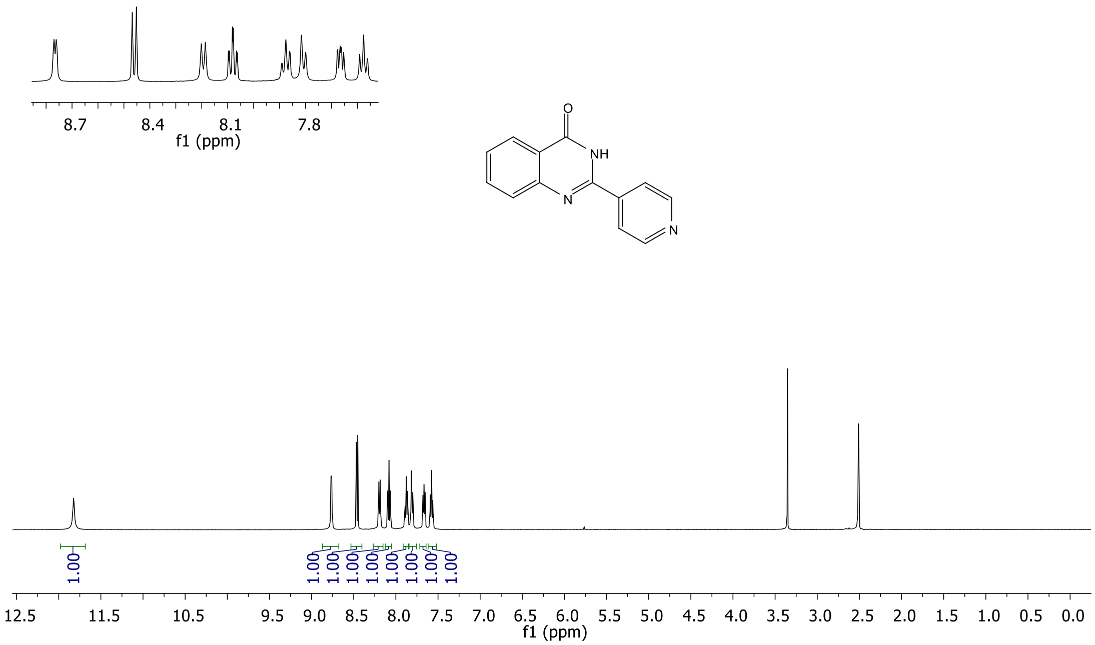

Compound **3d**: ^{1}H NMR (500 MHz, DMSO-d_6).

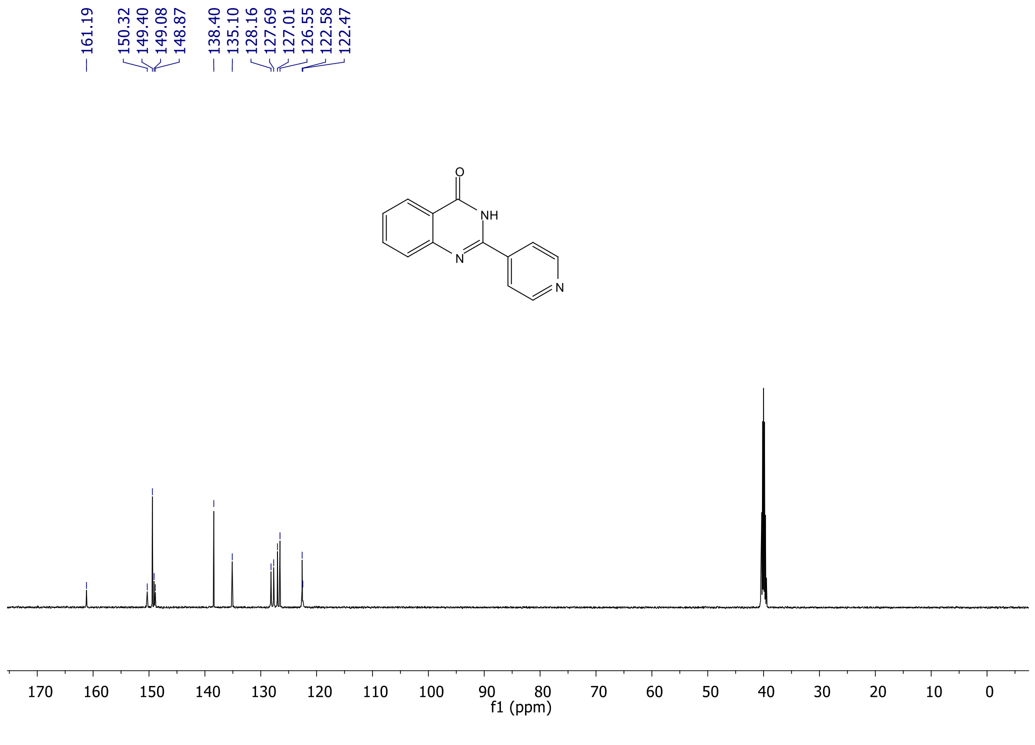

Compound **3d**: ^{13}C NMR (125 MHz, DMSO-d_6)

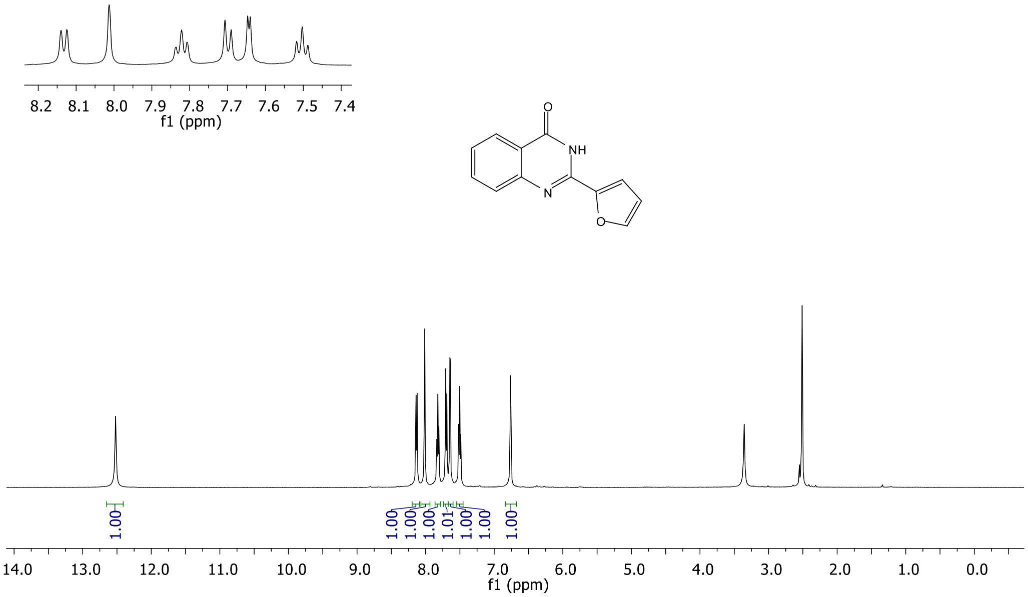

Compound **3e**: ^{1}H NMR (500 MHz, DMSO-d_6).

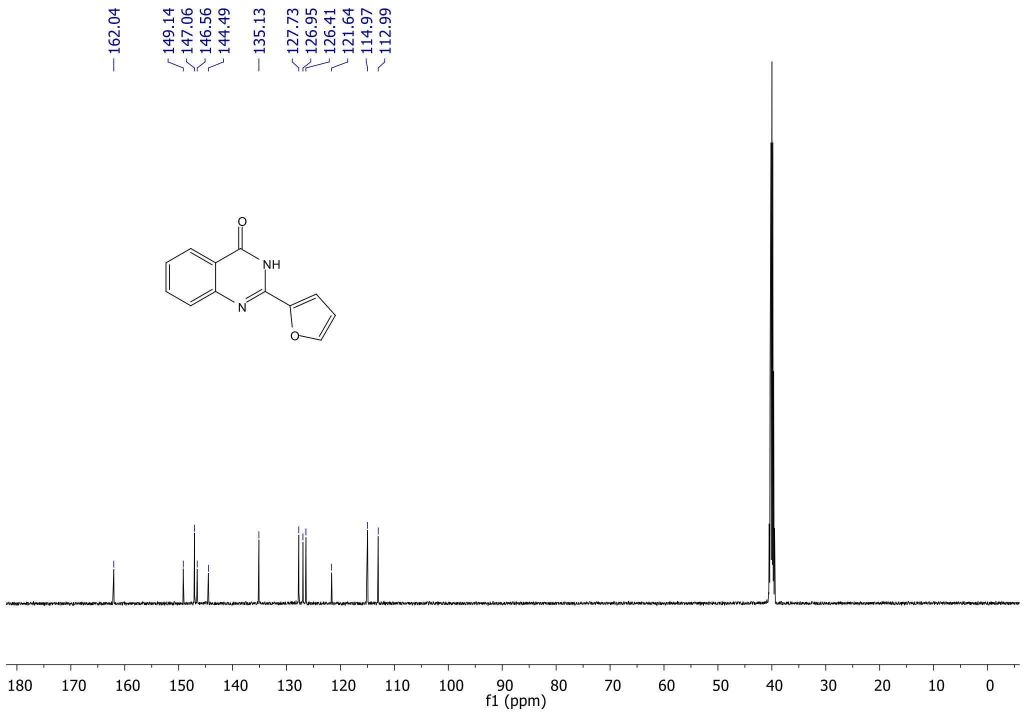

Compound **3e**: ^{13}C NMR (125 MHz, DMSO-d_6)

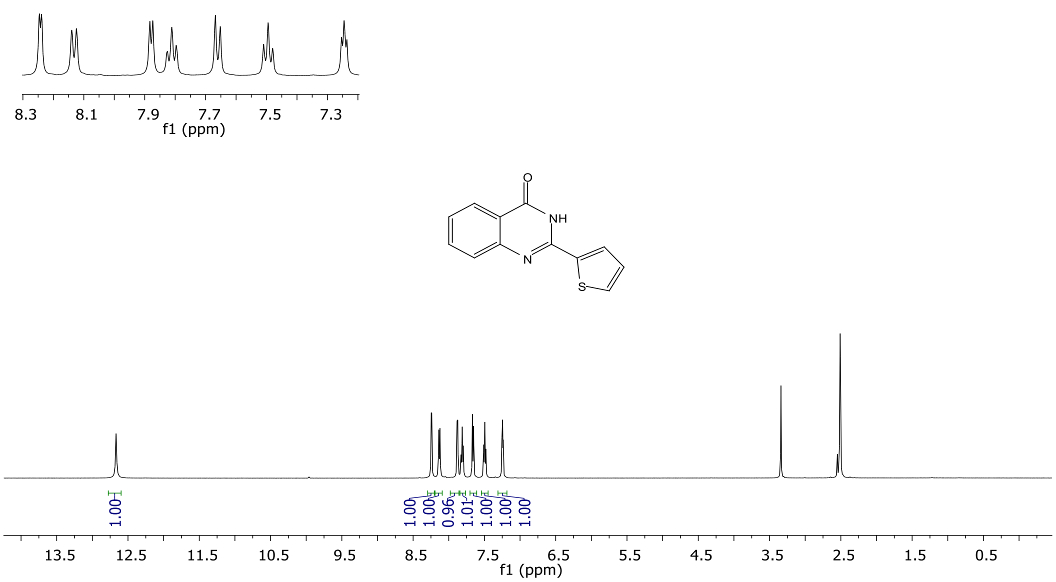

Compound **3f**: ^{1}H NMR (500 MHz, DMSO-d_6).

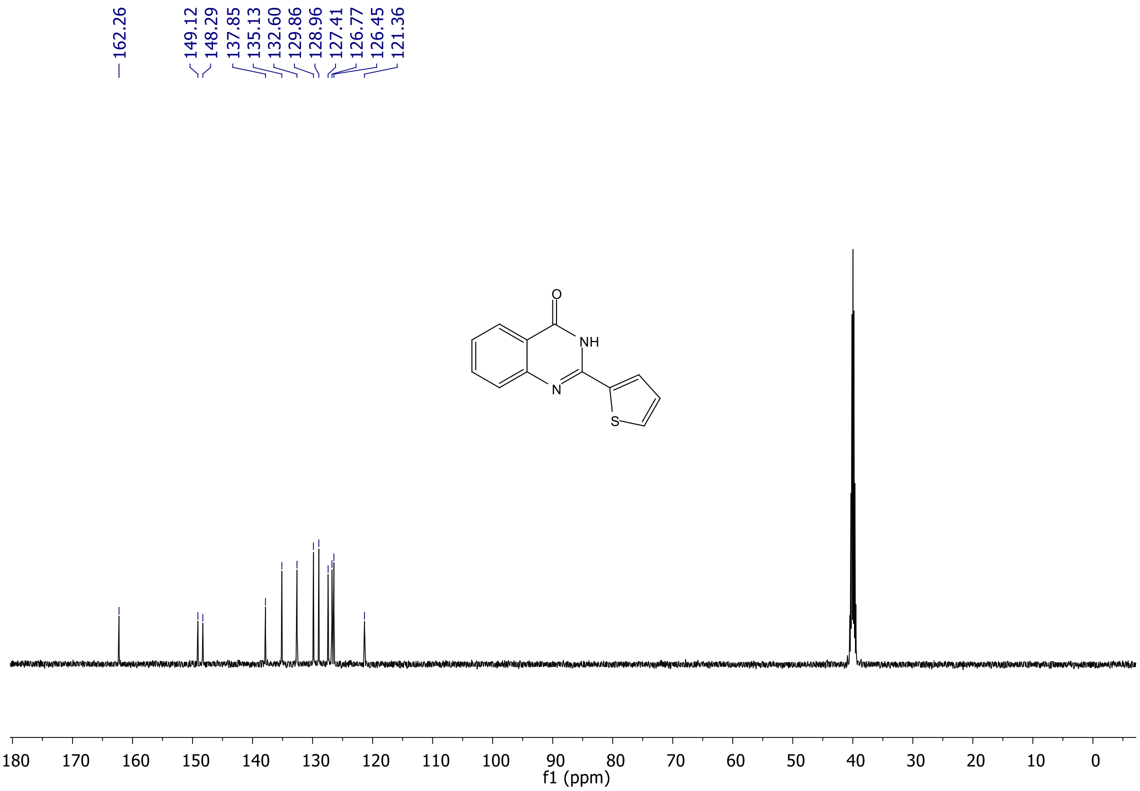

Compound **3f**: ^{13}C NMR (125 MHz, DMSO-d_6).

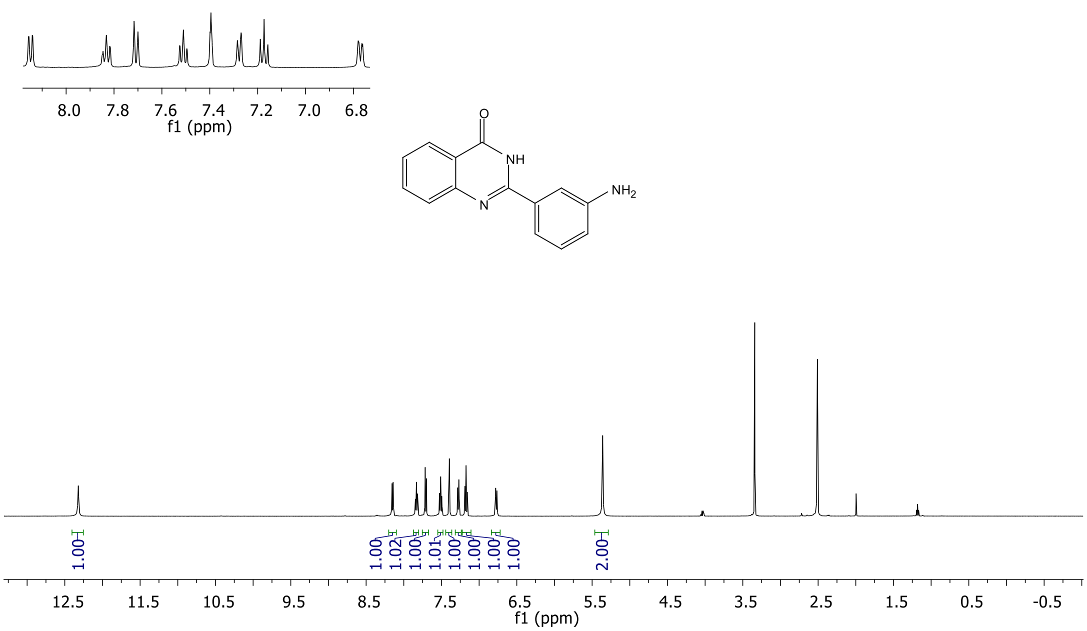

Compound **3g**: ^{1}H NMR (500 MHz, DMSO-d_6).

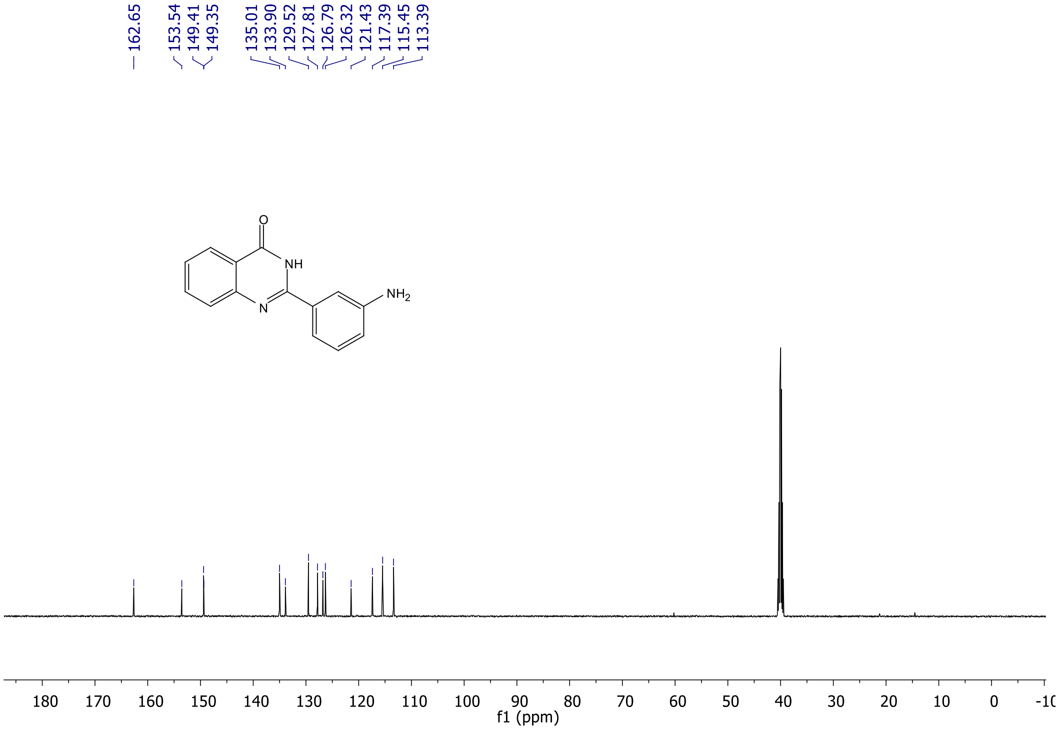

Compound **3g**: ^{13}C NMR (125 MHz, DMSO-d_6).

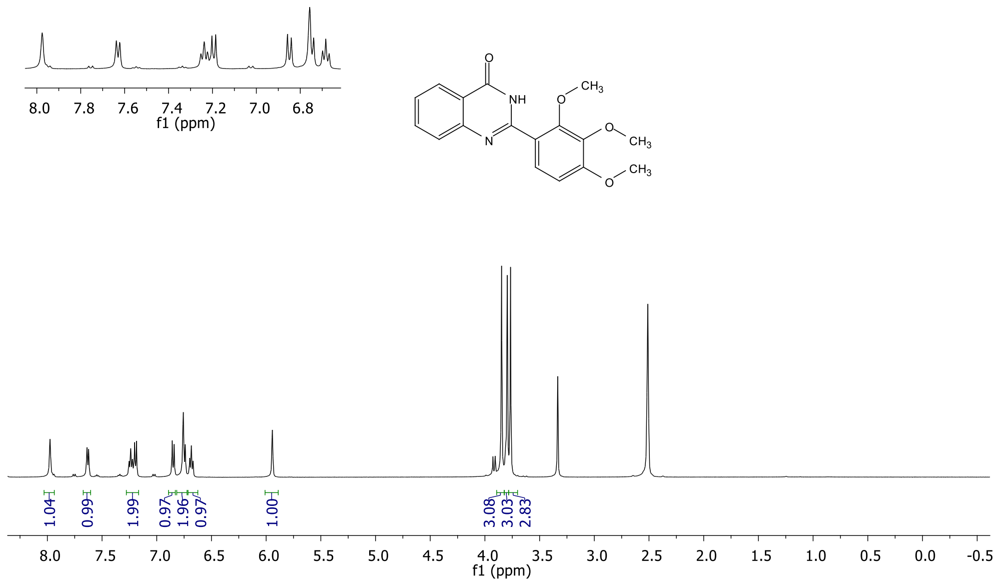

Compound **3h**: ^{1}H NMR (500 MHz, DMSO-d_6).

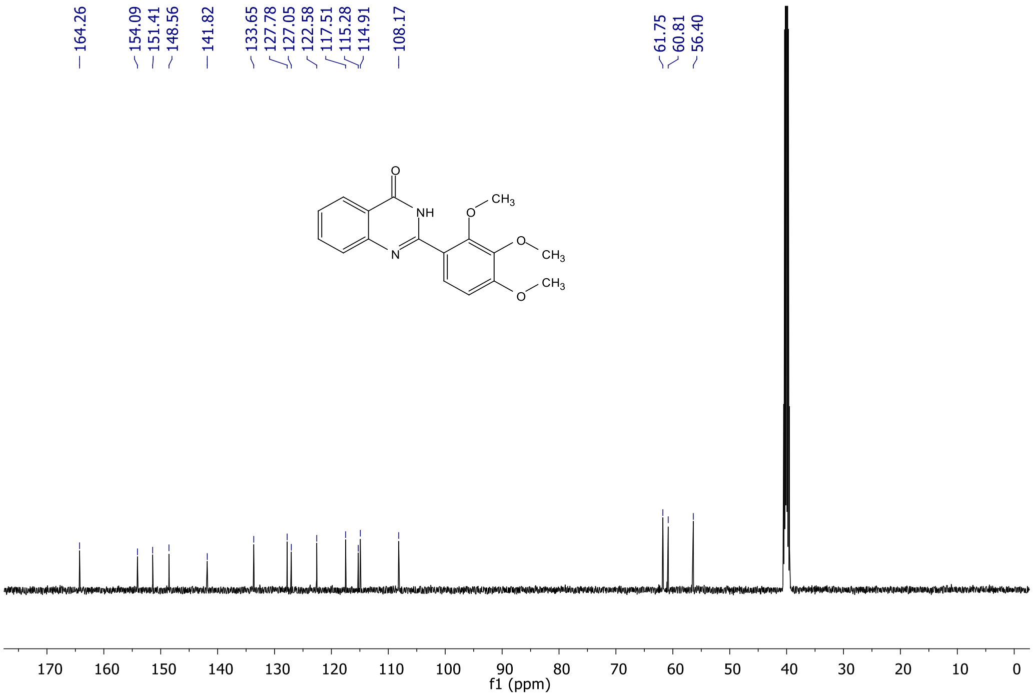

Compound **3h**: ^{13}C NMR (125 MHz, DMSO-d_6).

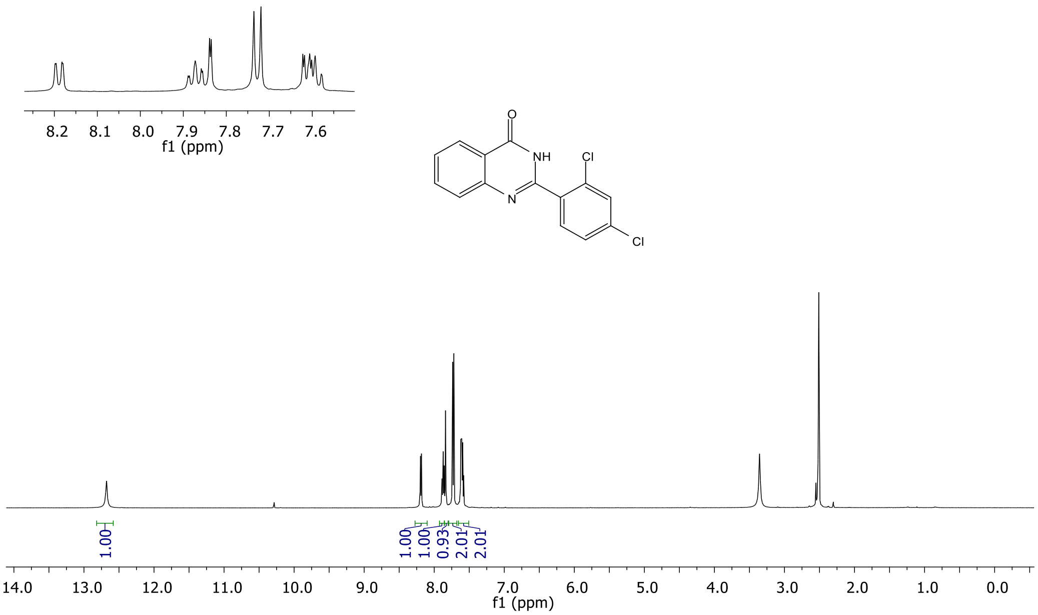

Compound **3i**: ^{1}H NMR (500 MHz, DMSO-d_6).

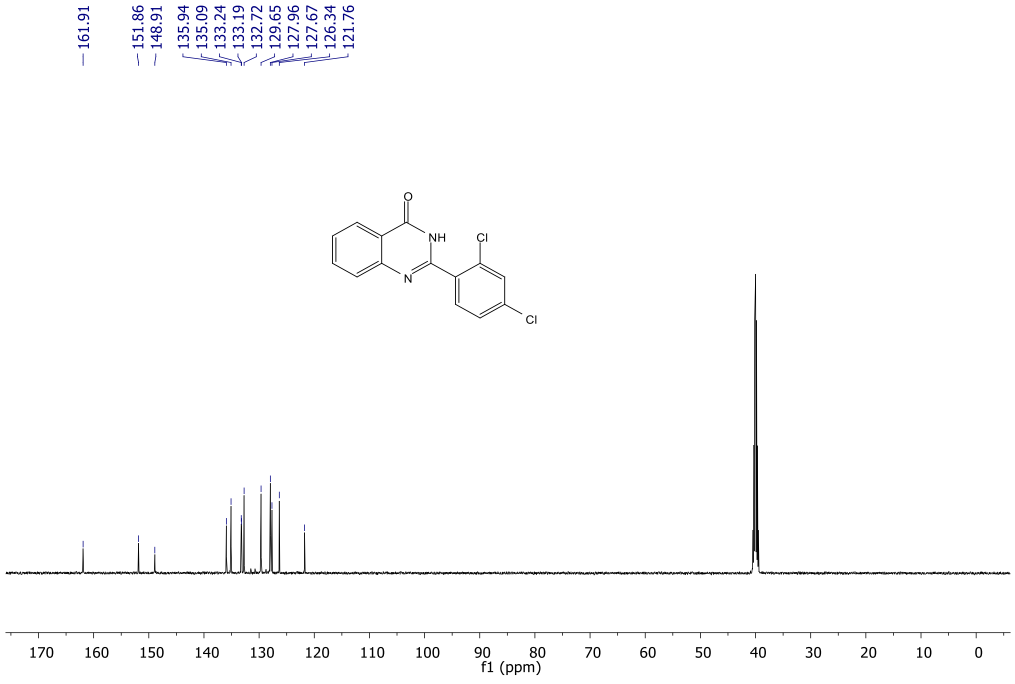

Compound **3i**: ^{13}C NMR (125 MHz, DMSO-d_6).

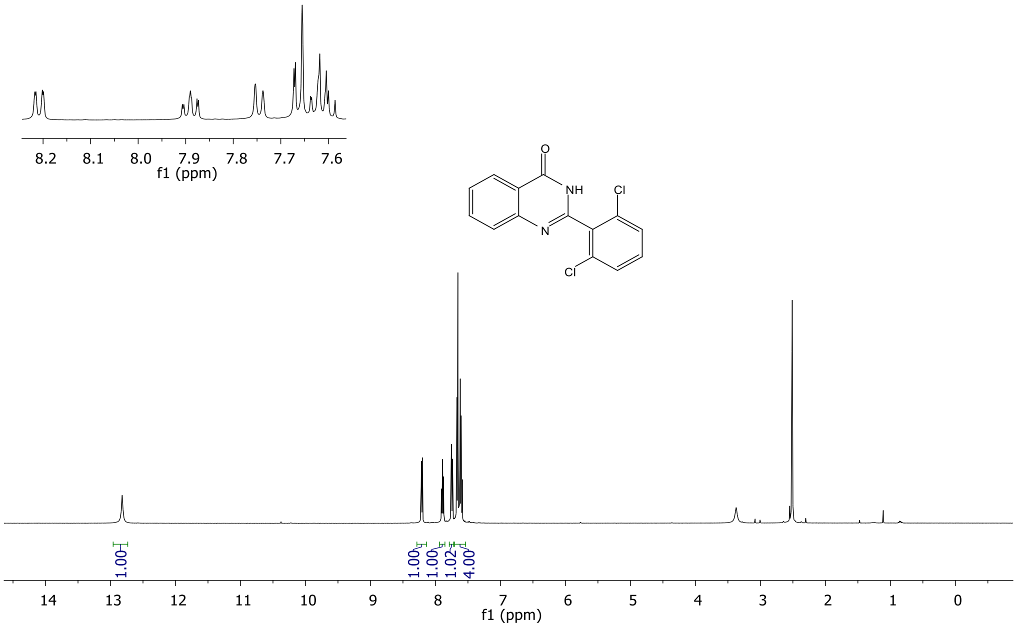

Compound **3j**: ^{1}H NMR (500 MHz, DMSO-d_6).

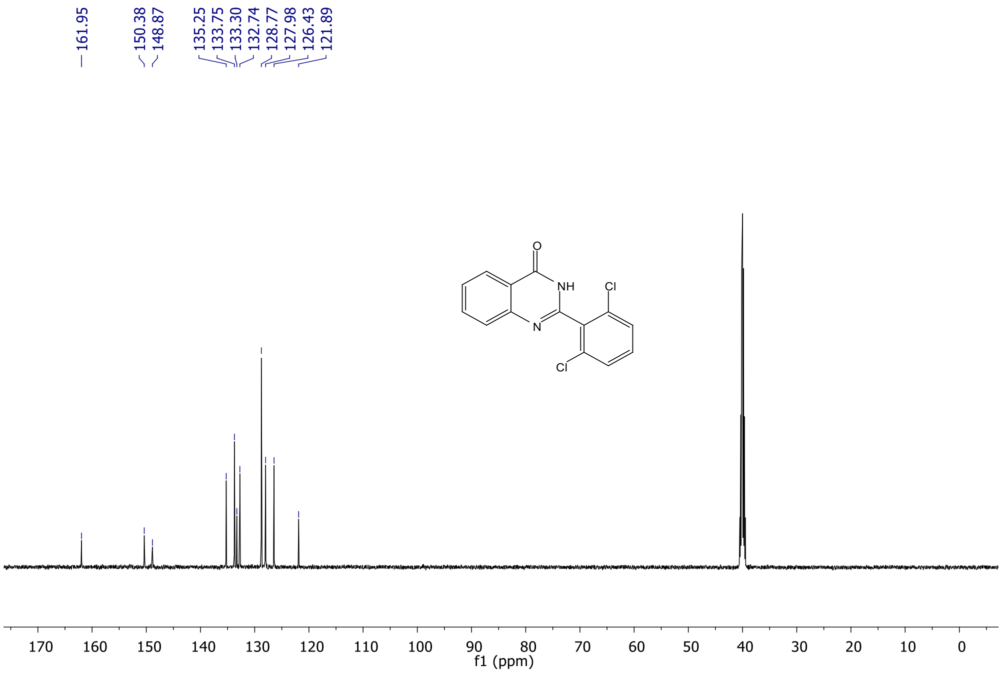

Compound **3j**: ^{13}C NMR (125 MHz, DMSO-d_6).

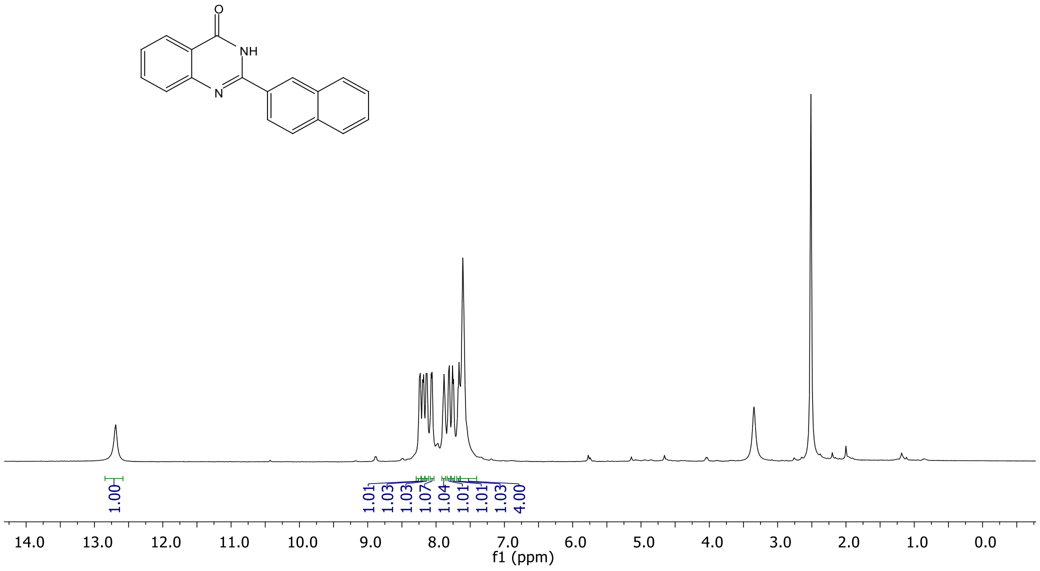

Compound **3k**: ^{1}H NMR (500 MHz, DMSO-d_6).

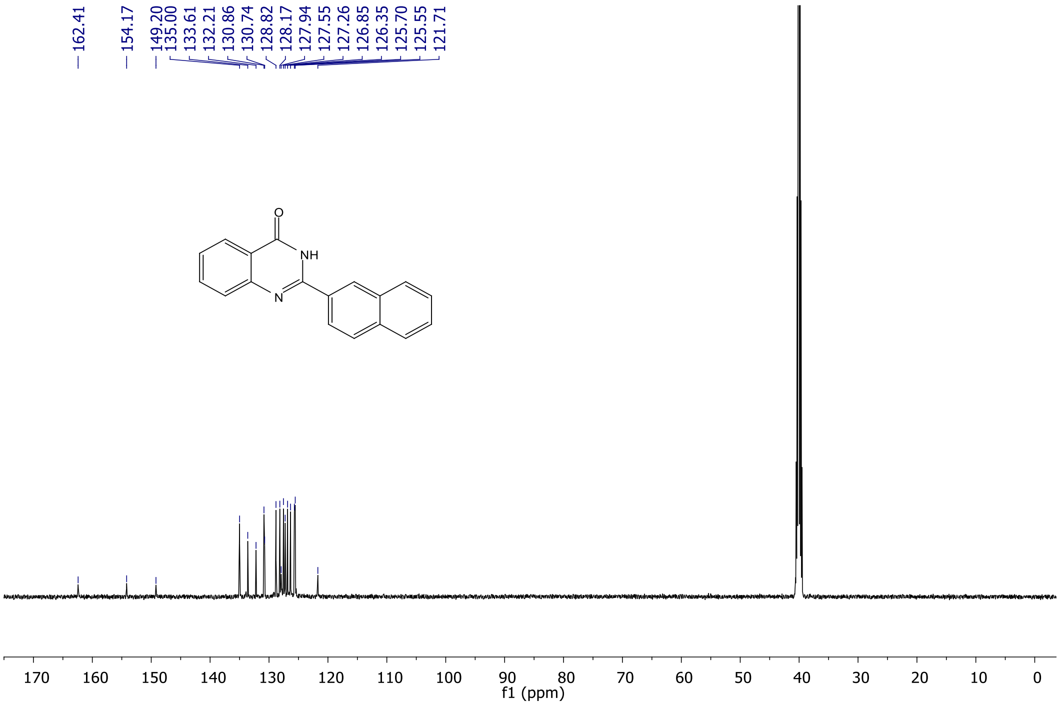

Compound **3k**: ^{13}C NMR (125 MHz, DMSO-d_6).

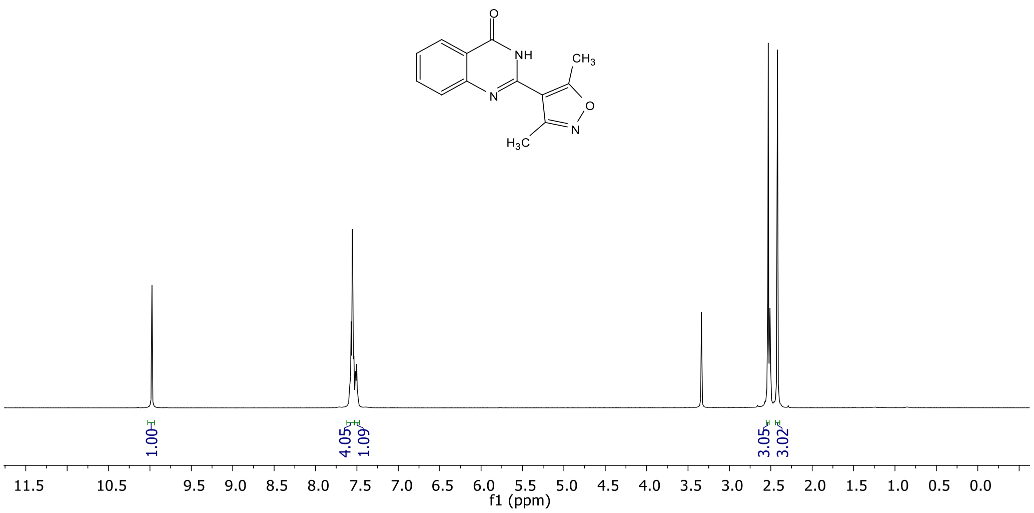

Compound **3m**: ^{1}H NMR (500 MHz, DMSO-d_6).

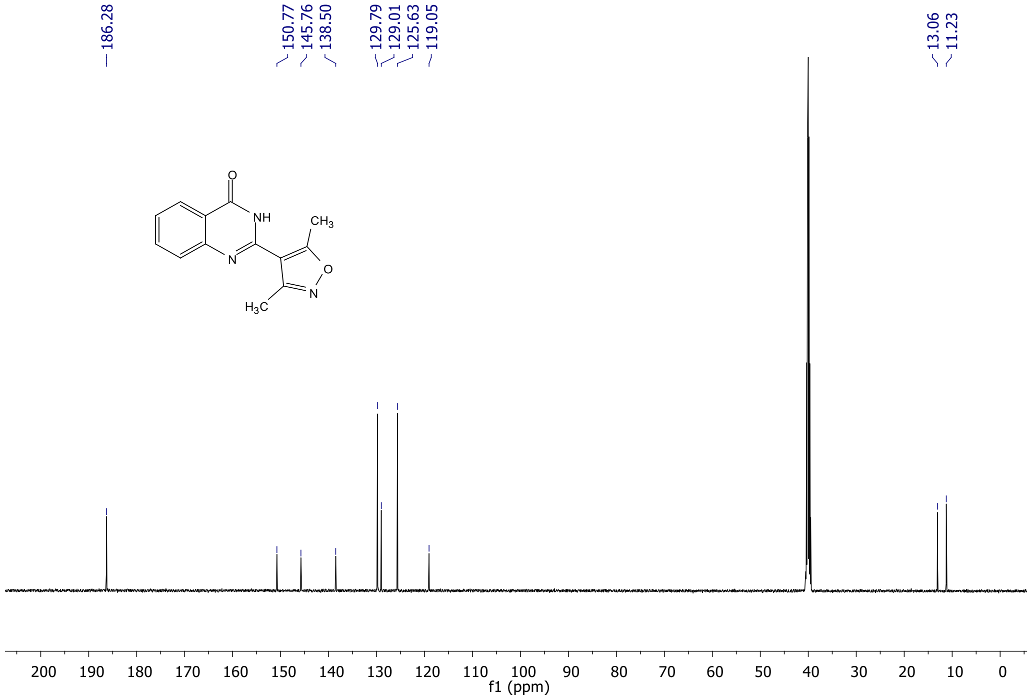

Compound **3m**: ^{13}C NMR (125 MHz, DMSO-d_6).

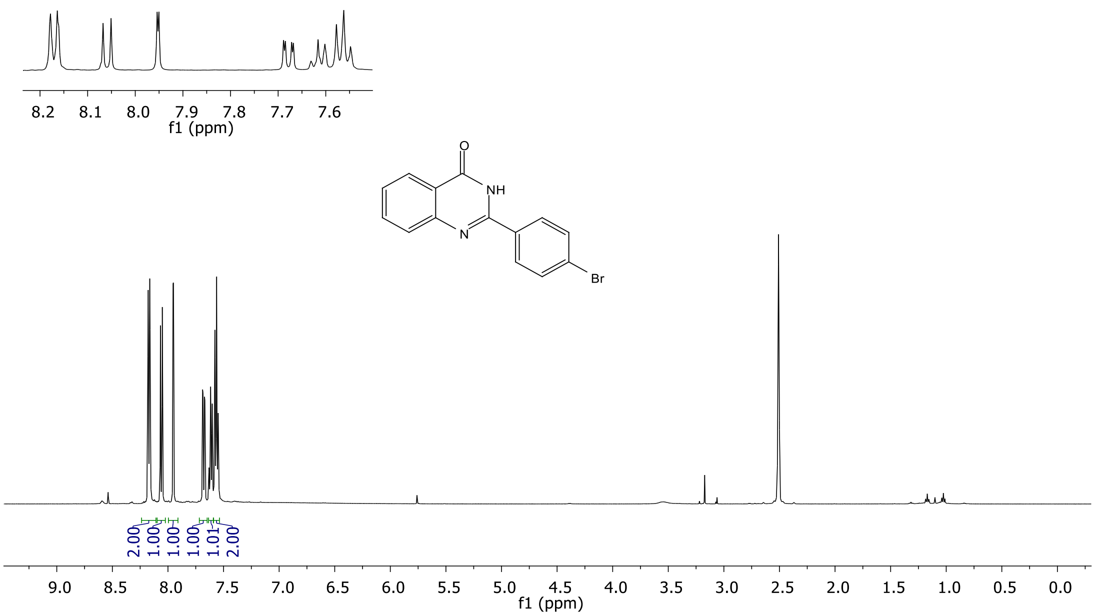

Compound **3n**: ^{1}H NMR (500 MHz, DMSO-d_6).

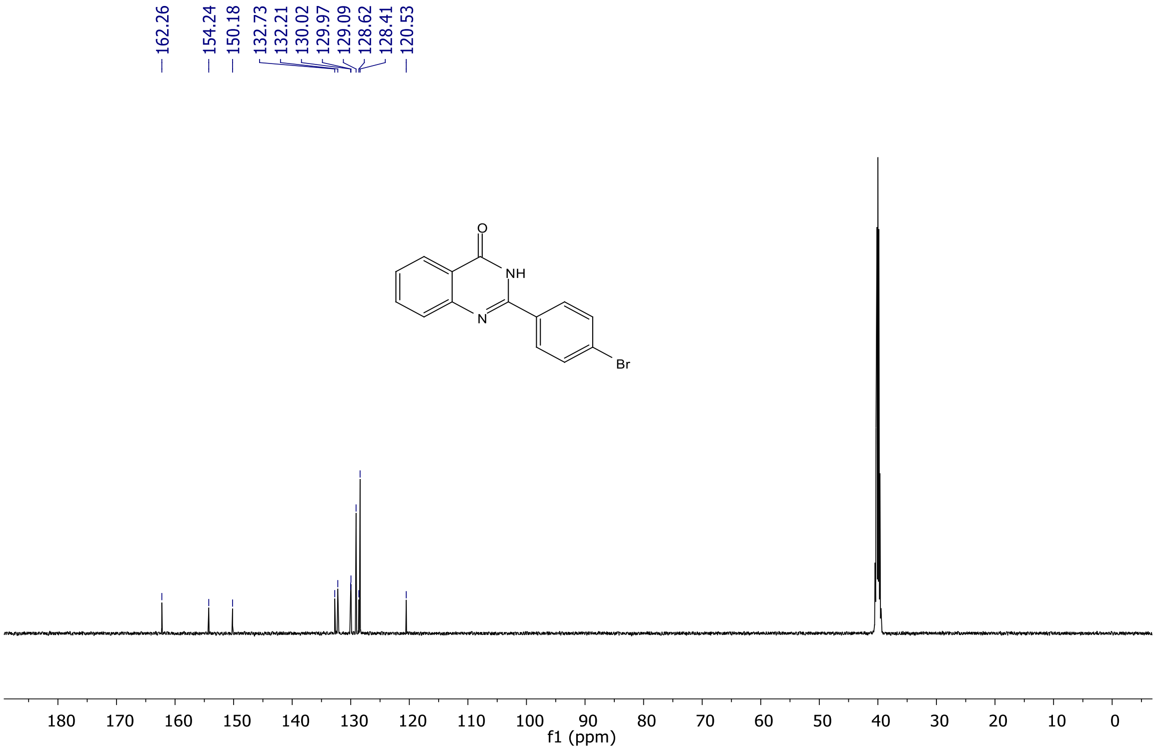

Compound **3n**: ^{13}C NMR (125 MHz, DMSO-d_6).

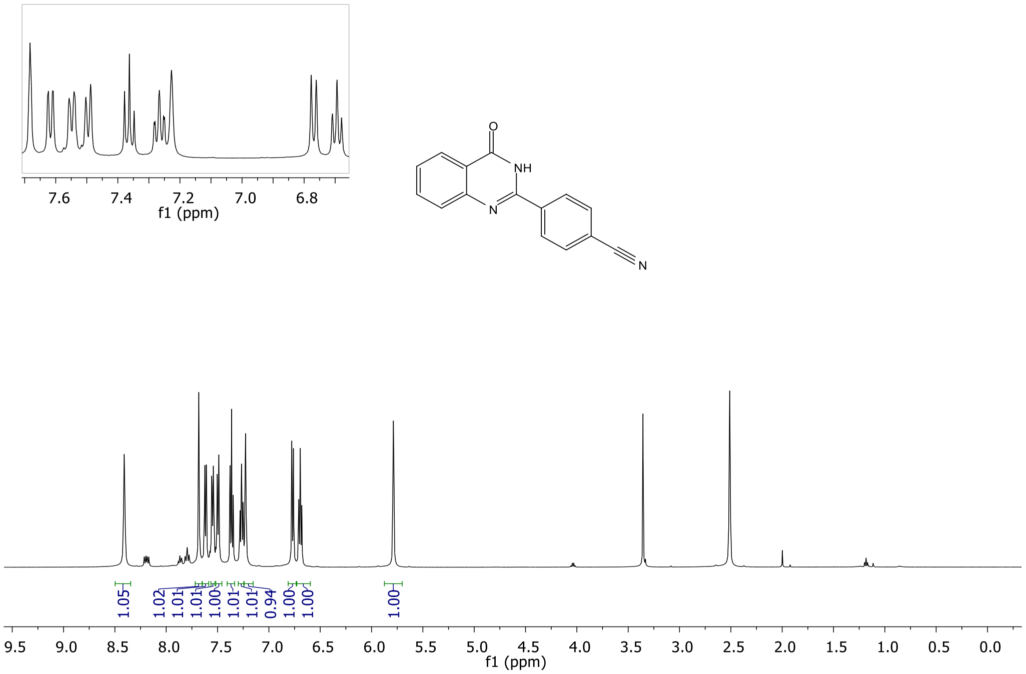

Compound **3o**: ^{1}H NMR (500 MHz, DMSO-d_6).

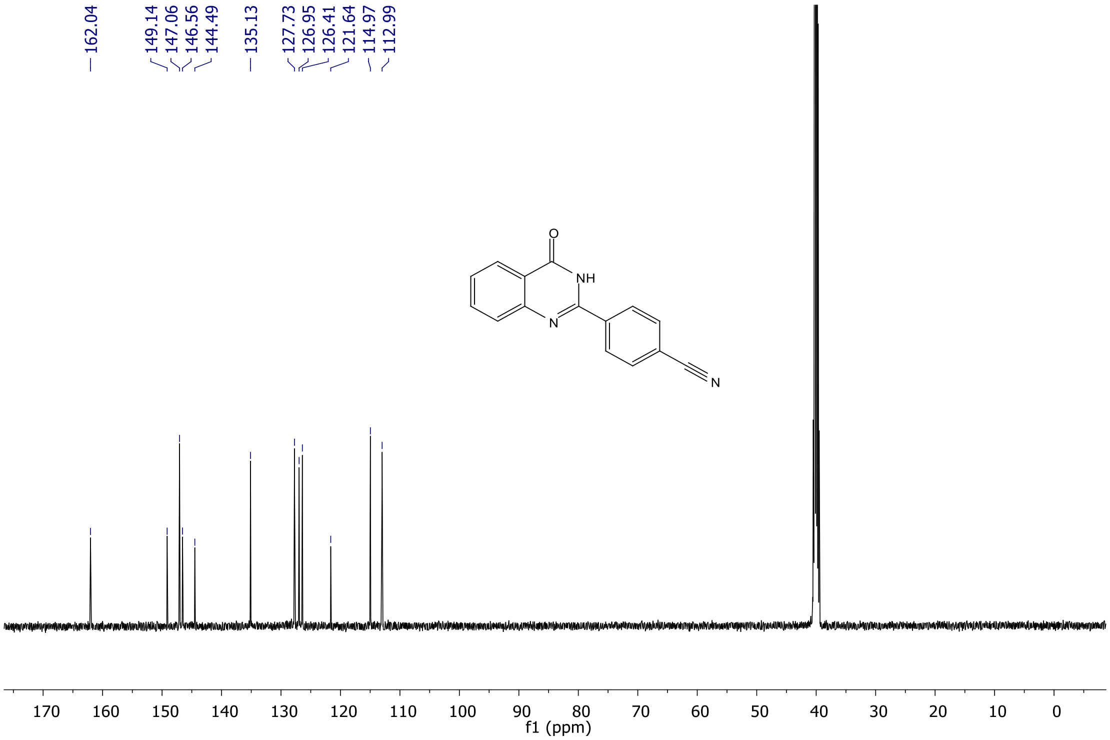

Compound **3o**: ^{13}C NMR (125 MHz, DMSO-d_6).

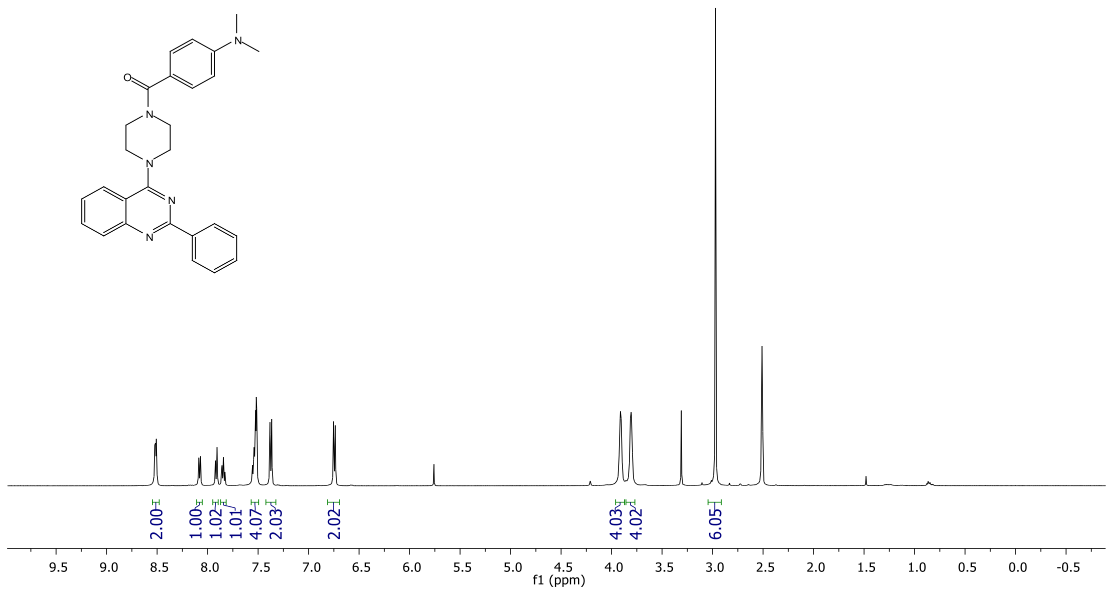

Compound **8a**: ^{1}H NMR (500 MHz, DMSO-d_6).

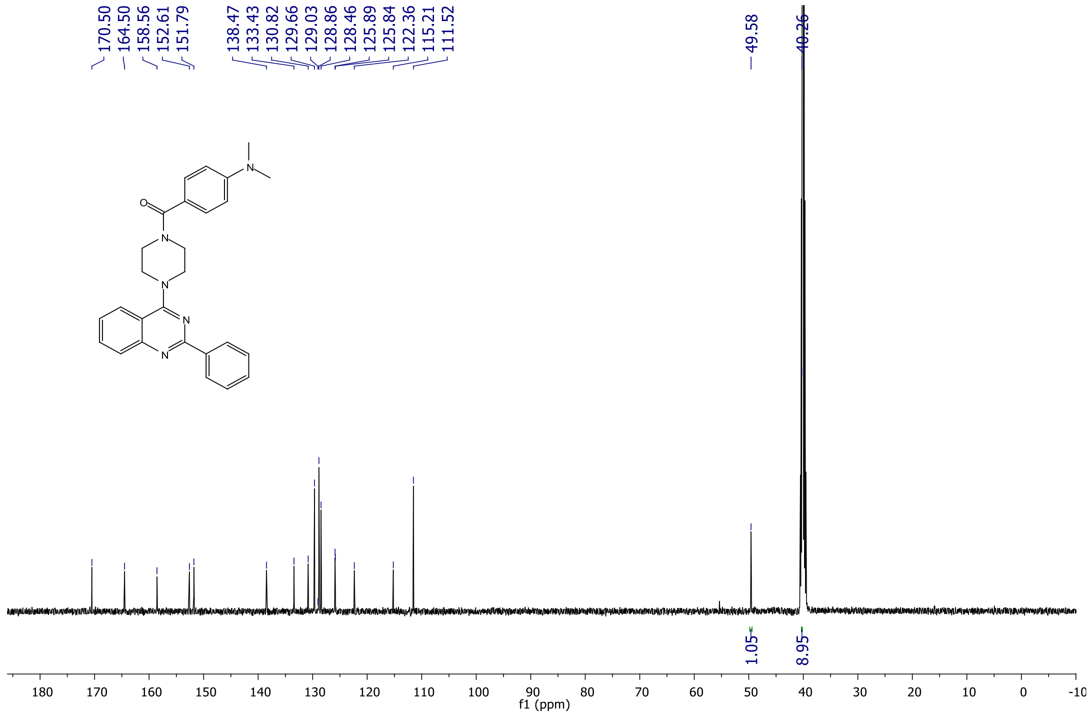

Compound **8a**: ^{13}C NMR (125 MHz, DMSO-d_6).

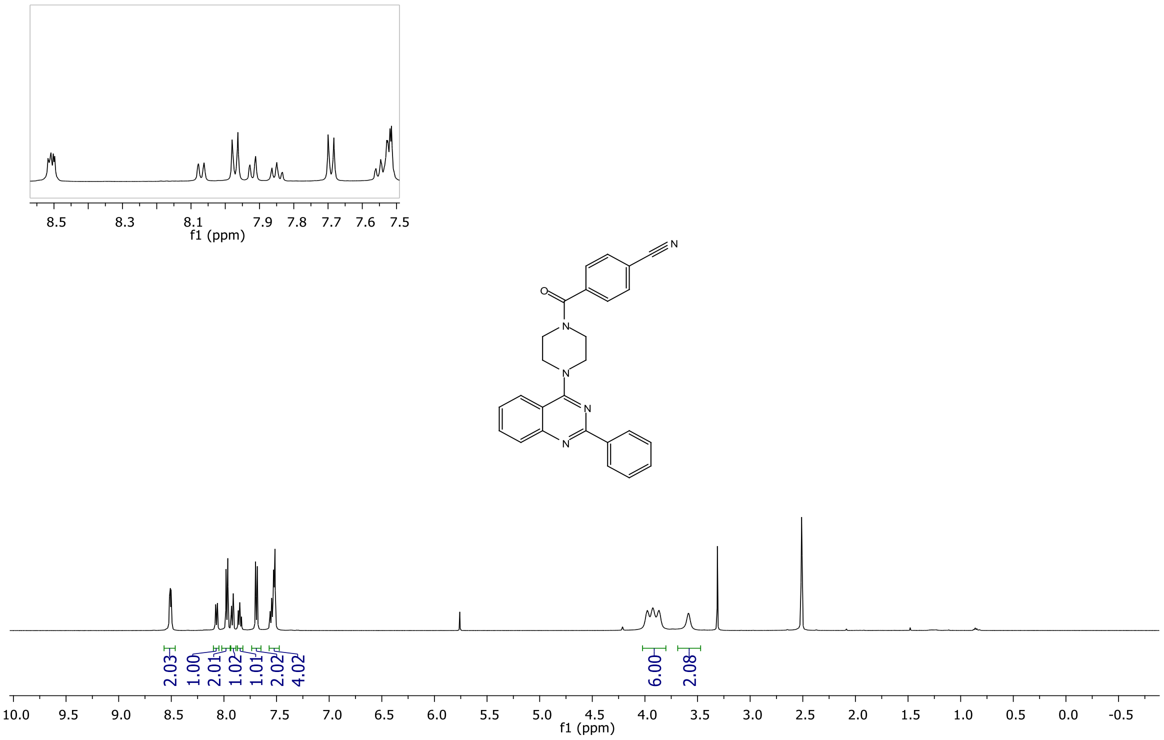

Compound **8b**: ^{1}H NMR (500 MHz, DMSO-d_6).

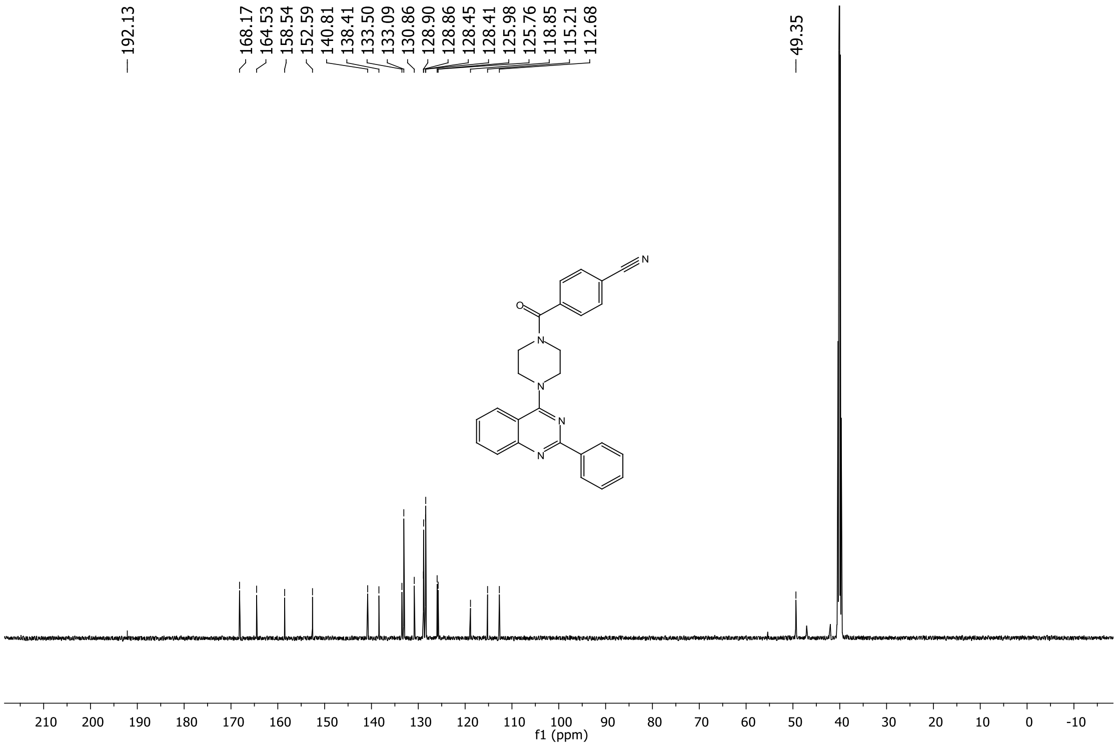

Compound **8b**: ^{13}C NMR (125 MHz, DMSO-d_6).

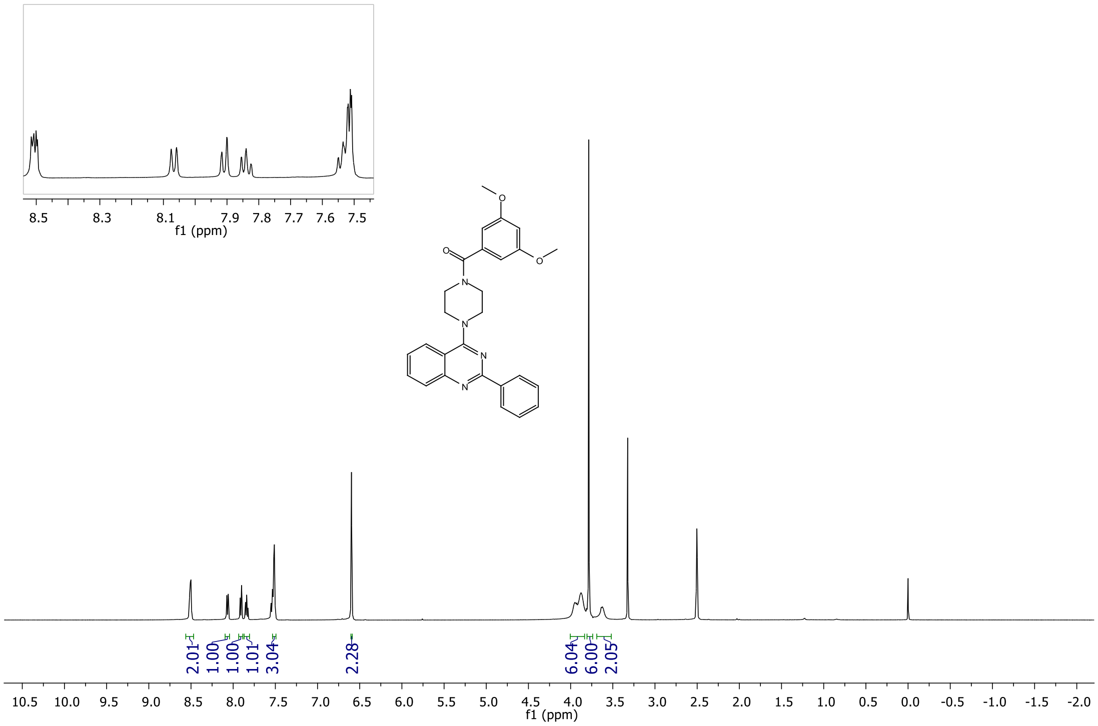

Compound **8c**: ^{1}H NMR (500 MHz, DMSO-d_6).

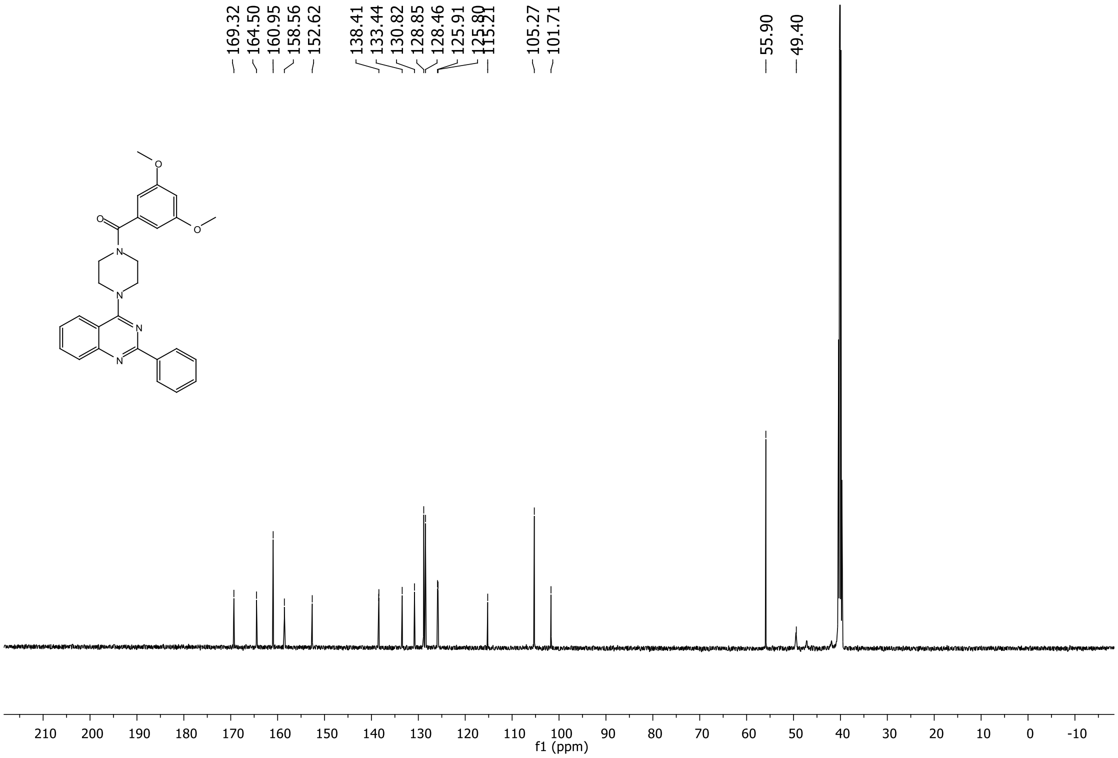

Compound **8c**: ^{13}C NMR (125 MHz, DMSO-d_6).

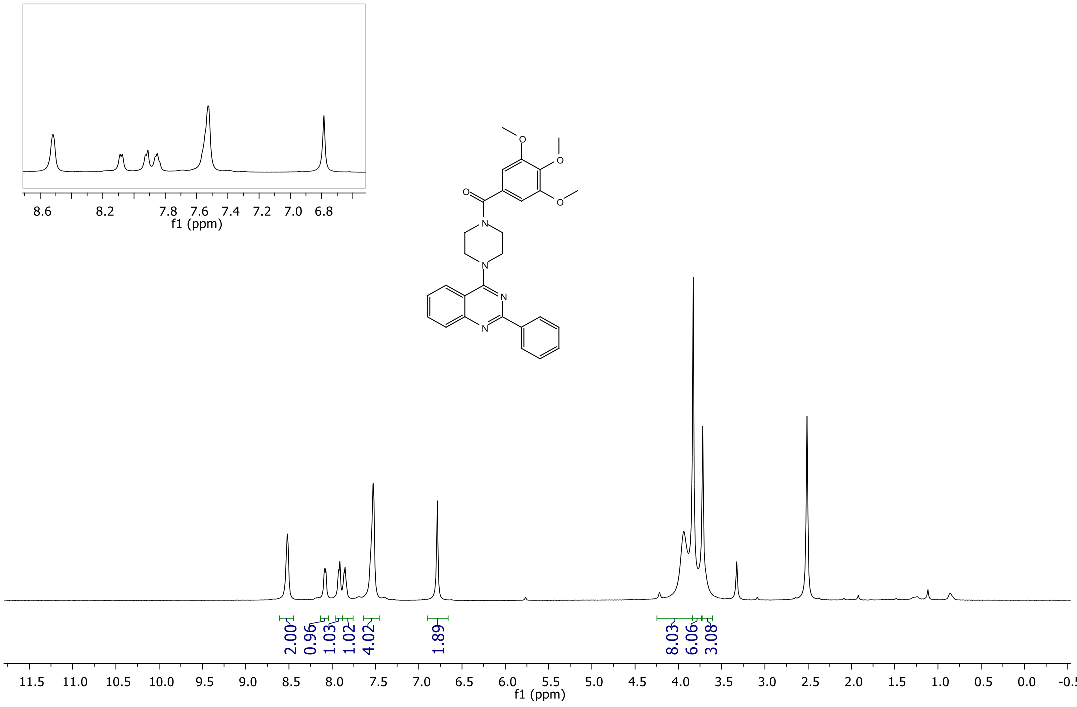

Compound **8d**: ^{1}H NMR (500 MHz, DMSO-d_6).

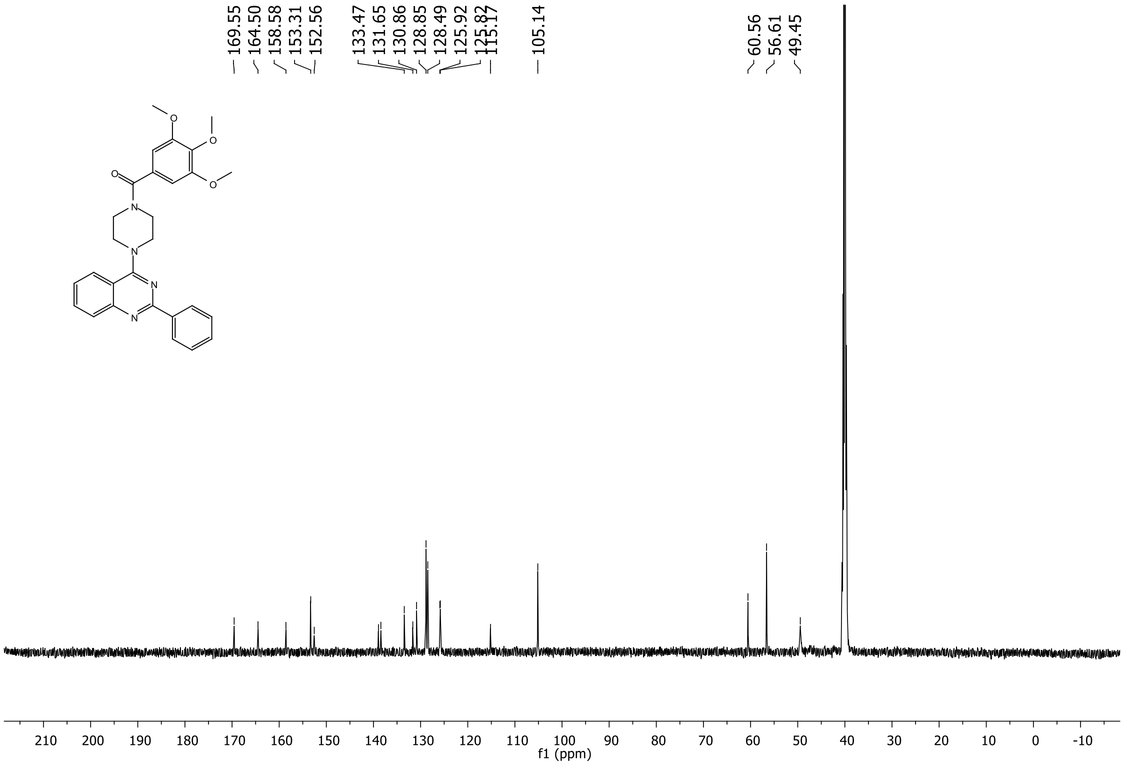

Compound **8d**: ^{13}C NMR (125 MHz, DMSO-d_6).

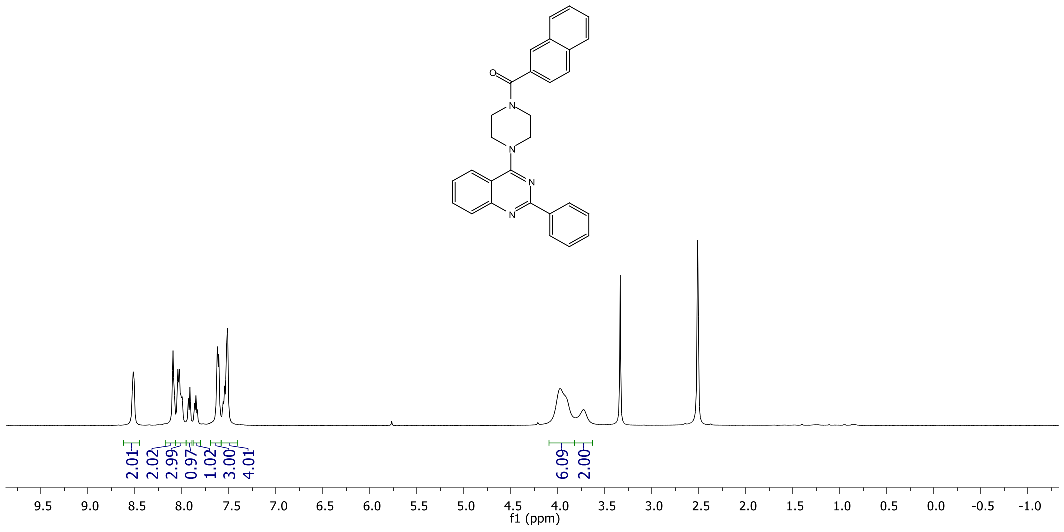

Compound **8e**: ^{1}H NMR (500 MHz, DMSO-d_6).

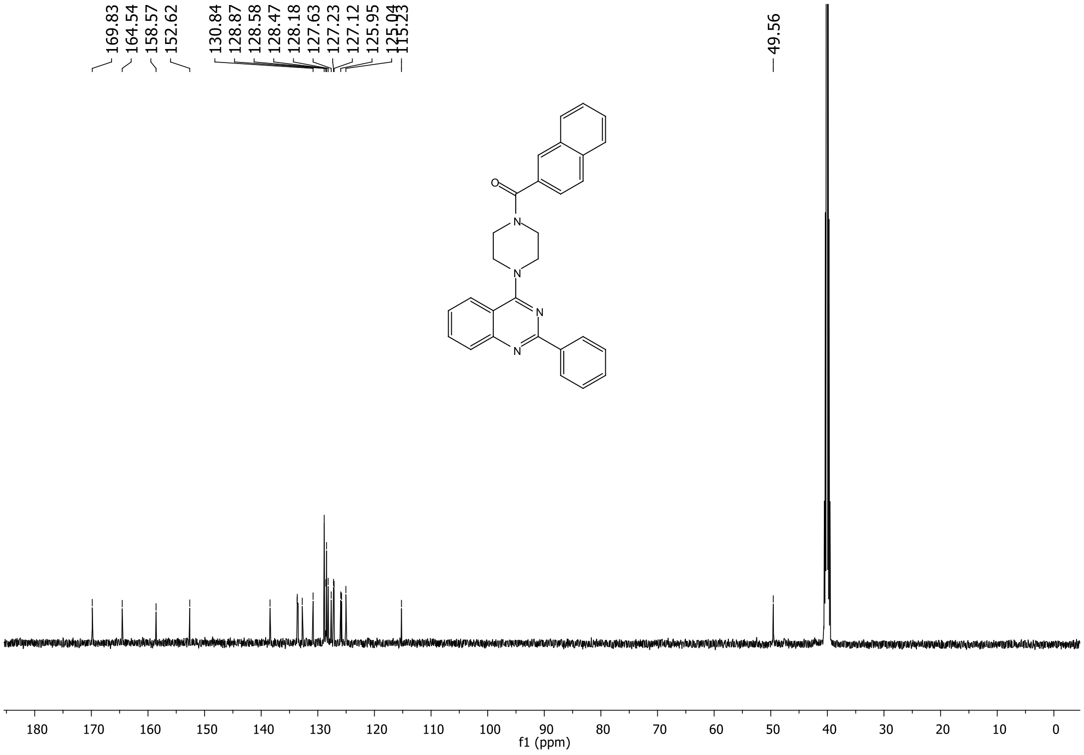

Compound **8e**: ^{13}C NMR (125 MHz, DMSO-d_6).

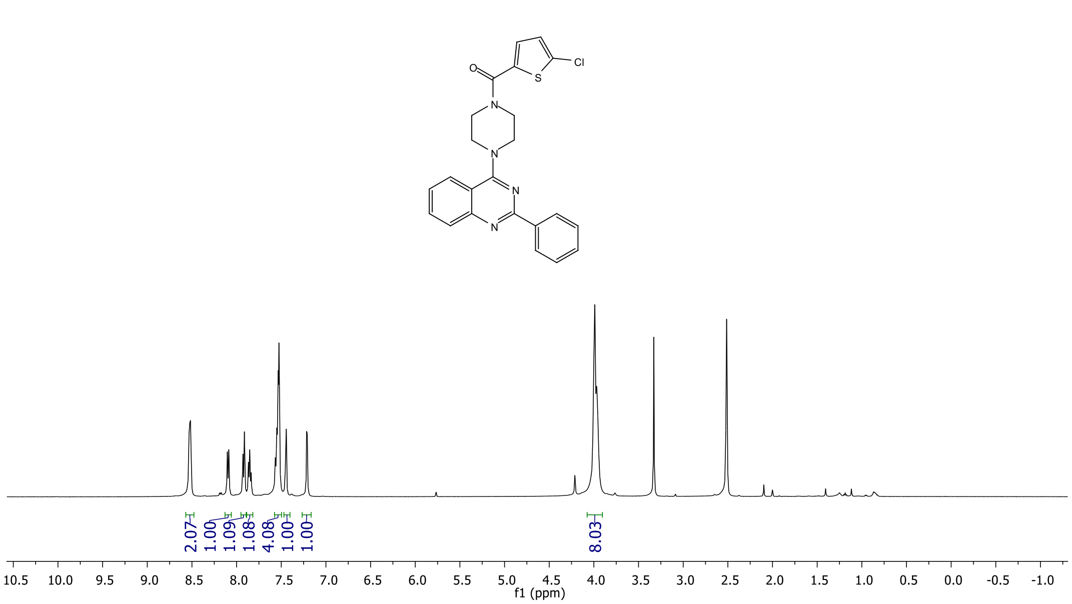

Compound **8f**: ^{1}H NMR (500 MHz, DMSO-d_6).

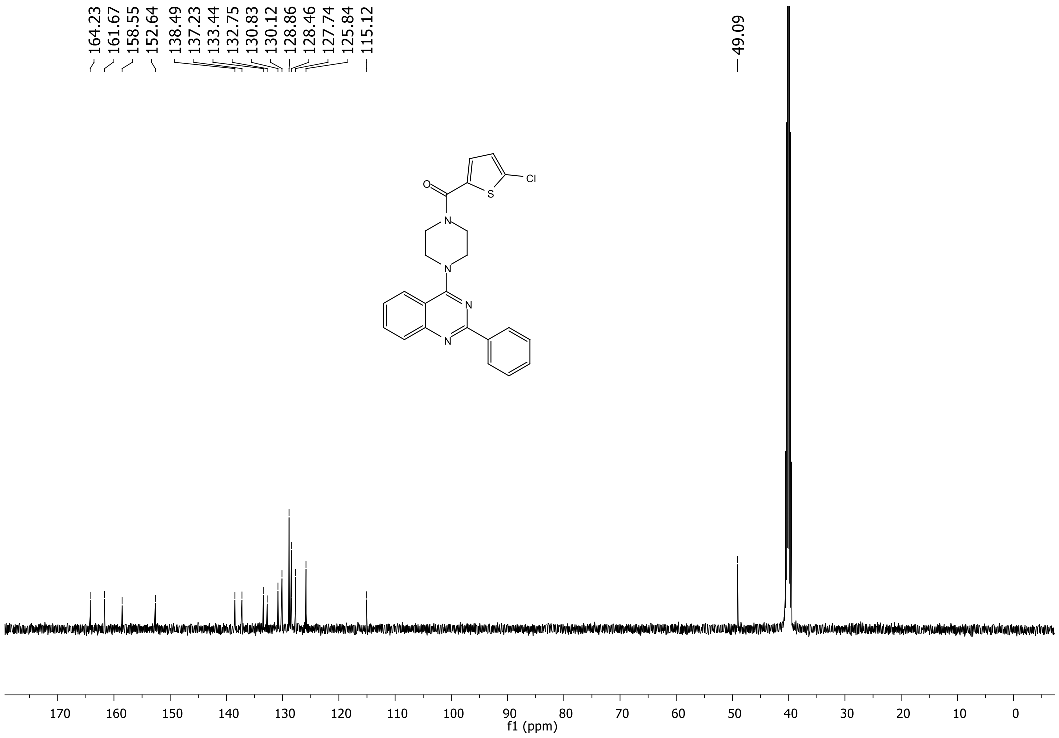

Compound **8f**: ^{13}C NMR (125 MHz, DMSO-d_6).

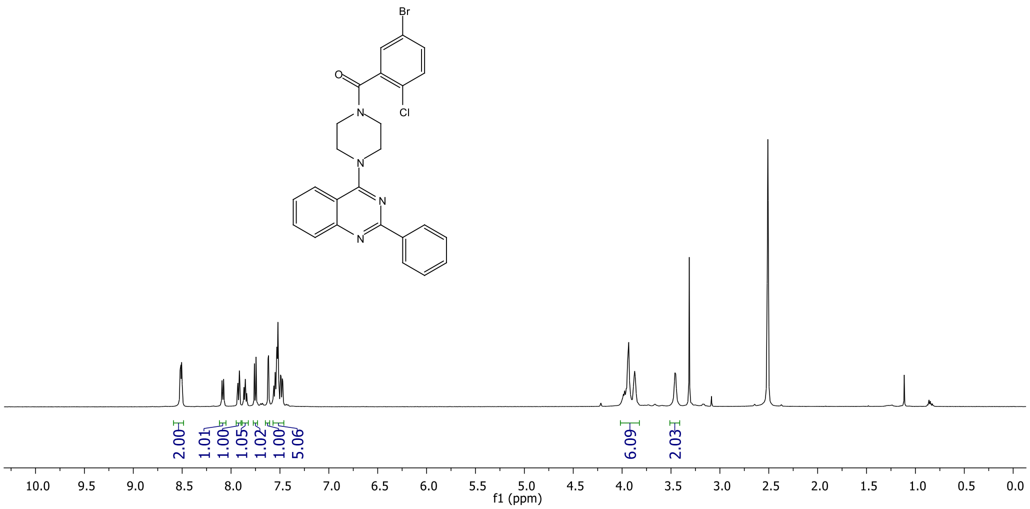

Compound **8g**: ^{1}H NMR (500 MHz, DMSO-d_6).

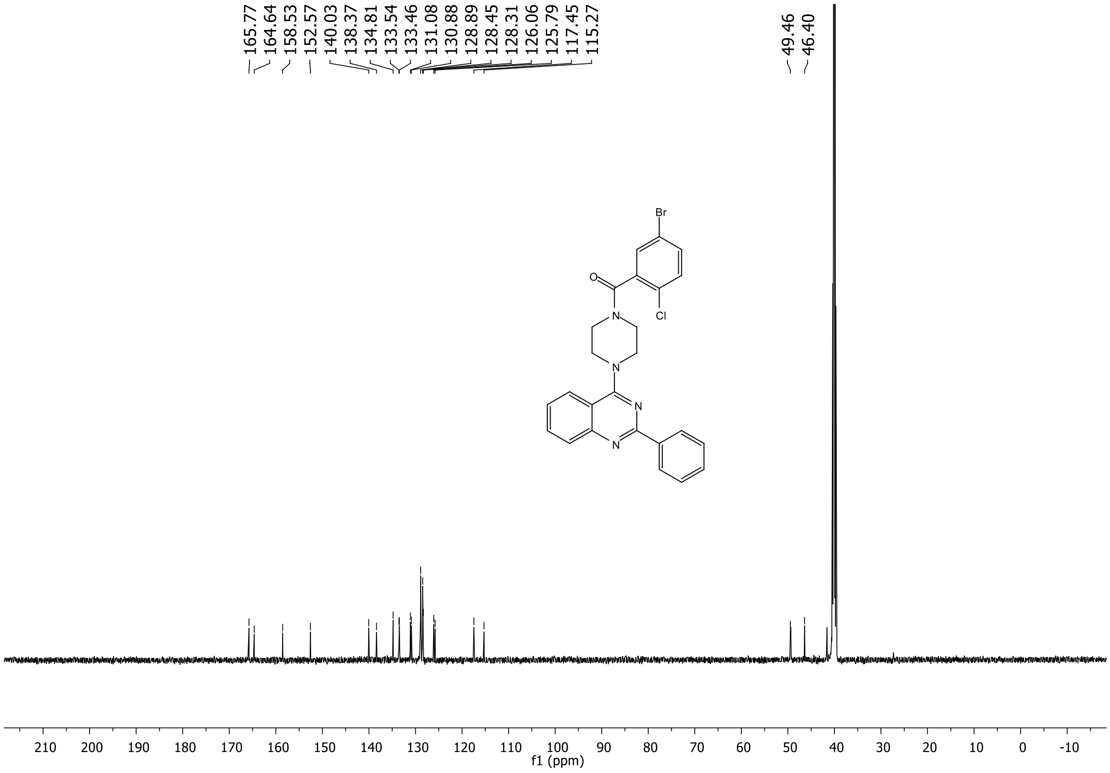

Compound **8g**: ^{13}C NMR (125 MHz, DMSO-d_6).

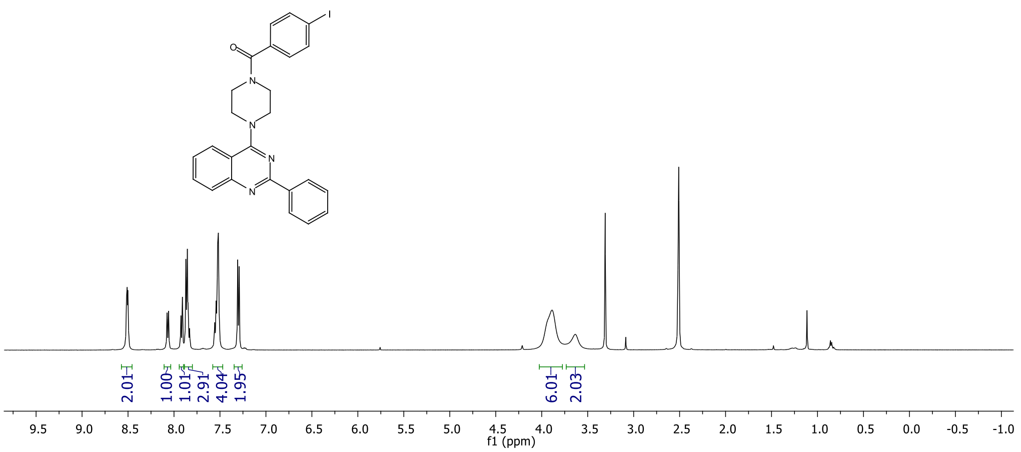

Compound **8h**: ^{1}H NMR (500 MHz, DMSO-d_6).

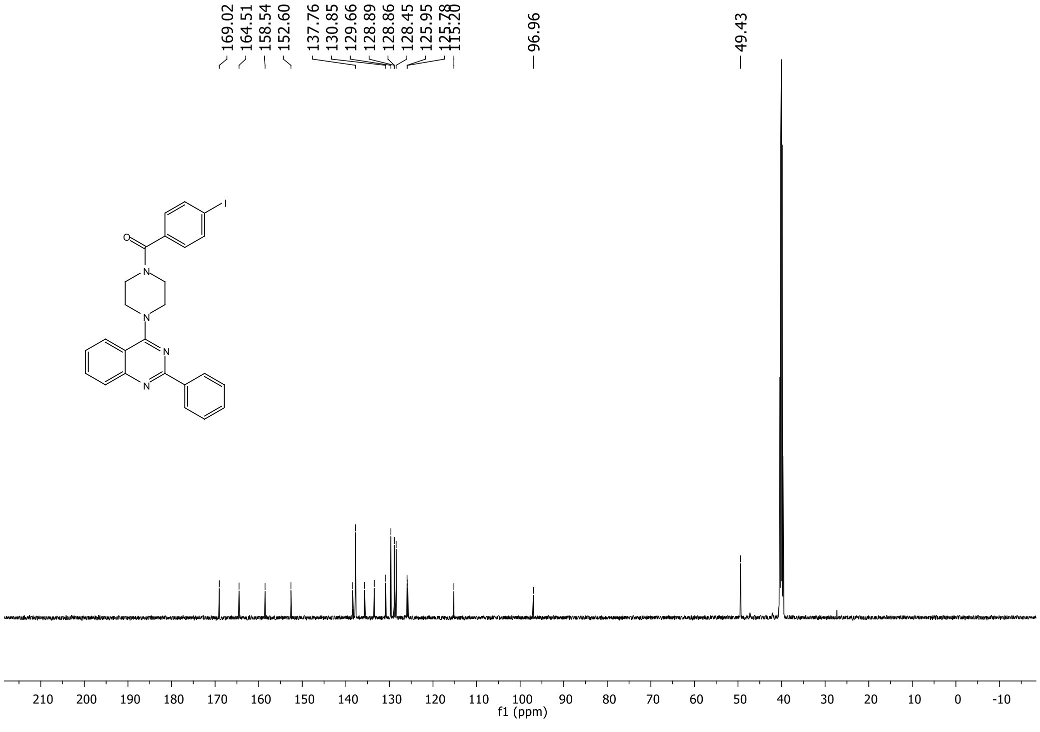

Compound **8h**: ^{13}C NMR (125 MHz, DMSO-d_6).

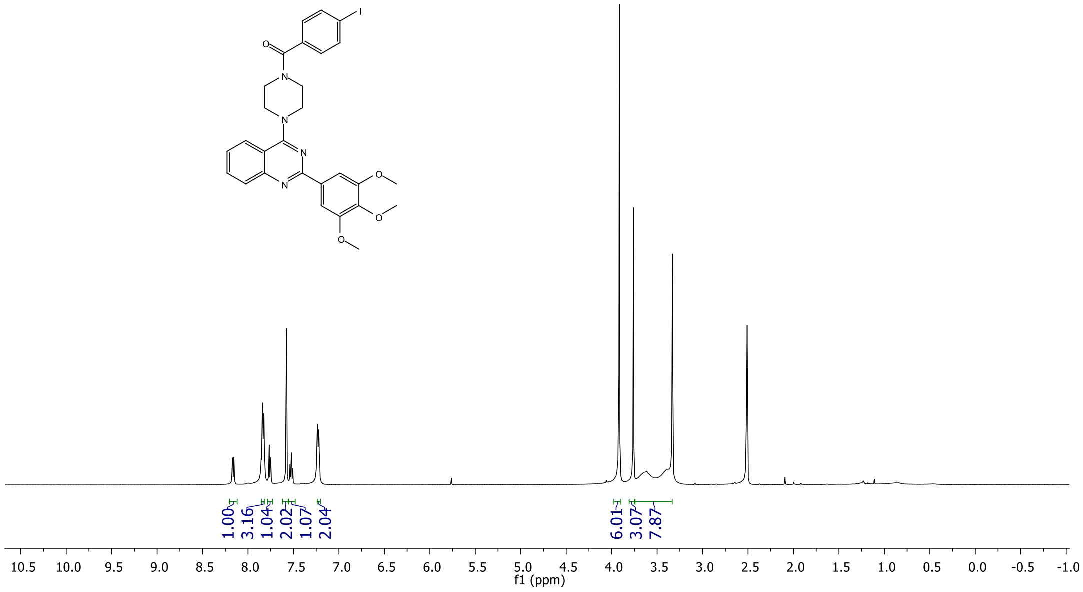

Compound **8i**: ^{1}H NMR (500 MHz, DMSO-d_6).

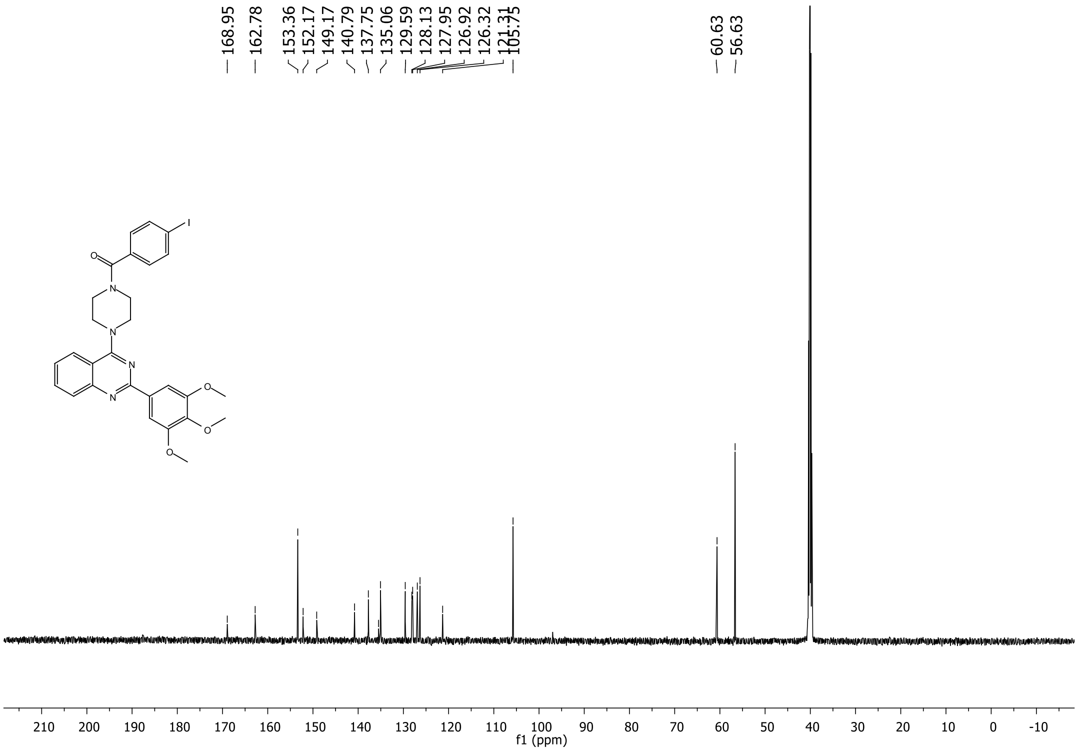

Compound **8i**: ^{13}C NMR (125 MHz, DMSO-d_6).

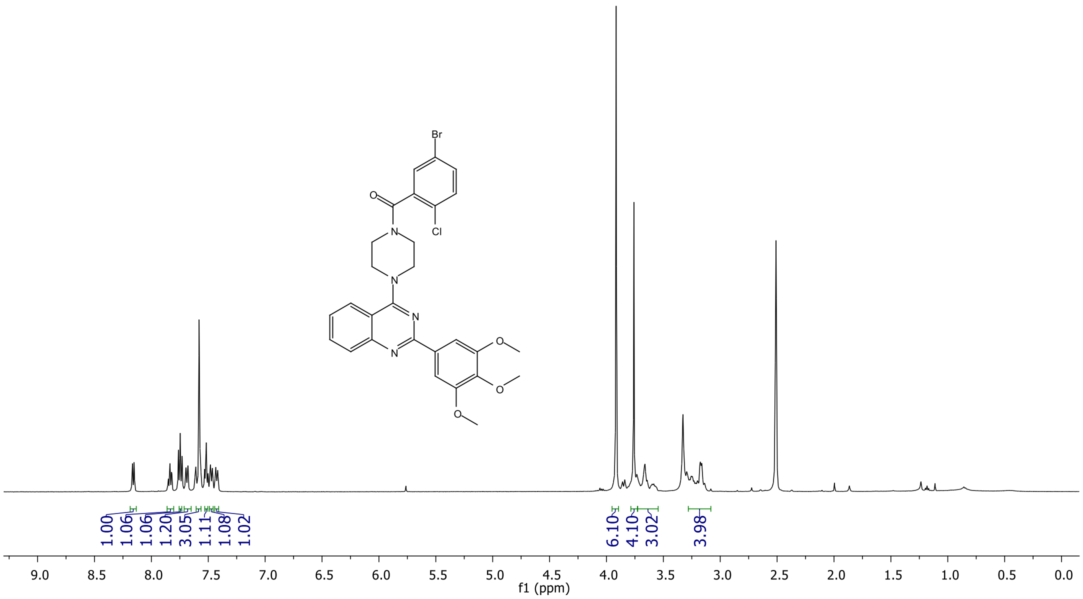

Compound **8j**: ^{1}H NMR (500 MHz, DMSO-d_6).

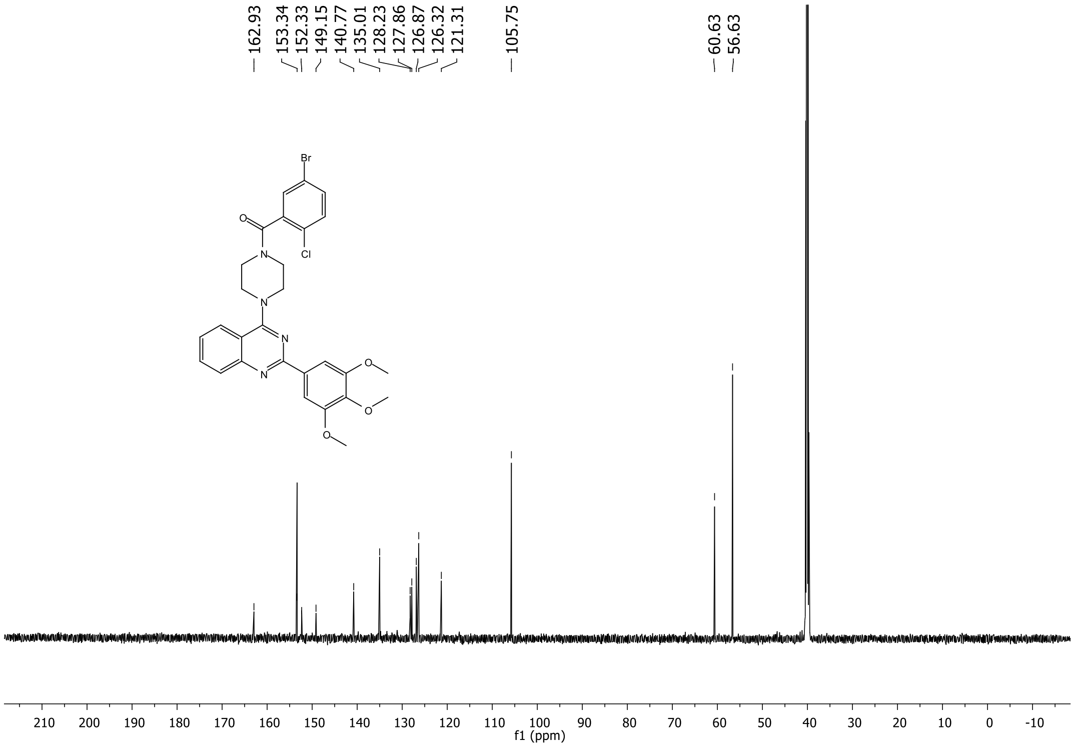

Compound **8j**: ^{13}C NMR (125 MHz, DMSO-d_6).

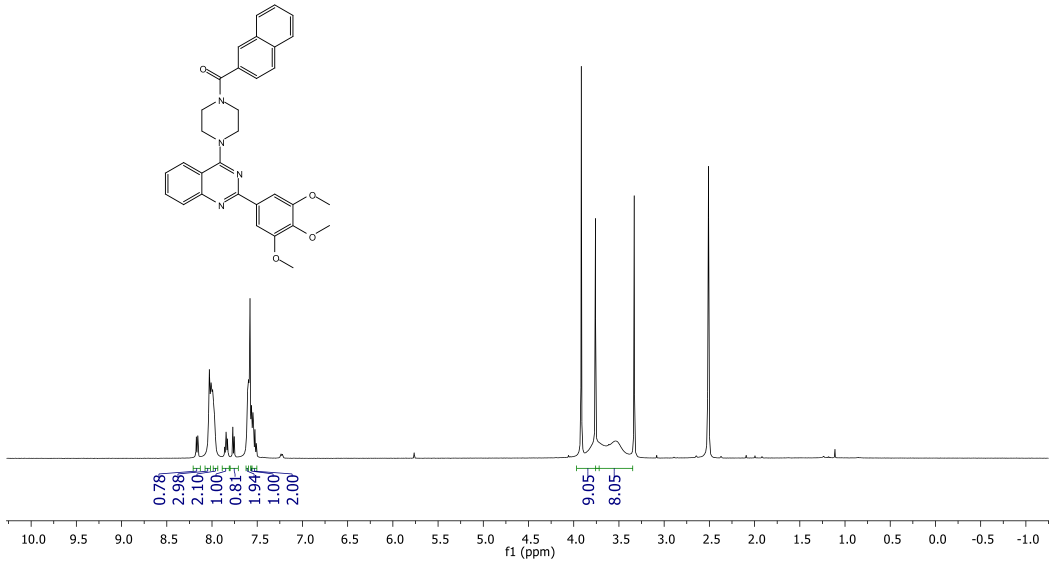

Compound **8k**: ^{1}H NMR (500 MHz, DMSO-d_6).

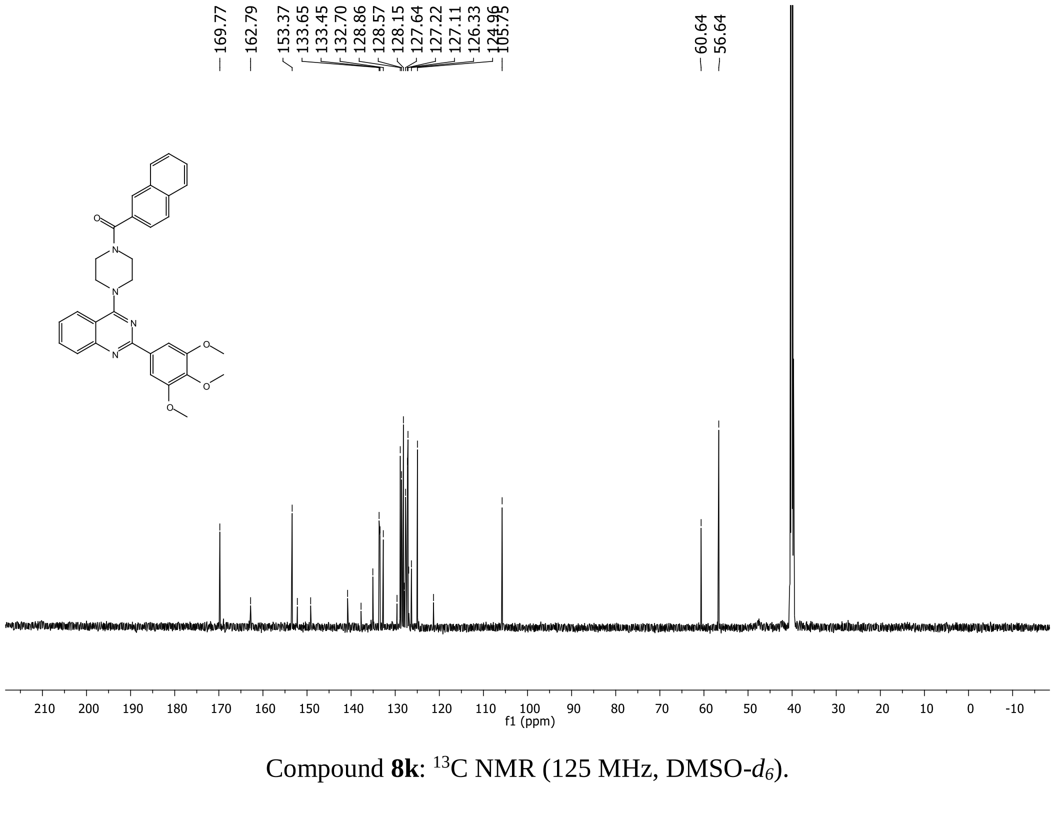

Compound **8k**: ^{13}C NMR (125 MHz, DMSO-d_6).

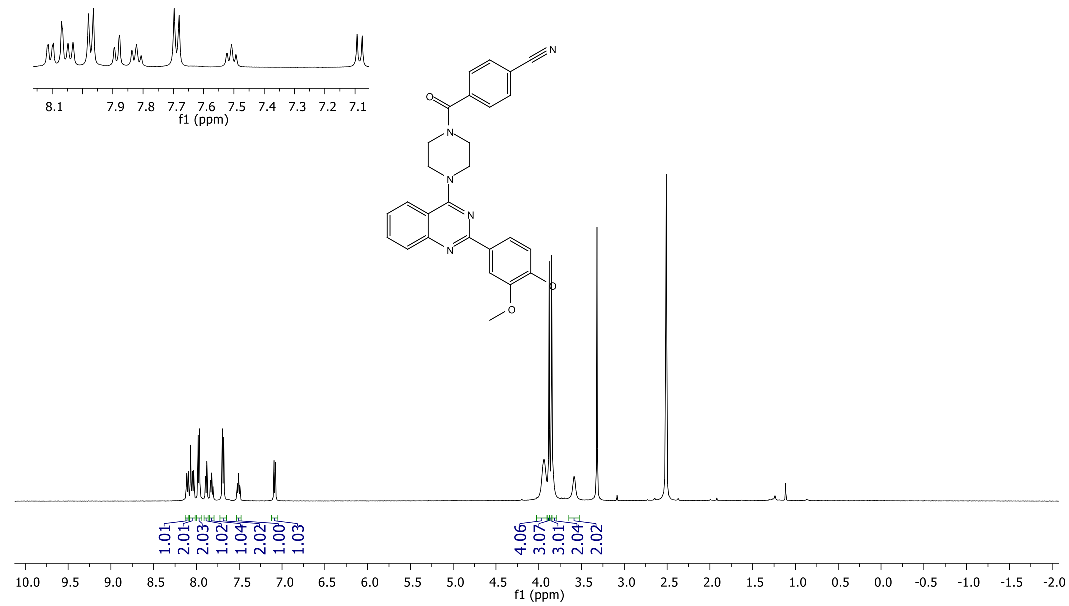

Compound **8l**: ^{1}H NMR (500 MHz, DMSO-d_6).

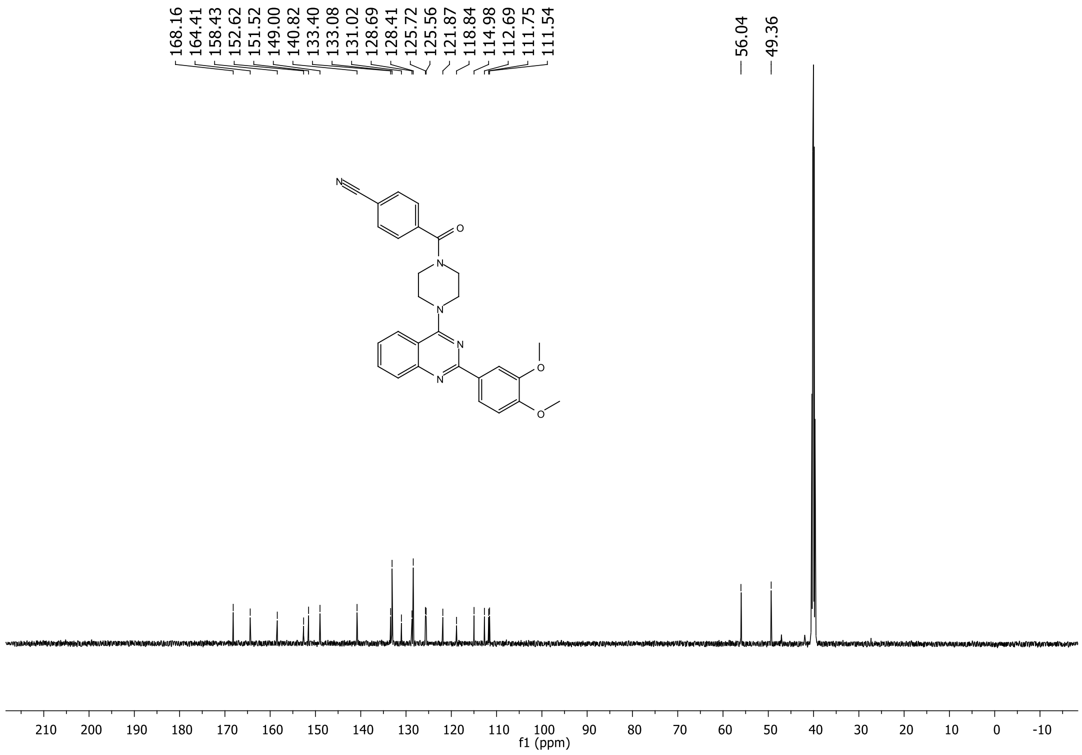

Compound **8l**: ^{13}C NMR (125 MHz, DMSO-d_6).

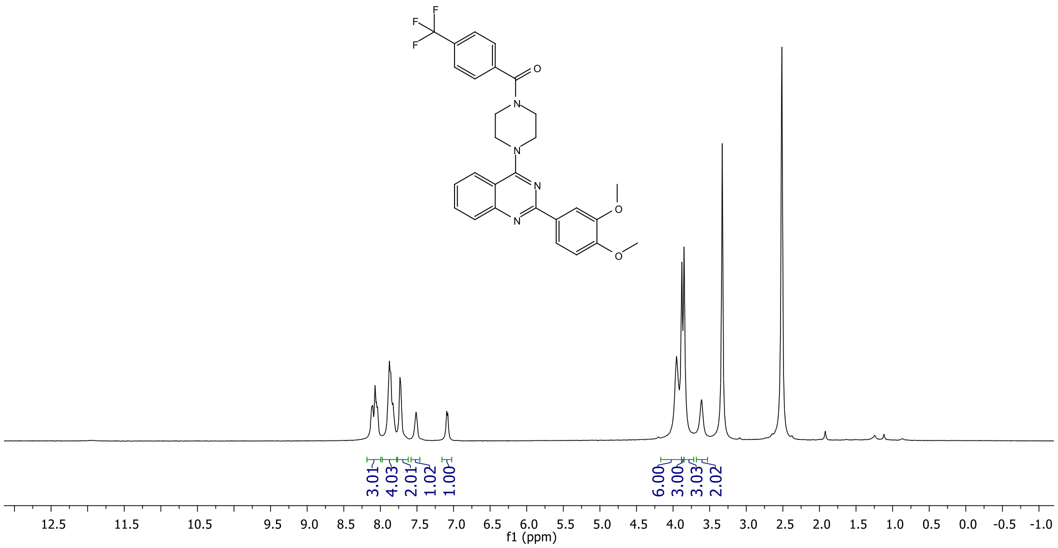

Compound **8m**: ^{1}H NMR (500 MHz, DMSO-d_6).

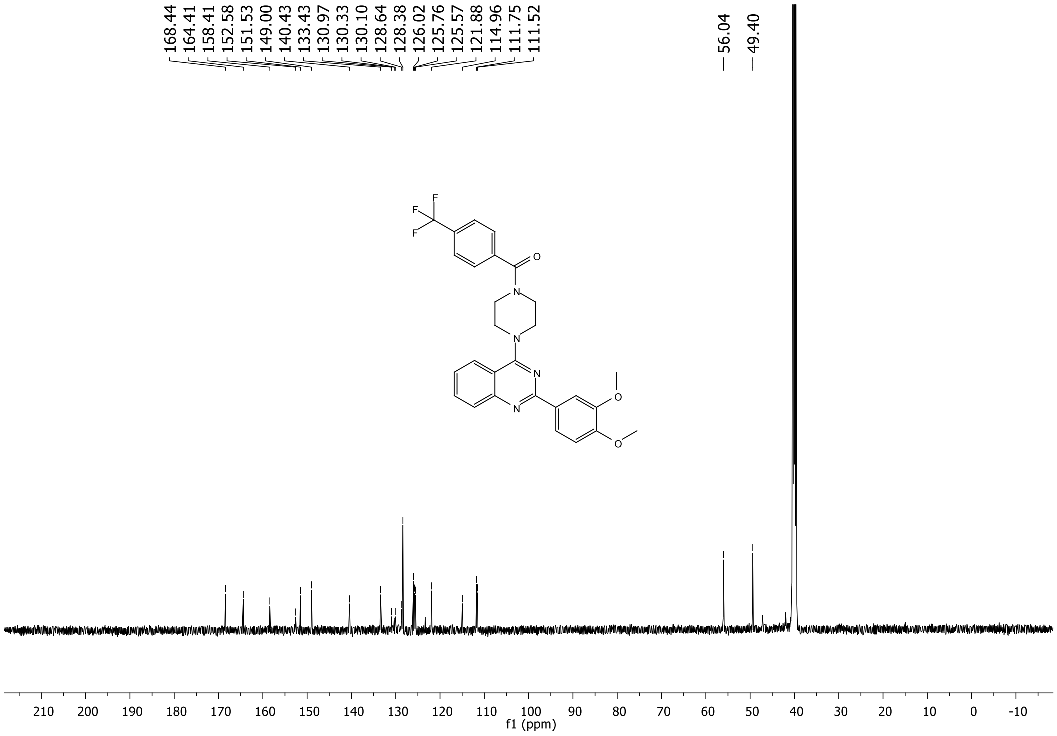

Compound **8m**: ^{13}C NMR (125 MHz, DMSO-d_6)

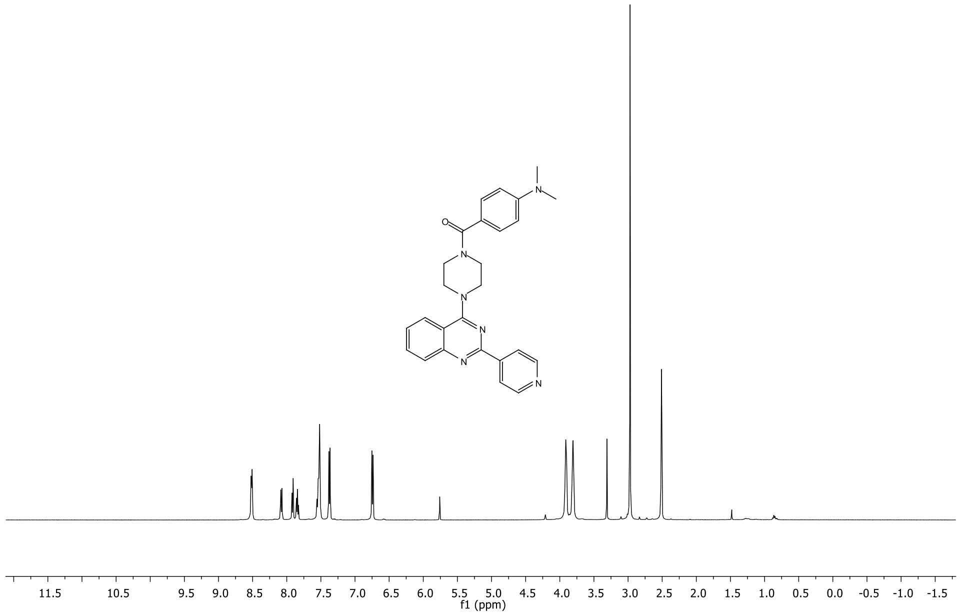

Compound **8n**: ^{1}H NMR (500 MHz, DMSO-d_6).

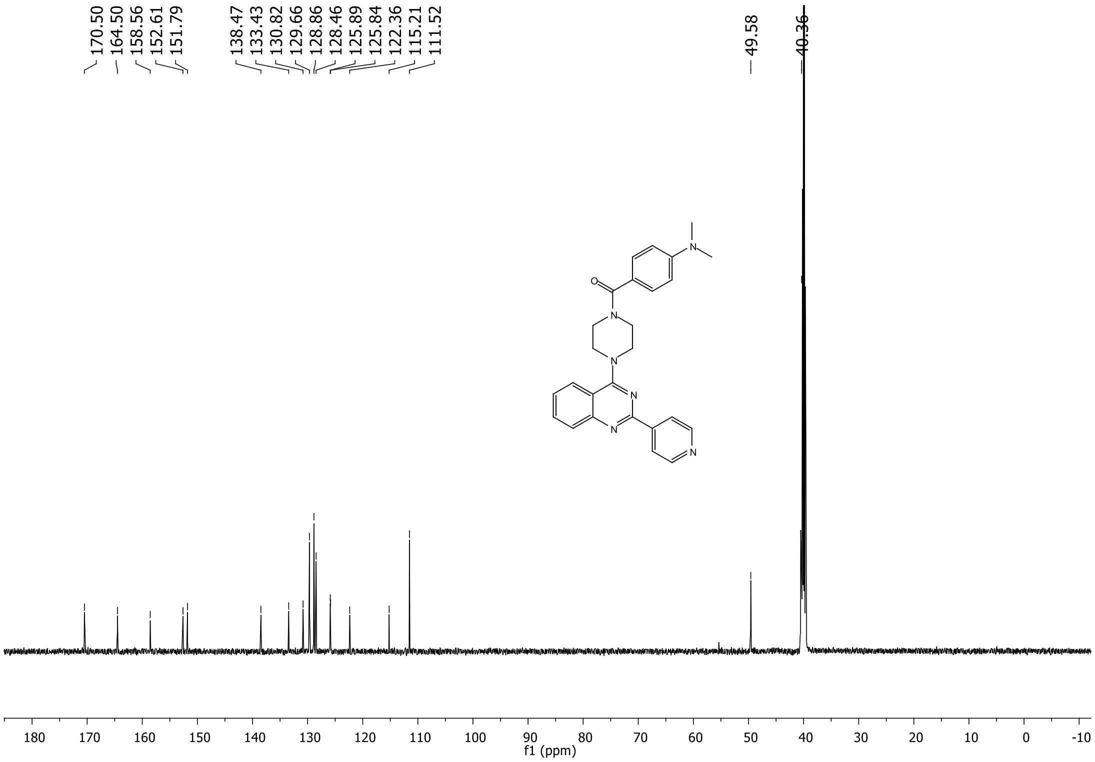

Compound **8n**: ^{13}C NMR (125 MHz, DMSO-d_6).

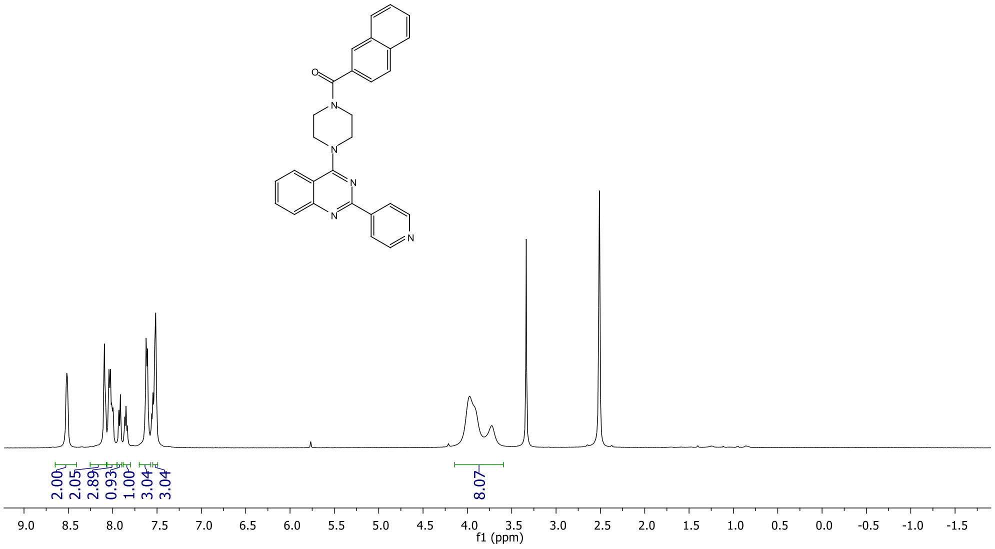

Compound **8o**: ^{1}H NMR (500 MHz, DMSO-d_6).

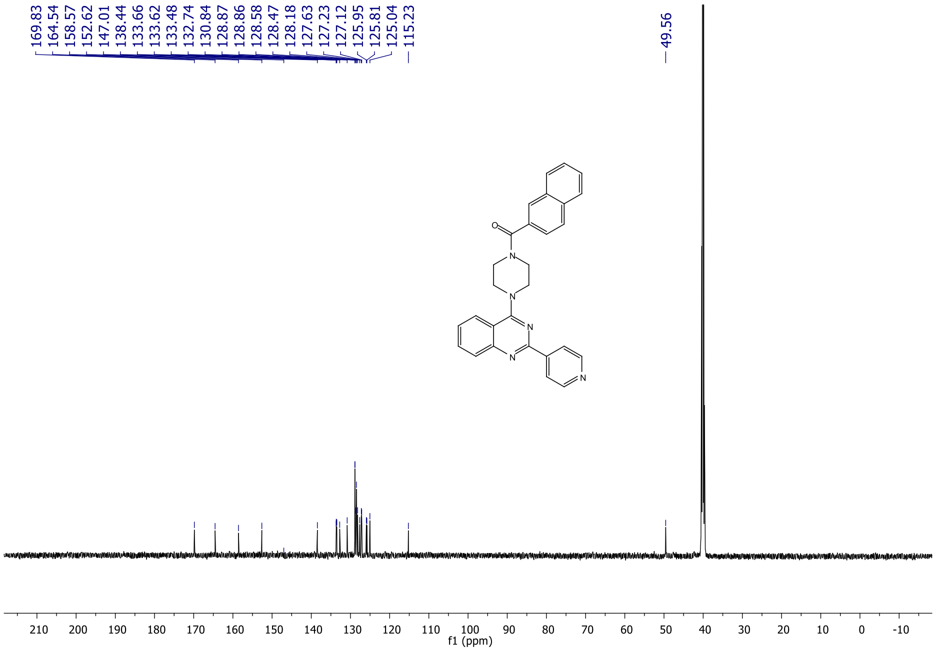

Compound **8o**: ^{13}C NMR (125 MHz, DMSO-d_6).

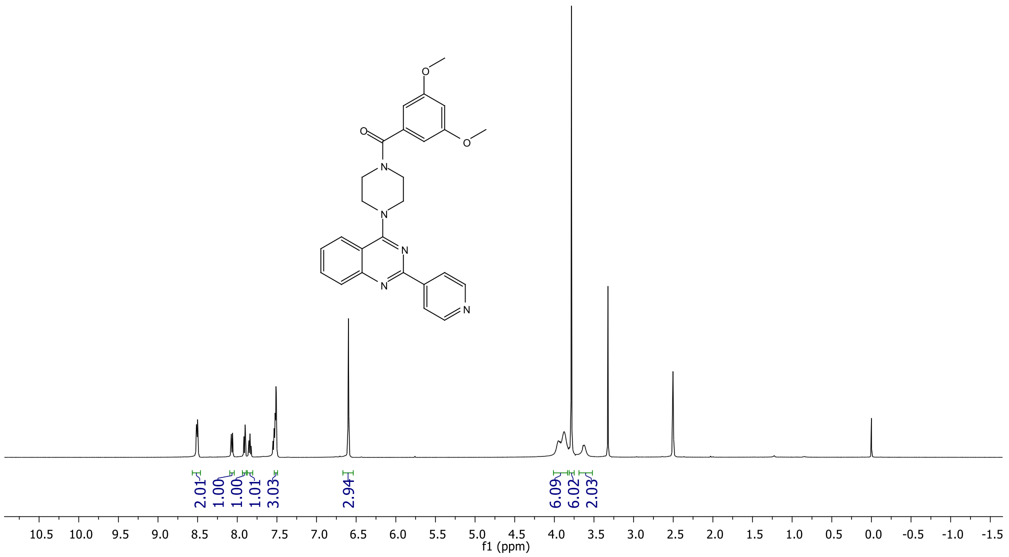

Compound **8p**: ^{1}H NMR (500 MHz, DMSO-d_6).

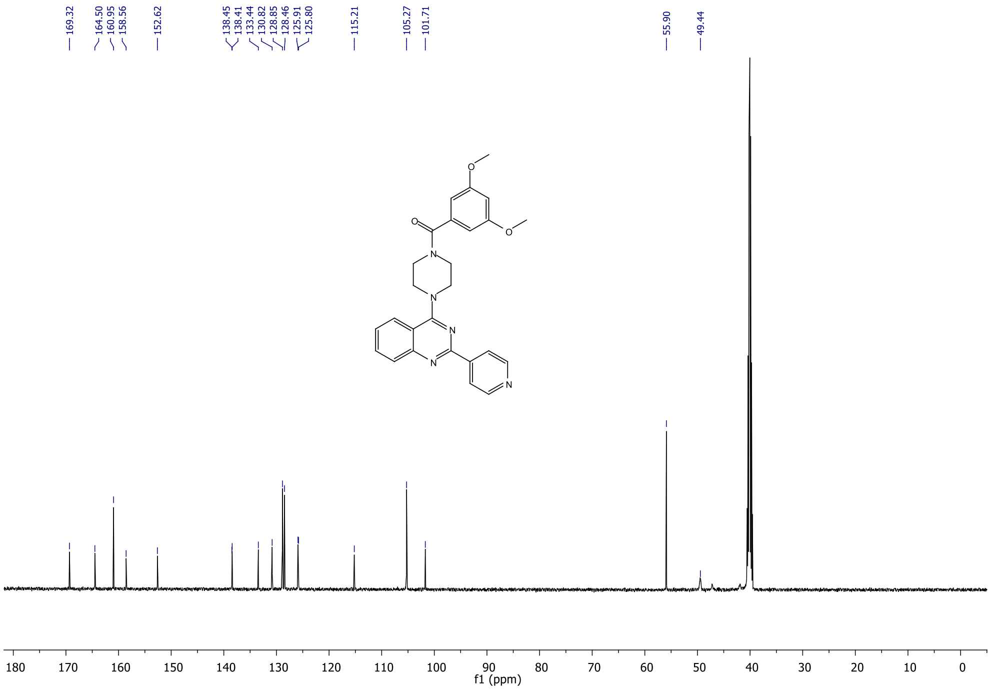

Compound **8p**: ^{13}C NMR (125 MHz, DMSO-d_6).

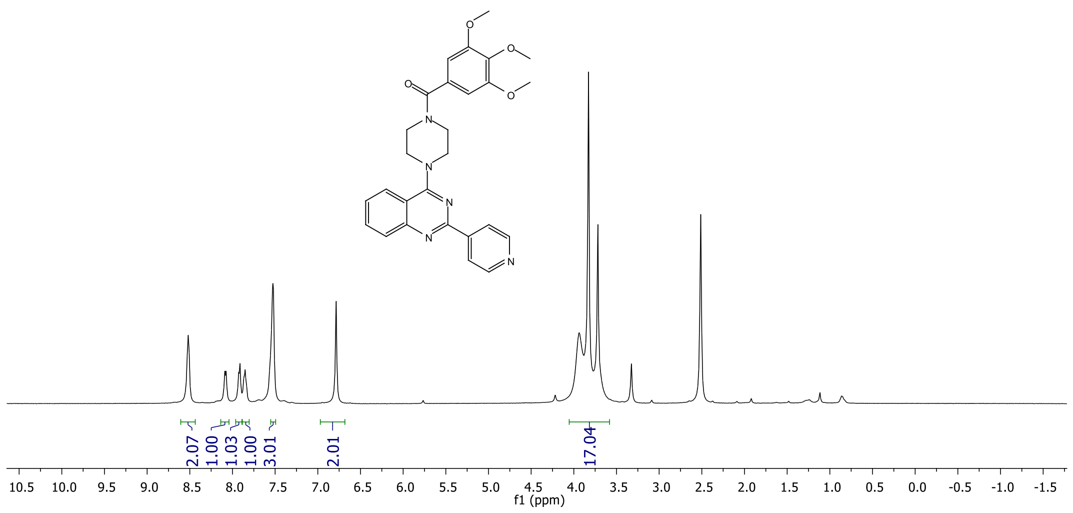

Compound **8q**: ^{1}H NMR (500 MHz, DMSO-d_6).

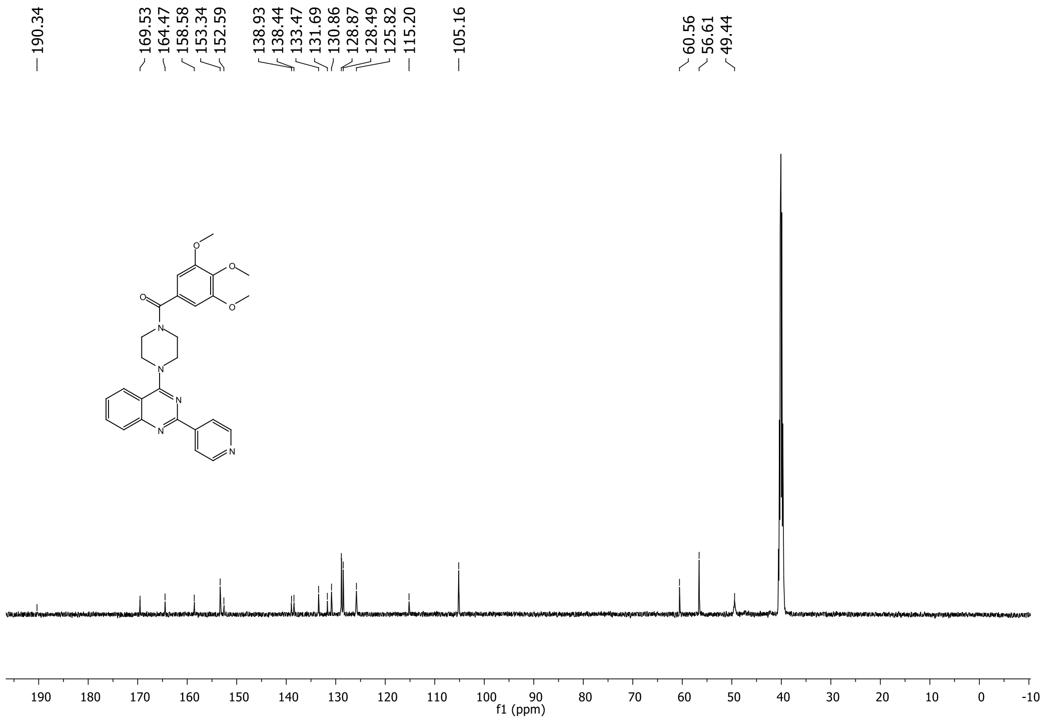

Compound **8q**: ^{13}C NMR (125 MHz, DMSO-d_6).

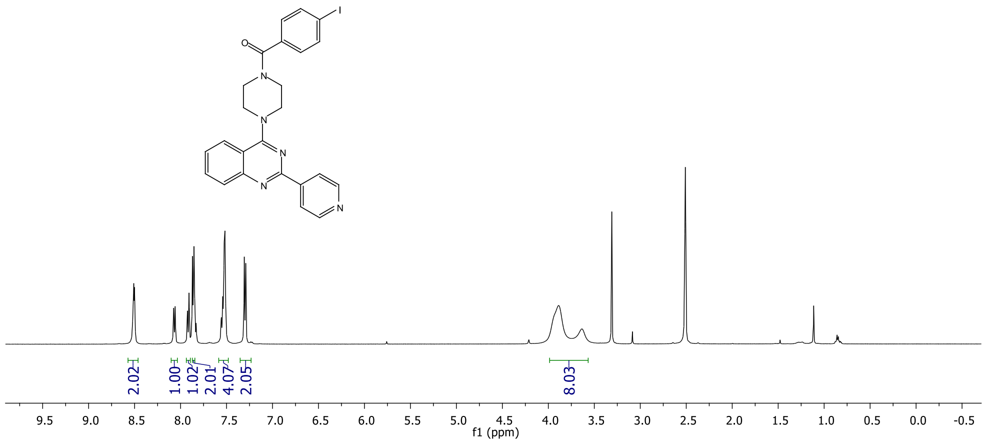

Compound **8r**: ^{1}H NMR (500 MHz, DMSO-d_6).

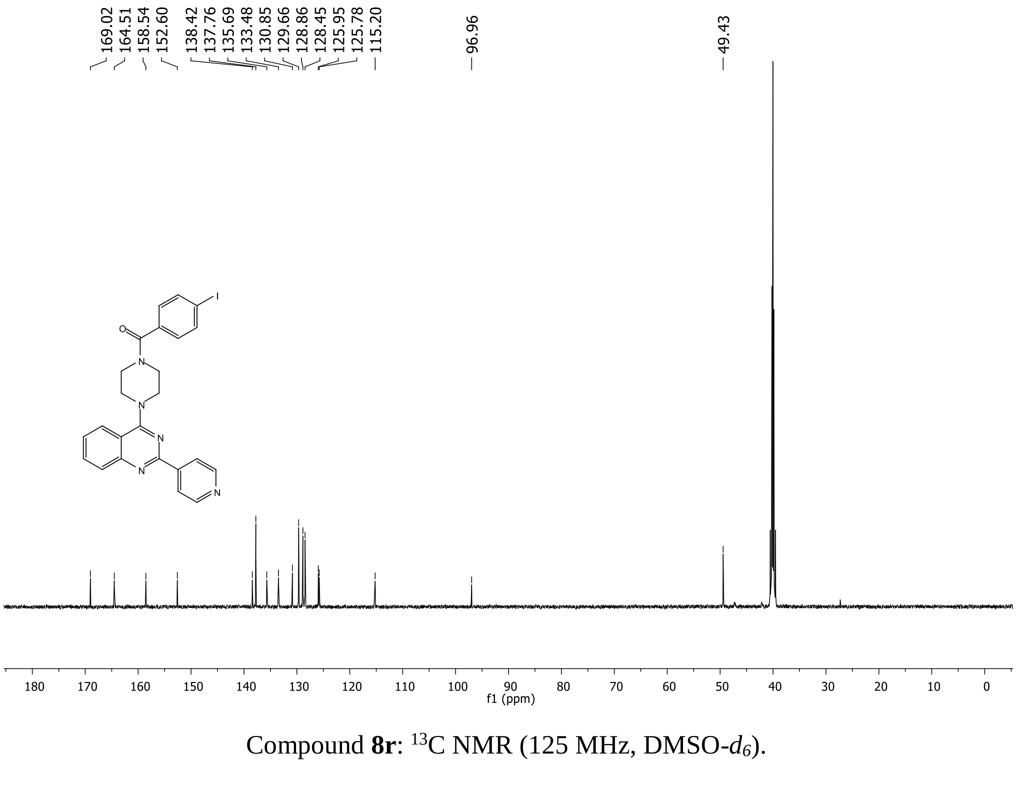

Compound **8r**: ^{13}C NMR (125 MHz, DMSO-d_6).

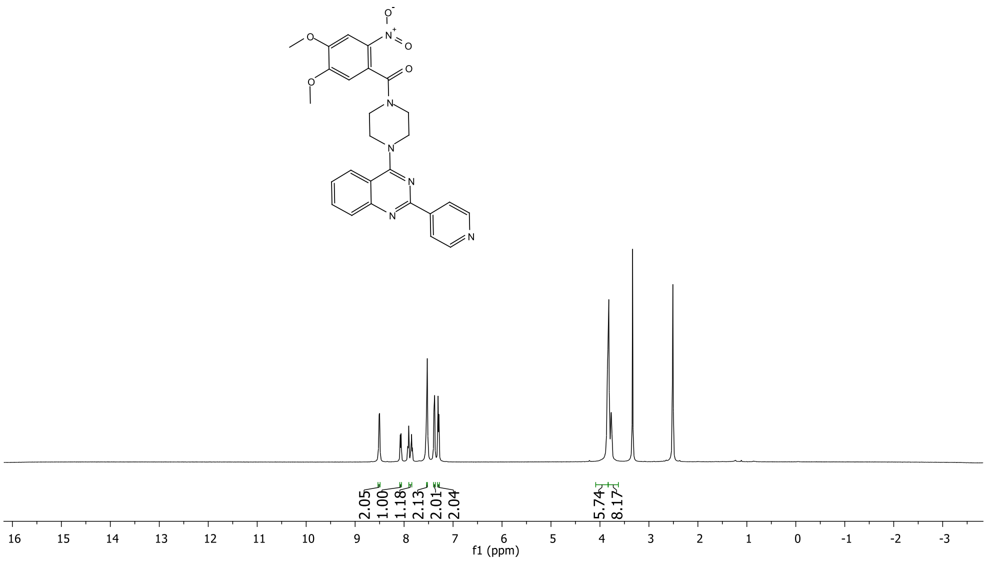

Compound **8s**: ^{1}H NMR (500 MHz, DMSO-d_6).

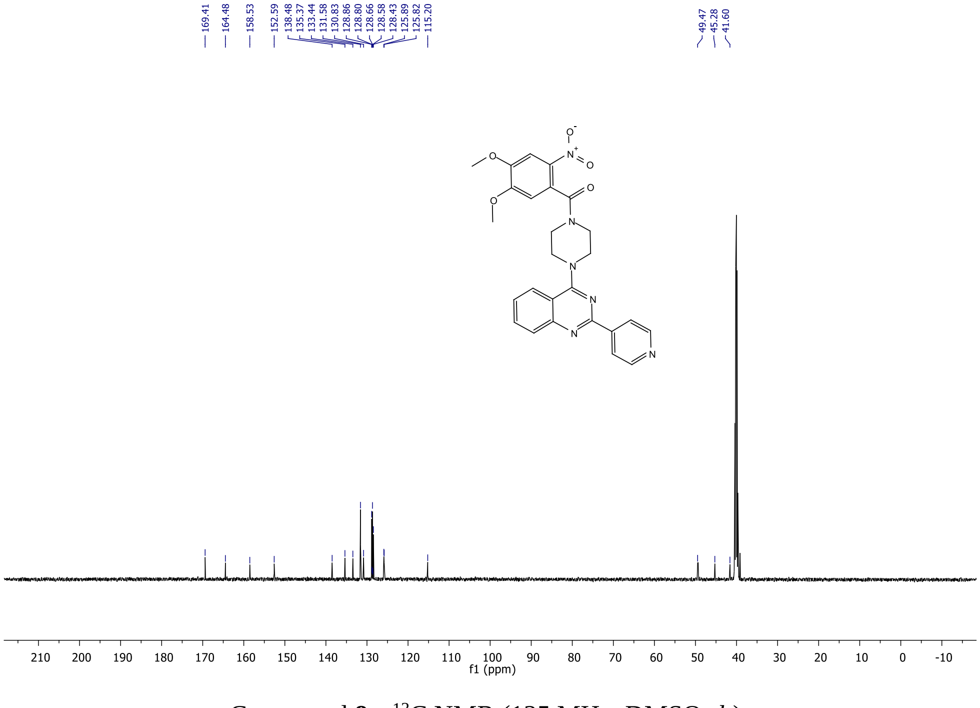

Compound **8s**: ^{13}C NMR (125 MHz, DMSO-d_6).

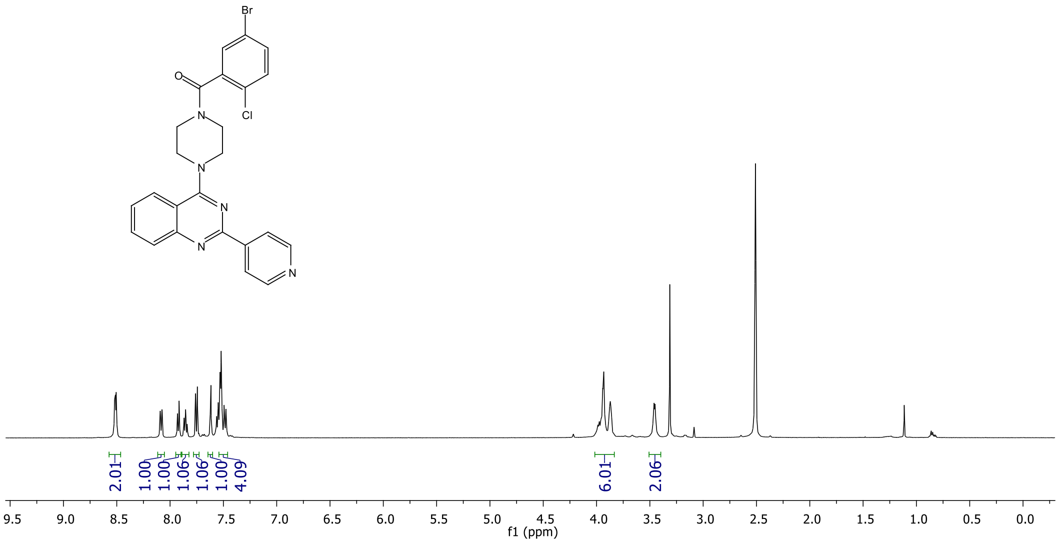

Compound **8t**: ^{1}H NMR (500 MHz, DMSO-d_6).

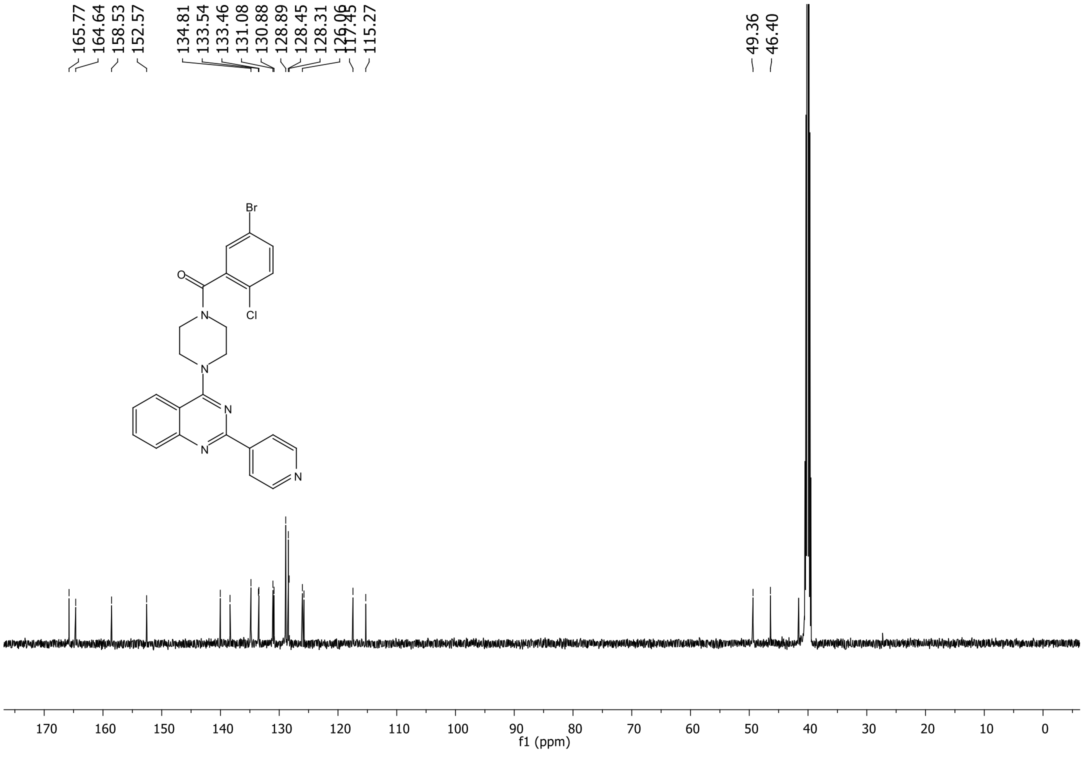

Compound **8t**: ^{13}C NMR (125 MHz, DMSO-d_6).

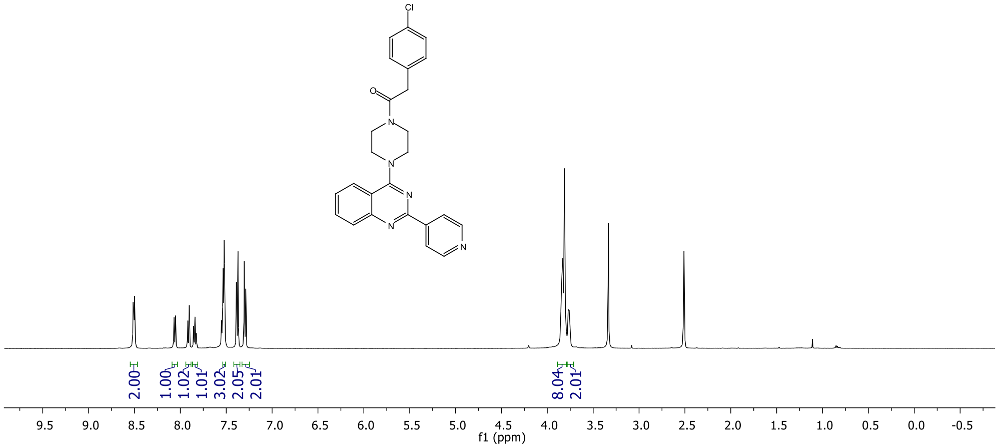

Compound **8u**: ^{1}H NMR (500 MHz, DMSO-d_6).

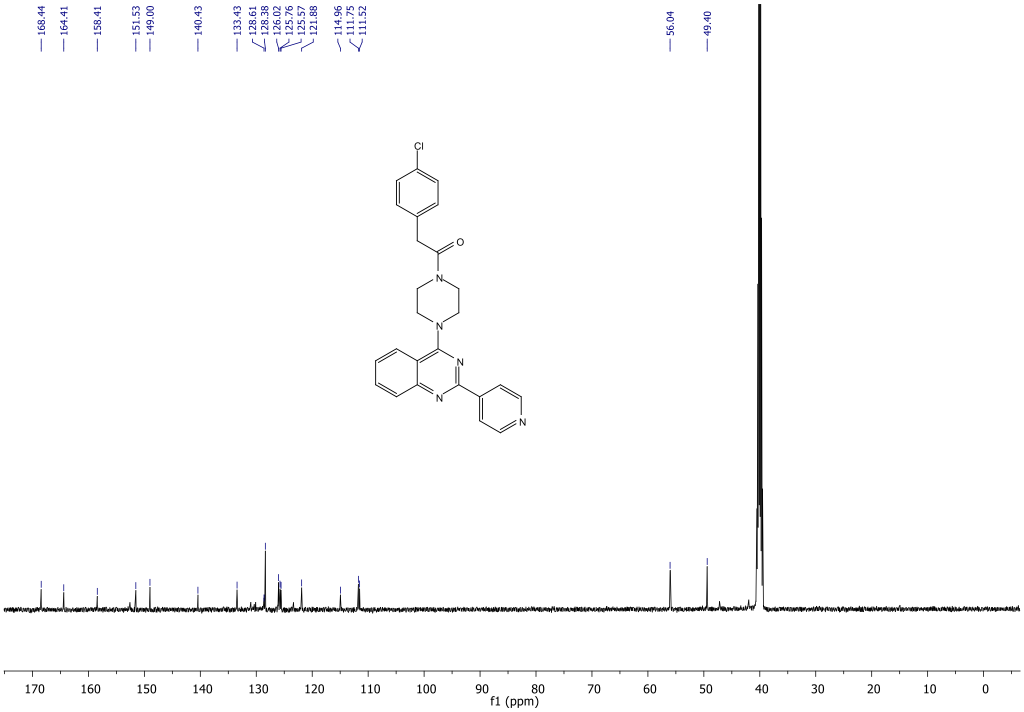

Compound **8u**: ^{13}C NMR (125 MHz, DMSO-d_6).

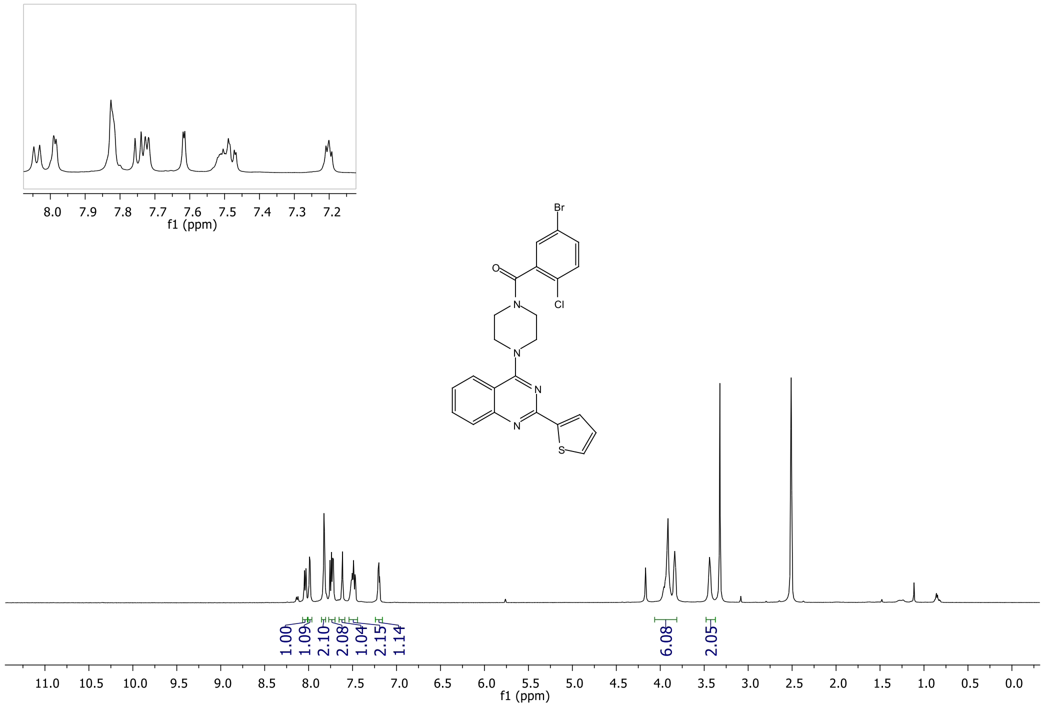

Compound **8v**: ^{1}H NMR (500 MHz, DMSO-d_6).

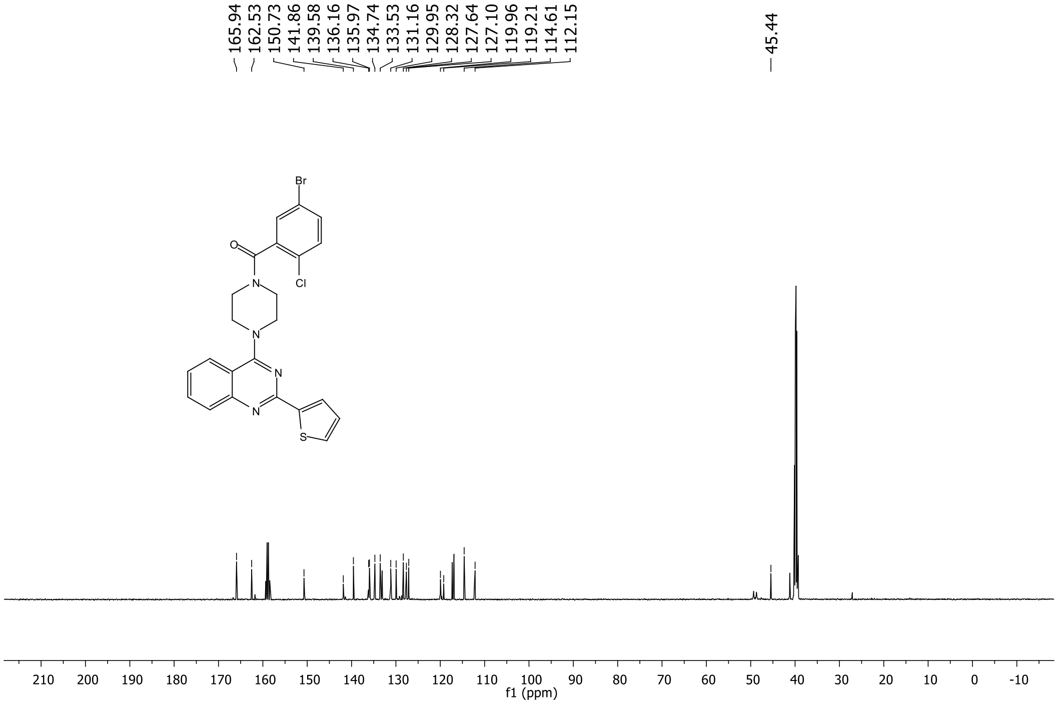

Compound **8v**: ^{13}C NMR (125 MHz, DMSO-d_6).

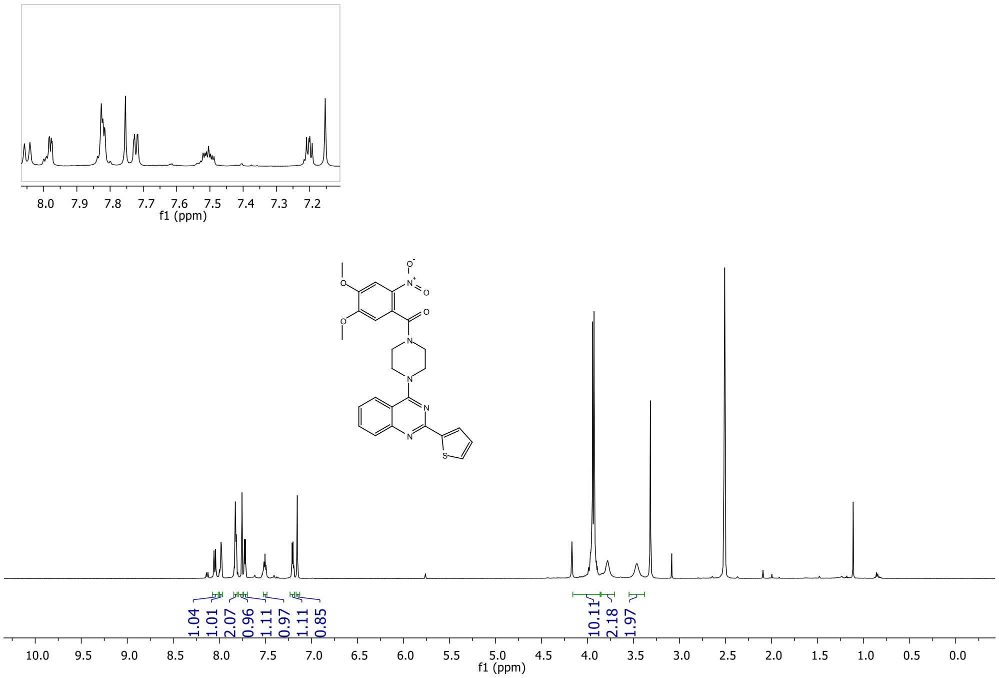

Compound **8w**: ^{1}H NMR (500 MHz, DMSO-d_6).

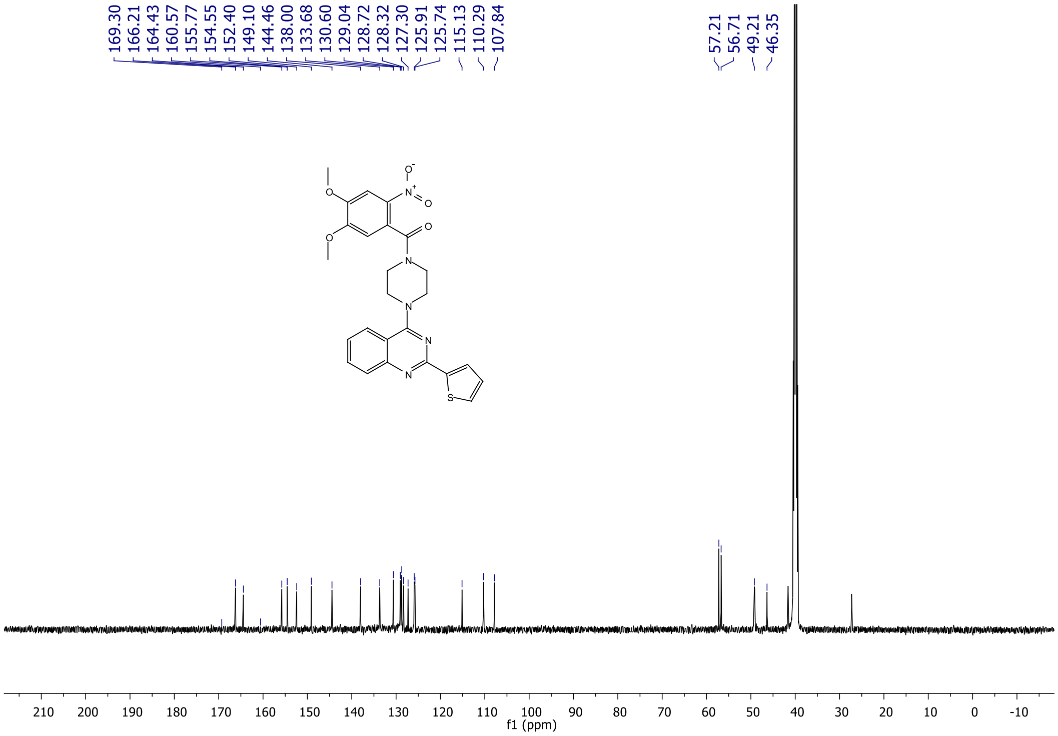

Compound **8w**: ^{13}C NMR (125 MHz, DMSO-d_6).

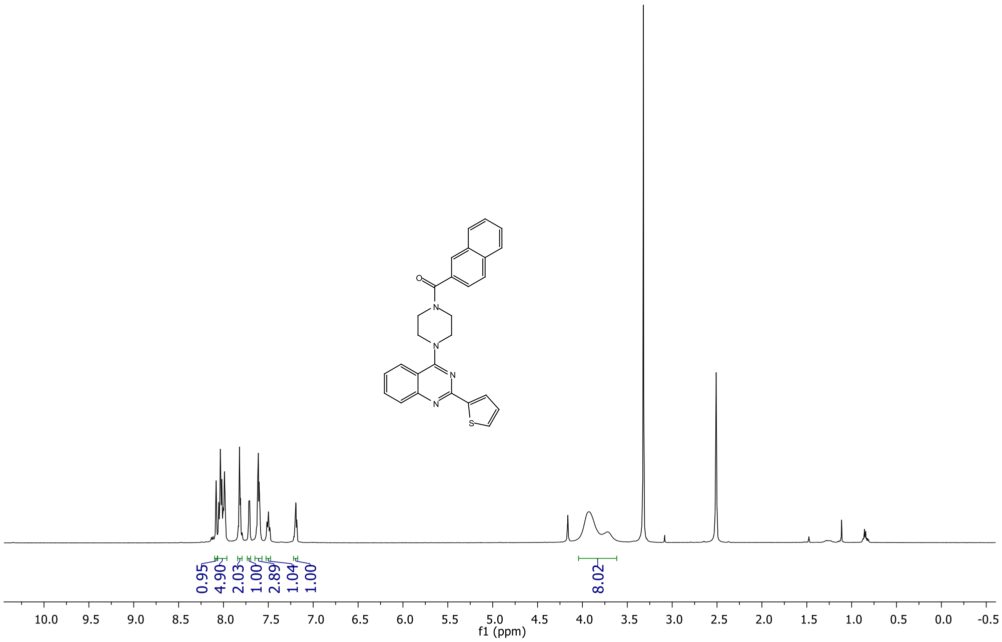

Compound **8x**: ^{1}H NMR (500 MHz, DMSO-d_6).

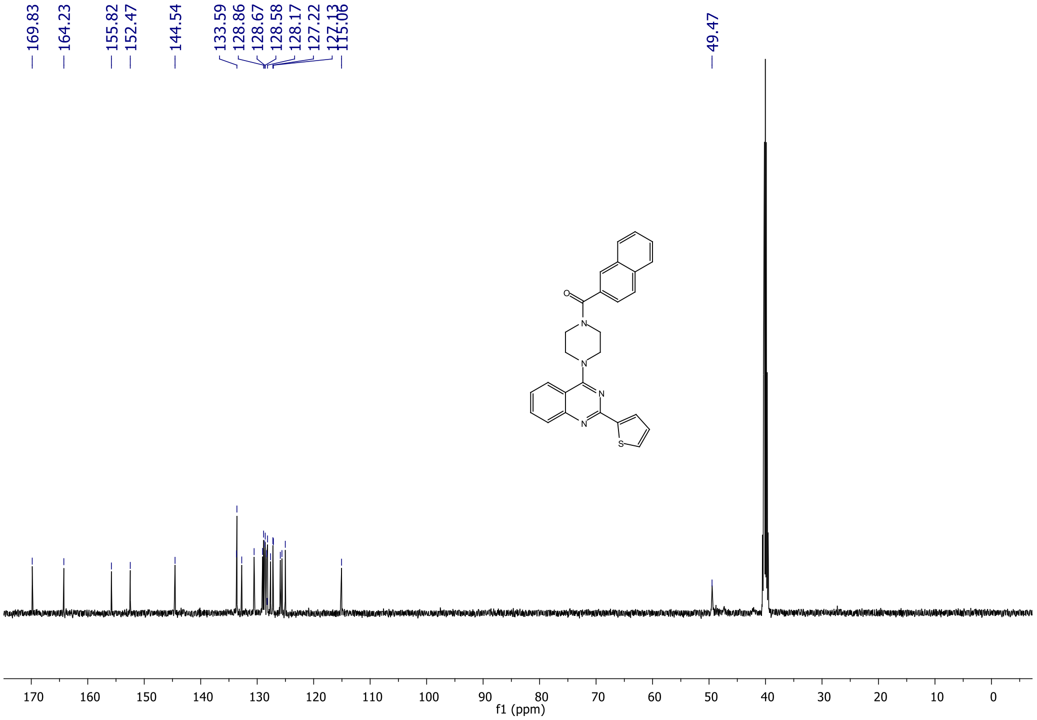

Compound **8x**: ^{13}C NMR (125 MHz, DMSO-d_6).

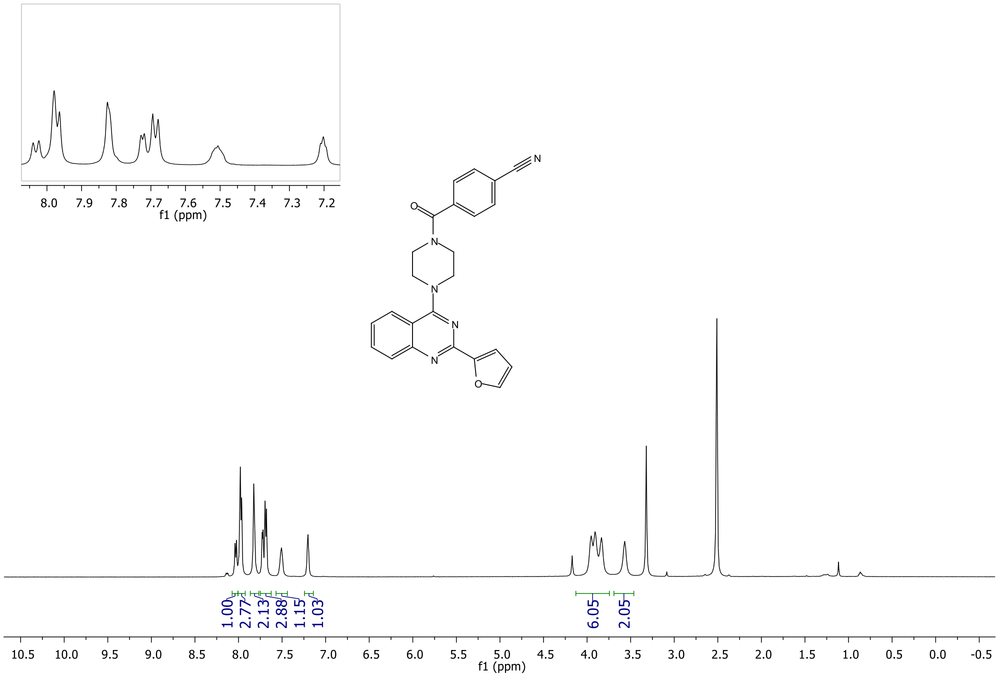

Compound **8y**: ^{1}H NMR (500 MHz, DMSO-d_6).

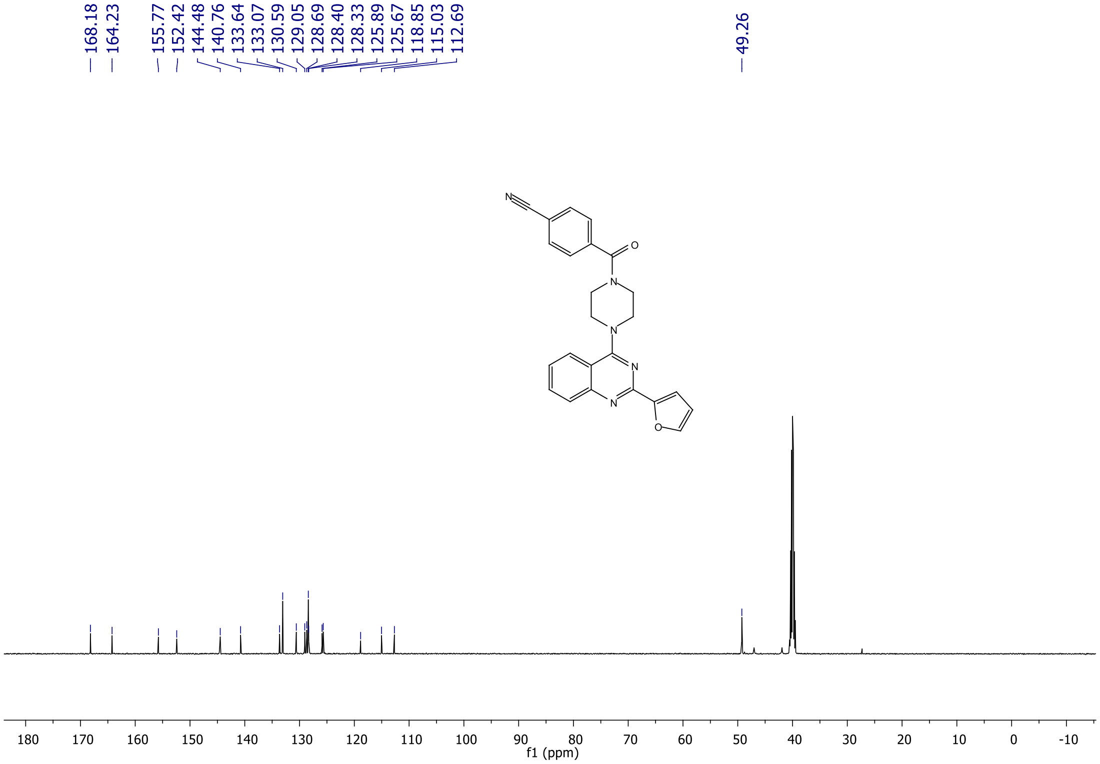

Compound **8y**: ^{13}C NMR (125 MHz, DMSO-d_6).

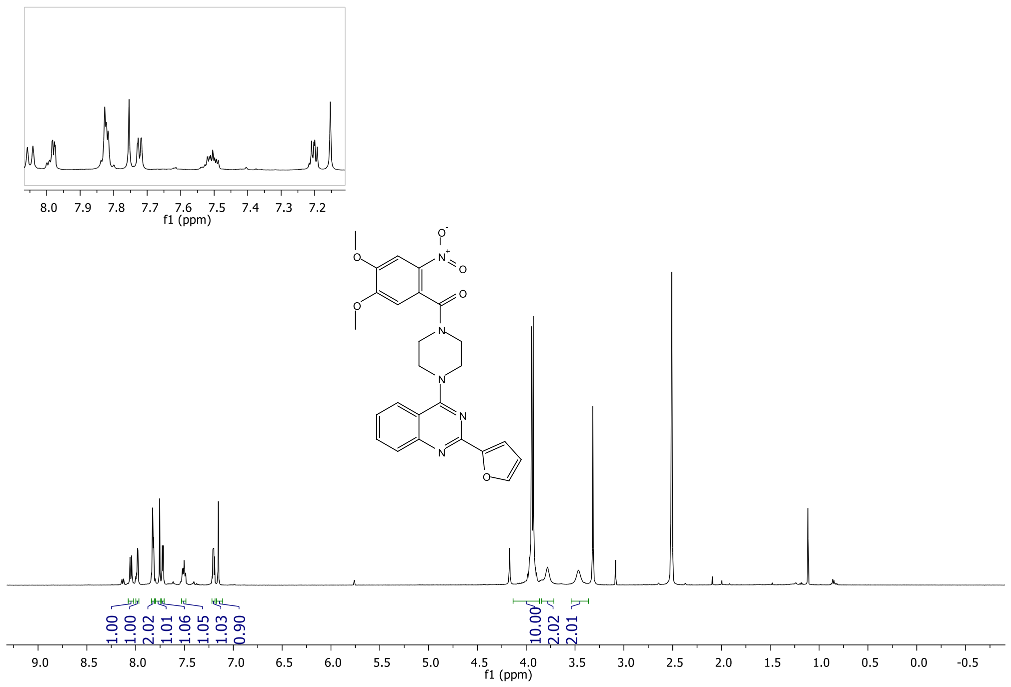

Compound **8z**: ^{1}H NMR (500 MHz, DMSO-d_6).

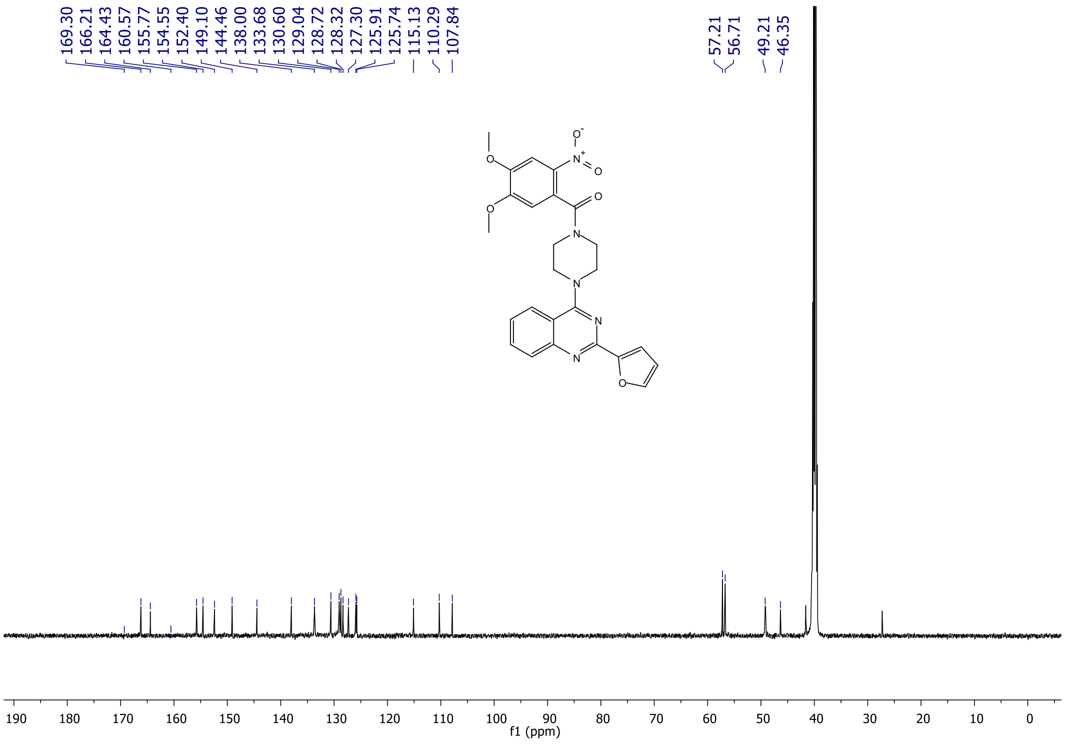

Compound **8z**: ^{13}C NMR (125 MHz, DMSO-d_6).
